# Genome-wide identification, characterization and evolutionary analysis of the *pyrroline-5-carboxylate synthetase* (*P5CS*), *succinic semialdehyde dehydrogenase* (*SSADH*), and *dehydrin* (*DHN*) genes in *Solanum lycopersicum* under drought stress

**DOI:** 10.1186/s12870-025-07057-w

**Published:** 2025-08-09

**Authors:** Amaal Maghraby, Mohamed Alzalaty

**Affiliations:** 1 Faculty of Science, Department of Botany and Microbiology, Cairo University, Giza, Egypt; 2 Agricultural Genetic Engineering Research Institute (AGERI), Agricultural Research Center (ARC), Giza, Egypt

**Keywords:** Bioinformatics, Phylogenetics, Chromosomal distribution, Gene duplication, Synteny

## Abstract

**Background:**

The *pyrroline-5-carboxylate synthetase* (*P5CS*), *succinic semialdehyde dehydrogenase* (*SSADH*), and *dehydrin (DHN*) genes are involved in plant drought response.

**Methods:**

A comprehensive bioinformatics approach was applied, including phylogenetic, structural, evolutionary, and functional analyses, as well as promoter, subcellular localization, and gene ontology assessments.

**Results:**

This is the first study to identify the *P5CS*, SSADH and *DHN* genes in *Solanum lycopersicum* via genome-wide analysis under drought stress. We identify 2 *P5CS*, 18 SSADH and 16 *DHN* genes in *S. lycopersicum*. The chromosomal distribution showed that *P5CS* genes were located on chromosomes 6 and 8, *SSADH* genes on chromosomes 1, 2, 3, 5, 6, 7, 8, 9, and 12, and *DHN* genes on chromosomes 1, 2, 3, 4, 5, 6, 7, 8, 9,10, and 12. The *Ka*/*Ks* ratios indicated that the *P5CS*, SSADH and *DHN* genes were influenced primarily by negative selection, which indicated that the *P5CS*, *SSADH* and *DHN* genes received strong environmental pressure during evolution.

The duplication time of the *P5CS* paralogous gene pairs was approximately 40.030 Mya. The duplication time of the *SSADH* paralogous gene pair ranged from approximately 31.495 to 45.966 Mya. The duplication time of the *DHN* paralogous gene pairs ranged from approximately 1.645 to 102.128 Mya. Synteny analysis of the *P5CS*, SSADH and *DHN* genes revealed collinearity orthologous relationships in *Solanum tuberosum* and *Arabidopsis thaliana* but no orthologs of the *P5CS*,* SSADH* and *DHN* genes with *Oryza sativa*. In addition, collinearity analysis revealed that 1 orthologous *P5CS* genes, 20 orthologous *SSADH* genes and 5 orthologous *DHN* genes were paired with those in *S. tuberosum*. Additionally, collinearity analysis revealed that 15 orthologous *SSADH* genes, 5 orthologous *DHN* gene and no orthologous *P5CS* genes, were paired with those in *A. thaliana*. The qRT–PCR results indicated that *P5CS* and *DHN* were upregulated, with fold changes of 2.39 and 1.23, respectively, whereas *SSADH* expression decreased with a fold change of 0.73.

**Conclusions:**

Our results provide comprehensive insights into the *P5CS*, *SSADH*, and *DHN* genes, including their protein structures and predicted interaction features. These findings offer valuable targets for tomato breeding programs aimed at developing stress-tolerant varieties under changing climate conditions.

**Supplementary Information:**

The online version contains supplementary material available at 10.1186/s12870-025-07057-w.

## Background

Drought occurs in most regions and has affected agricultural production more than any other natural hazard in the last 40 years. Climate change has led to drought in many areas of the world, increasing in severity, frequency and duration [1]. Drought resulted from a deficiency in precipitation for a long time. It may constitute a part of the normal climate cycle in many climate zones, but it can develop quickly through extreme heat and/or wind [2]. Drought is an abiotic stress that limits crop production worldwide. We need more studies to understand the genetic and molecular mechanisms of drought tolerance in tomatoes [3]. Under drought stress, plant cells undergo several critical physiological and molecular alterations that severely impair cellular function. According to Haghpanah et al. [4], drought stress elicits six major consequences: osmotic and dehydration stress, loss of cellular turgidity, dysfunction of plasma and endosomal membranes, metabolic inhibition, energy depletion, chloroplast malfunction, and oxidative stress. These effects collectively compromise growth and productivity. Investigating how these interconnected disruptions trigger specific gene expression and signaling pathways is essential for understanding the adaptive mechanisms plants employ to survive under water-deficit conditions [4].

Plants have evolved complex molecular mechanisms to maintain cellular homeostasis under drought conditions [5]. Among the key genetic components involved in drought tolerance are the *Δ¹-pyrroline-5-carboxylate synthase* (*P5CS*) [6], *succinic semialdehyde dehydrogenase* (*SSADH*) [7], and *dehydrin* (*DHN*) [8] genes, which contribute to distinct but interconnected protective responses. These genes collectively function in osmoprotection, oxidative stress mitigation, and stabilization of cellular structures under water-deficit conditions, and their coordinated expression has been frequently observed in drought-tolerant cultivars. Understanding their integrated roles offers a valuable framework for improving stress resilience in crops such as tomato [6, 8].

*P5CS* is a critical enzyme in the biosynthesis of proline, a compatible solute that accumulates in plant cells during drought and contributes to osmotic adjustment and membrane stabilization [6]. The upregulation of *P5CS* under drought stress has been reported in species such as *Eugenia uniflora* [9], where proline accumulation protects against oxidative stress and membrane damage [10]. Two isoforms of *P5CS* have been identified: *P5CS1*, which is primarily induced during stress conditions, and *P5CS2*, which is more involved in development and growth [11]. Overexpression of *P5CS* in *A. thaliana* resulted in improved drought tolerance, as evidenced by increased root length, higher survival rates, and reduced membrane injury under water stress [12].

*SSADH* operates within the γ-aminobutyric acid (GABA) shunt pathway, converting succinic semialdehyde into succinate, thereby linking stress response and primary metabolism through the tricarboxylic acid (TCA) cycle [7]. Together with GABA transaminase (GABA-T), *SSADH* contributes to cellular redox balance and energy supply under abiotic stress conditions [13]. The GABA shunt is regulated by glutamate decarboxylase (GAD) in the cytosol and mitochondrial enzymes such as GABA-T (POP2) and *SSADH*. SSADH-deficient mutants in *A*. *thaliana* show increased accumulation of reactive oxygen intermediates and susceptibility to oxidative damage, highlighting the gene’s role in ROS detoxification and stress tolerance [14].

*DHN*, belonging to the *Late Embryogenesis Abundant* (*LEA*) protein gene family, are known for their protective function under drought, salinity, and cold stress [8]. These proteins are highly hydrophilic and function by stabilizing macromolecules and cellular membranes through molecular shielding and water binding [15, 16]. *DHN* expression is commonly induced in response to abiotic stresses, particularly those that lead to cellular dehydration. For instance, *DHN1* expression is significantly upregulated under drought in grapevine [15], and transgenic *A*. *thaliana* lines overexpressing *DHNs* exhibit enhanced tolerance to osmotic stress [17]. The presence of conserved K-segments within *DHNs* contributes to their functional capacity to bind ions and interact with membranes [18]. Their broad expression patterns across tissues and responsiveness to environmental triggers such as salinity and temperature suggest their central role in the adaptive stress response in various crops, including pepper [19].

Despite extensive studies on *P5CS*, *SSADH*, and *DHN* genes in various plant species, genome-wide identification and detailed functional analysis in *S*. *lycopersicum* are still not studied. This study aims to fill these gaps by performing a genome-wide analysis of *P5CS*, *SSADH*, and *DHN* gene families in *S*. *lycopersicum*, investigating their Phylogenetic relationships, chromosomal distribution, evolutionary analysis, synteny analysis, Conserved domain, conserved motif, gene structure analyses, promoters analysis, Subcellular localization, nuclear localization signal, transmembrane helices, phosphorylation sites analysis, Three-dimensional (3-D) structure prediction, functional interaction network analysis, Prediction of miRNAs targeting genes, Gene Ontology enrichment, offer novel clues into the structure, interaction, and functional analysis of these gene families in tomato’s drought response. These findings contribute a unique dataset and framework for future functional genomics studies in *Solanaceae*.

## Methods

### Identification of the *P5CS*, *SSADH* and *DHN* genes in *Solanum lycopersicum*

The annotated genome assemblies of *S. lycopersicum* (genome version: ITAG5.0, Phytozome ID: Slycopersicum_796), *S. tuberosum* (version: v6.1, Phytozome ID: Stuberosum_686), *O. sativa* (IRGSP-1.0, release 56, Phytozome ID: Oryza_sativa.IRGSP-1.0.56), and *A. thaliana* (Araport11, Phytozome ID: Athaliana_167) were retrieved from the Phytozome database (https://phytozome-next.jgi.doe.gov/) [20]. To identify gene family members in *S. lycopersicum*, we first retrieved well-characterized protein sequences of the studied genes from model plants (*A. thaliana*, *O. sativa*, and *S. tuberosum*) using the NCBI database (https://www.ncbi.nlm.nih.gov/) [21]. These sequences were used as query inputs for BLASTP searches against the *S. lycopersicum* proteome in Phytozome, applying a stringent E-value threshold (≤ 1e-30) to ensure specificity. Subsequent iterative BLASTP searches were performed using each identified protein to capture additional homologs within the gene families. Final candidate genes were validated based on conserved domain architectures (e.g., PF00257 for DHNs, PF00171 for SSADH, and PF00696/PF01431 for P5CS) and consistent protein features. All identified genes and their corresponding accession numbers are listed in Table **S1** (Online Resource SI 1).

### Characterization of the *P5CS*, *SSADH* and *DHN* proteins in *S. lycopersicum*

Circoletto (http://tools.bat.infspire.org/circoletto/) [22] visualized the sequence identity of the studied proteins. The physical and chemical properties of the Studied proteins, including the molecular weight, total number of negatively charged residues (Asp + Glu), total number of positively charged residues (Arg + Lys), isoelectric point, total number of atoms, grand average hydropathicity (GRAVY) and instability, were computed using the ExPASy ProtParam Tool (https://web.expasy.org/protparam/) [23].

### Phylogenetic, chromosomal distribution, evolutionary analysis and synteny analysis of the *P5CS*, *SSADH* and *DHN* genes in *S. lycopersicum*

Multiple sequence alignments of the studied proteins from were performed via the MUltiple Sequence Comparison by Log-Expectation (MUSCLE) method. Molecular evolutionary genetic analysis (MEGA-11) [24] of the studied proteins were subsequently conducted on a phylogenetic tree with a maximum likelihood. The iTOL online website (https://itol.embl.de/) [25] was used to adjust and visualize the tree of the studied proteins.

Based on the chromosomal location information of the studied genes, a chromosomal localization map was generated using TBtools [26]. The resulting figure illustrates the distribution of these genes across the different chromosomes.

The rates of synonymous (*KSs*) and nonsynonymous (*KAs*) substitutions of the studied genes were calculated via TBtools [26] to investigate selection pressure [27]. The divergence time of the studied gene pairs was estimated using the synonymous mutation rate of substitutions per synonymous site per million years ago (Mya) as follows: “T = Ks/2λ × 10^−6^ (λ = 6.5 × 10^−9^)” [28]. Paralogous genes of the studied genes were identified if the alignment covered **≥** 70% of the longer gene and if the aligned region was **≥** 70% [29]; additionally, the genes were identified by a gene duplication wizard [24]. Collinearity analysis of the studied paralogous gene pair was visualized as a Circos plot through TBtools [26].

TBtools [26] were used to determine the syntenic relationships of the studied genes in *S. lycopersicum* with those in *Solanum tuberosum*,* O. sativa* and *A. thaliana*.

### Conserved domain, conserved motif, gene structure analyses and promoters of the *P5CS*, *SSADH* and *DHN* genes in *S. lycopersicum*

The NCBI conserved domain tool (https://www.ncbi.nlm.nih.gov/Structure/cdd/wrpsb.cgi) [30] was used to search against the Pfam v34.0–19,178 PSSM database for the studied proteins. The InterPro tool **(**https://www.ebi.ac.uk/interpro/) [31] was used to analyze the domains of the studied proteins.

MEME 5.5.5 (https://meme-suite.org/) [32] was used to compute the conserved motifs of the studied proteins.

The gene structures of studied were retrieved from the GFF *S. lycopersicum* genome file and subsequently illustrated using TBtools [26].

The 1500 bp upstream promoter sequences (relative to the transcription start site, TSS) were retrieved for each of the studied genes from the *S. lycopersicum* genome and downloaded via the Phytozome database (https://phytozome-next.jgi.doe.gov/) [20]. Cis-regulatory elements (CREs) within these promoter regions were identified using the PlantCARE database (https://bioinformatics.psb.ugent.be/webtools/plantcare/html/) [33]. A graphical representation of the detected CREs was generated using TBtools [26].

### Subcellular localization, nuclear localization signal, transmembrane helices and phosphorylation sites of the *P5CS*,* SSADH *and* DHN *genes in* S. lycopersicum*

Subcellular localization predictor (CELLO) version 2.5 (http://cello.life.nctu.edu.tw/) [34] was used to predict the subcellular localization of the studied proteins, and the results were visualized via TBtools [26]. NLSDB (http://www.moseslab.csb.utoronto.ca/NLStradamus/) [35] was used to search for nuclear localization signal potentials of the studied proteins. The TMHMM server version 2.0 (https://services.healthtech.dtu.dk/services/TMHMM-2.0/) [36] was used to confirm the presence of transmembrane helical domains (TMs) in the studied proteins. The NetPhos 3.1 server (https://services.healthtech.dtu.dk/services/NetPhos-3.1/) [37] was used to predict the phosphorylation sites of the studied proteins.

### Three-dimensional (3-D) structure prediction and functional interaction network analysis of the *P5CS*, *SSADH* and *DHN* proteins in *S. lycopersicum*

The 3D structures of the studied proteins were predicted using the Swiss-Model server (https://swissmodel.expasy.org/) [38]. To identify structural analogs and assess structural similarity, the I-TASSER server (https://zhanggroup.org/I-TASSER/) [39] was used, which provided C-score, TM-score, and RMSD values for each predicted model based on structural templates in the PDB database. The STRING database (https://string-db.org/) [40] was used to determine the physical interaction network between the studied proteins.

### Prediction of miRNAs targeting the *P5CS*, *SSADH* and *DHN* genes in *S. lycopersicum*

The psRNATarget database (https://www.zhaolab.org/psRNATarget/analysis?function=3) [41] and miRBase (https://www.mirbase.org/) [42] were used to predict miRNAs of the studied genes. IPKnot (https://ws.sato-lab.org/rtips/ipknot++/) [43] was used to predict RNA secondary structures with pseudoknots for the studied genes.

### Gene Ontology enrichment and functional relationship analysis of the *P5CS*, *SSADH* and *DHN* genes in *S. lycopersicum*

Gene Ontology (GO) annotation analysis was performed by submitting all the studied gene sequences to the eggNOG database (https://bio.tools/eggnog-mapper-v2) [44] and Phytozome database (https://phytozome-next.jgi.doe.gov/) [20]. The GO annotation data were processed in SRPLOT (http://www.bioinformatics.com.cn/plot_basic_GOplot_chord_plot_085_en) [45] to construct the gene ontology chord for the functional relationships of the studied genes. ShinyGO 0.77 (http://bioinformatics.sdstate.edu/go77/) [46] was used for Gene Ontology enrichment analysis of the studied genes.

### Cultivation of tomatoes and drought treatment

These experiments were conducted in the Department of Botany and Microbiology, Faculty of Science at Cairo University. The Sagatan F1 hybrid tomato seeds used in this study were obtained from the Agricultural Research Center (ARC), Giza, Egypt. A completely randomized design (CRD) was used. Thirty seeds were sown in small plastic pots and grown under controlled growth room conditions (25 °C, 16/8 h light/dark cycle) for 14 days. The plants were then divided into two groups: control and drought-stressed. Drought stress was imposed by withholding water for 2, 6, and 12 h, while control plants received Hoagland’s nutrient solution. Leaf tissues were harvested after 2, 6, and 12 h of treatment. For RNA extraction and sequencing, three biological replicates were collected from each group, with each replicate consisting of a single plant.

### RNA isolation, qRT‒PCR expression analysis and sequencing of the *P5CS*, *SSADH* and *DHN* genes in *S. lycopersicum*

In this study, Full-length cDNA sequences were retrieved from the *S. lycopersicum* genome (version: Slycopersicum_796_ITAG5.0) available in the Phytozome database (https://phytozome-next.jgi.doe.gov/) [20]. Gene-specific primers were designed using NCBI Primer-BLAST to ensure specificity. Primer properties such as GC content, melting temperature, and secondary structure were analyzed using the OligoAnalyzer tool (https://www.idtdna.com/pages/tools/oligoanalyzer). For *P5CS* the forward primer was CCAAAGCTAGCTCCCTGCTC and the reverse primer was AAAGTTTGCATGGAACCGGAG. For *SSADH*, the forward primer was TGAGAATTTCACTCATCGTTGC and the reverse primer was ACACTCCGTCTCTTCTCACC. For *DHN*, the forward primer was GTCTTCTTATGCCCGCCACC and the reverse primer was ACCGTTTACTTTTTCTAGCGGGTA). These primers target a single gene isoform within each family to monitor gene expression under drought stress. Total RNA was isolated from leaf tissues using a GeneTireX kit and treated with RNase-free DNase I (Thermo Scientific, Lithuania). First-strand cDNA was synthesized using 2 µg of total RNA and a Grisp reverse transcription kit (https://grisp.pt/). qRT-PCR was conducted using a CFX Connect Real-Time PCR System (Bio-Rad, Singapore) with the following conditions: 95 °C for 10 min, followed by 40 cycles of 94 °C for 10 s, 58 °C for 20 s, and 72 °C for 30 s. A melt curve analysis was performed (65–95 °C, 0.5 °C increments) [47]. The TIP41 gene was used as an internal reference the forward primer was ATGGAGTTTTTGAGTCTTCTGC and the reverse primer was GCTGCGTTTCTGGCTTAGG) [48], and expression levels were calculated using the 2^-ΔΔCt^ method [49]. All qRT-PCRs for each sample were done in three biological and three technical duplicates. Melting curve examination of the amplicons verified the specificity of the PCR reaction.

## Results

### Identification of the *P5CS*, *SSADH* and *DHN* genes in *S. lycopersicum*

A total of 2 *P5CS*, 18 *SSADH* and 16 *DHN* candidate genes were identified from the *S. lycopersicum* genome and were named according to their chromosomal positions from *SlP5CS-1* to *SlP5CS-2*, *SlSSADH-1* to *SlSSADH-18* and *SlDHN-1* to *SlDHN-16* genes (Table S1 Online Resource SI 1).

### Characterization of the *P5CS*, *SSADH* and *DHN* proteins in *S. lycopersicum*

**Fig. 1:**
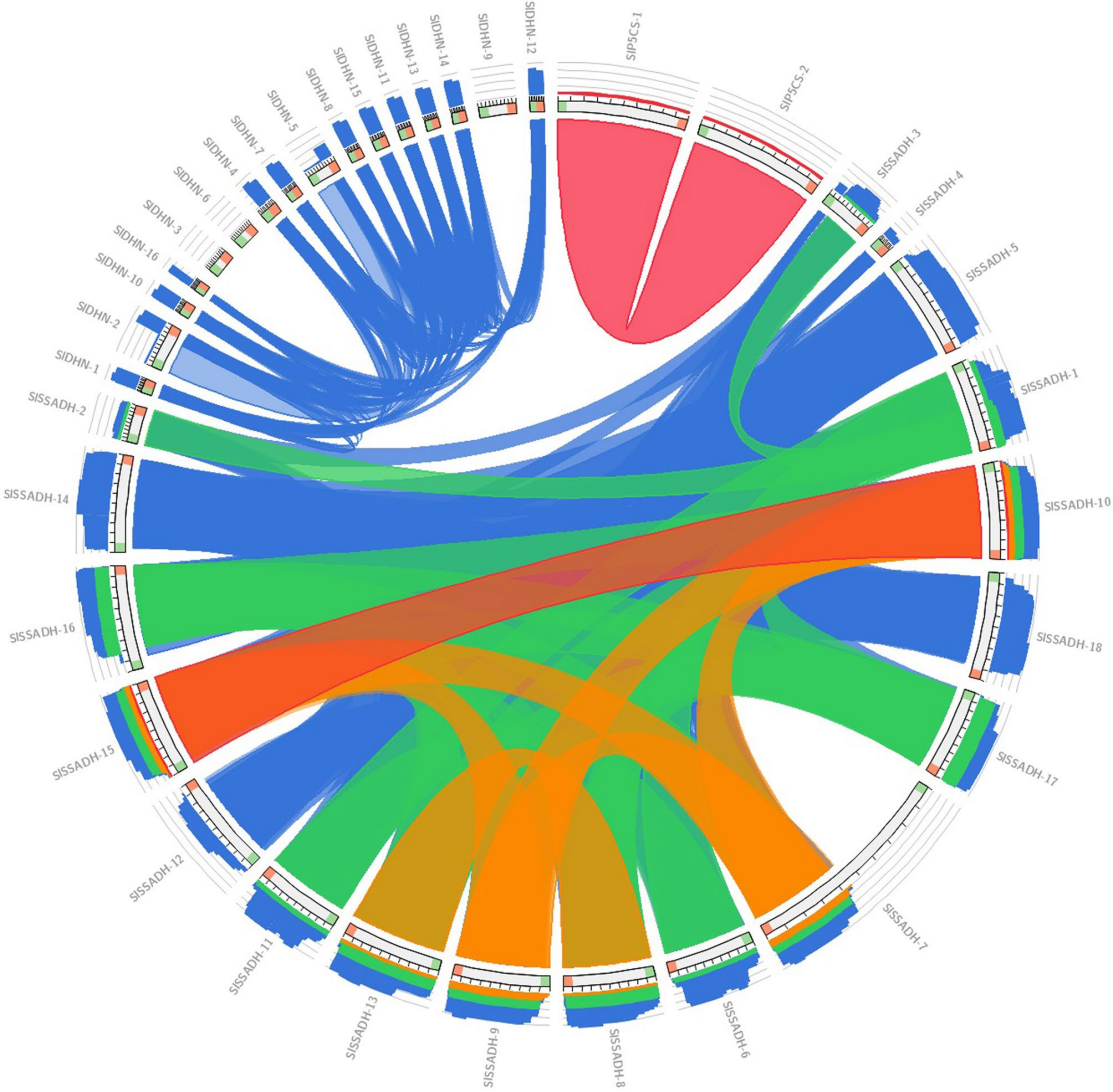
Sequence identity comparison of 2 P5CS, 18 SSADH, and 16 DHN proteins based on color-coded E-value similarity. Blue indicates identity ≤ 70%, green ≤ 80%, orange ≤ 90%, and red otherwise. The alignment visualization highlights conserved and variable regions among the gene family members

The sequence identities of studied proteins are shown by the color-by-E-value ratio (blue, ≤ 70%; green, ≤ 80%; orange, ≤ 90% and red otherwise), as shown in Fig. 1.

Analysis of protein physical and chemical properties revealed that the lengths of the P5CS family amino acids ranged from 717 (SlP5CS-1) to 720 (SlP5CS-2). The length of the amino acids in the SSADH family ranged from 85 (SlSSADH-4) to 1206 (SlSSADH-7). The length of the DHN family amino acids ranged from 61 (SlDHN-16) to 249 (SlDHN-2). The molecular weights (MWs) of P5CS ranged from 77478.64 (SlP5CS-1) to 78008.21 (SlP5CS-2). The molecular weights of SSADH ranged from 9136.69 (SlSSADH-4) to 136201.79 (SlSSADH-7). The molecular weights of the DHNs ranged from 7156.18 (SlDHN-16) to 25363.8 (SlDHN-2). The isoelectric point (pI) of P5CS ranged from 5.58 (SlP5CS-1) to 6.14 (SlP5CS-2). The isoelectric point of SSADH ranged from 5.33 (SlSSADH-8) to 9.26 (SlSSADH-2). The isoelectric point of DHN ranged from 5.12 (SlDHN-9) to 9.95 (SlDHN-4). The total number of atoms in P5CS ranged from 11,016 (SlP5CS-1) to 11,080 (SlP5CS-2). The total number of atoms in SSADH ranged from 1323 (SlSSADH-4) to 19,112 (SlSSADH-7). The total number of atoms in the DHN ranged from 1014 (SlDHN-16) to 3509 (SlDHN-2). The average hydropathicity value (GRAVY) of P5CS ranged from − 0.032 (SlP5CS-2) to −0.02 (SlP5CS-1). The average hydropathicity value of SSADH ranged from − 0.173 (SlSSADH-12) to 0.173 (SlSSADH-4). The average hydropathicity of DHN ranged from − 1.744 (SlDHN-8) to −0.838 (SlDHN-2) (Table S1 Online Resource SI 1).

### Phylogenetic, chromosomal distribution, evolutionary analysis and synteny analysis of the *P5CS*, *SSADH* and *DHN* genes in *S. lycopersicum*

**Fig. 2:**
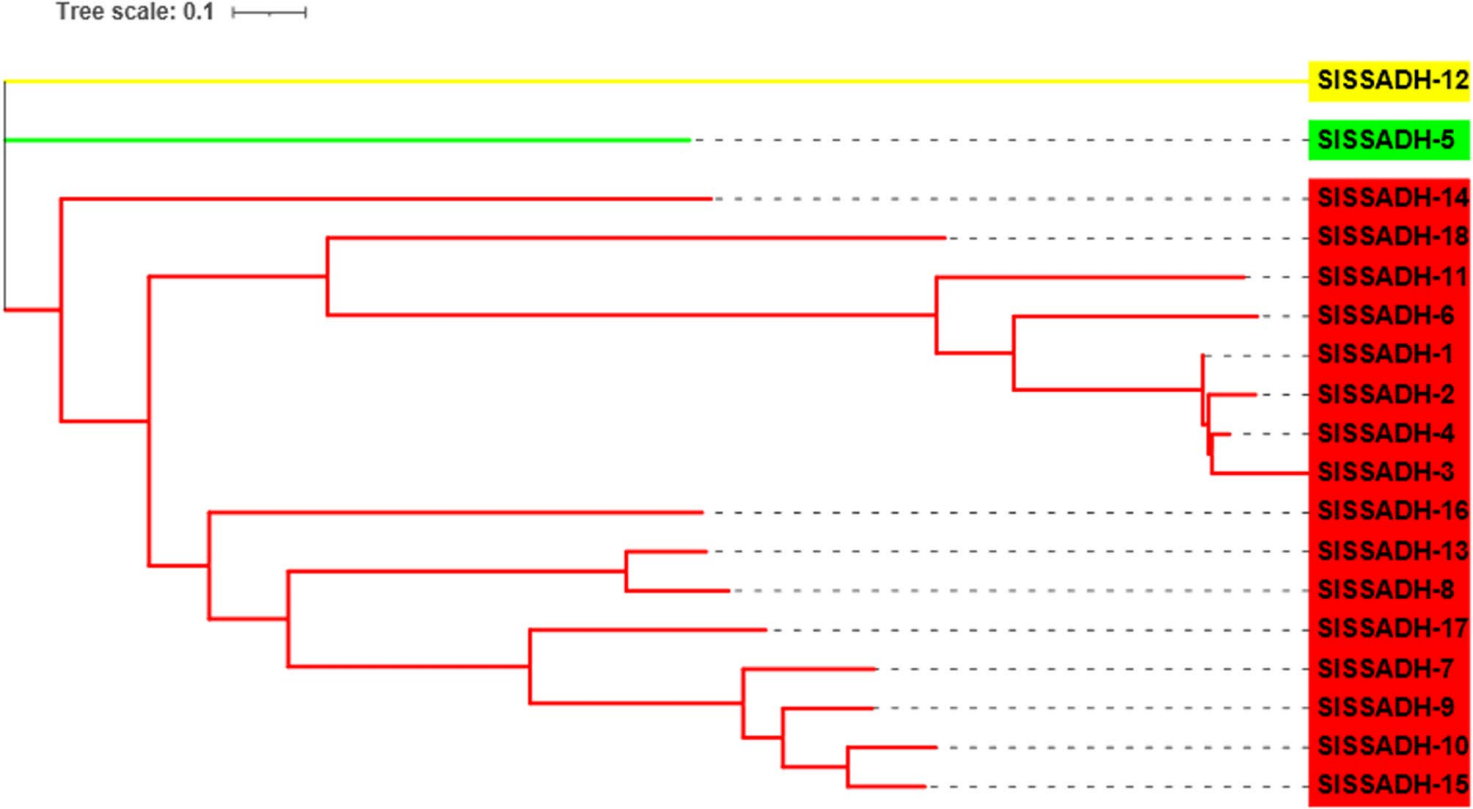
Maximum likelihood phylogenetic tree of 18 SlSSADH protein sequences from *S. lycopersicum*, constructed using MEGA-11

**Fig. 3:**
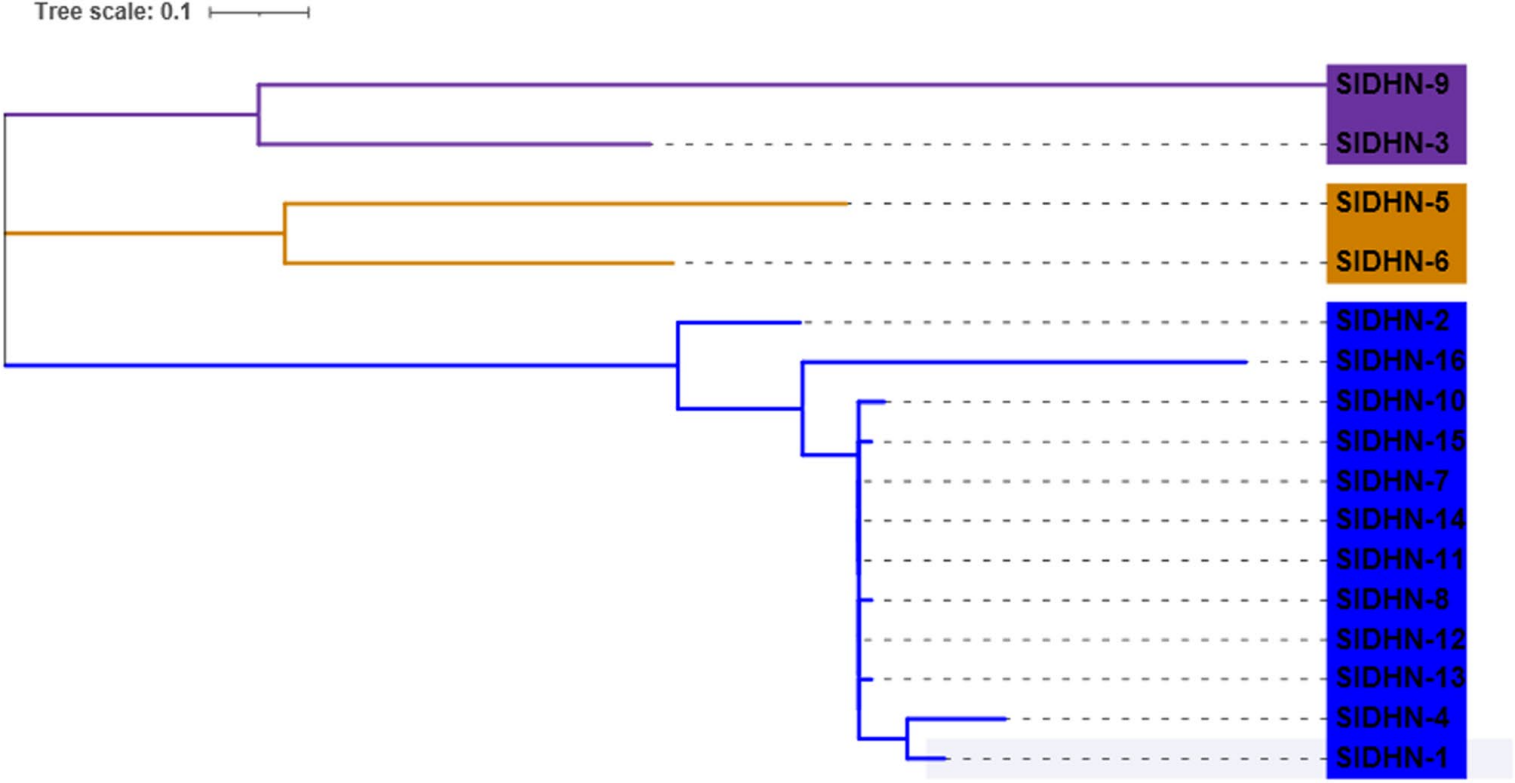
Maximum likelihood phylogenetic tree of 16 SlDHN protein sequences from *S. lycopersicum*, constructed using MEGA-11

A maximum likelihood phylogenetic tree was constructed to analyze the possible evolutionary history of SSADH and DHN protein sequences. In the resulting phylogenetic tree, the 18 SlSSADH and 16 SlDHN proteins were classified into three distinct clades **(**Figs. 2 and 3**)**.

**Fig. 4:**
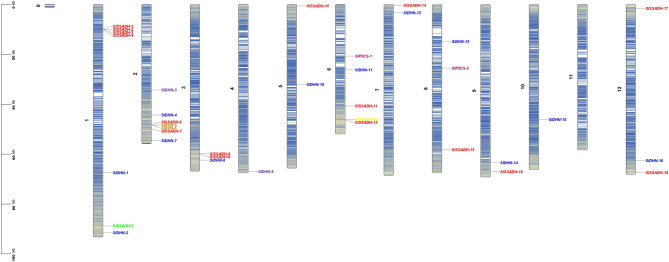
Chromosomal distribution of 2 *P5CS*, 18 *SSADH*, and 16 *DHN* genes in the *S. lycopersicum* genome

Based on the information available on the Phytozome-13 website [20], the studied genes were physically drawn on the chromosomes in the *S. lycopersicum* genome. Two *P5CS* genes were found on chromosomes 6 and 8. Five *SSADH* genes were found on chromosome 1, 2 *SSADH* genes on chromosome 2, 2 *SSADH* genes on chromosome 3, 1 *SSADH* gene on chromosome 5, 3 *SSADH* genes on chromosome 6, 1 *SSADH* gene on chromosome 7, 1 *SSADH* gene on chromosome 8, 1 *SSADH* gene on chromosome 9, and 2 *SSADH* genes on chromosome 12. Two *DHN* genes were found on chromosome 1, five *DHN* genes were found on chromosome 2, while chromosomes 3, 4, 5, 6, 7, 8, 9, 10, and 12 have one *DHN* gene **(**Fig. 4**)**.

The evolutionary pressure of the studied genes was investigated by calculating the nonsynonymous (*KAs*)/synonymous ratio (*KSs*). A *Ka*/*Ks* ratio exceeding 1 suggested positive selection of accelerated evolution, a *Ka*/*Ks* ratio less than 1 suggested negative selection [50], and a *KAs*/*KSs* ratio equal to 1 indicated neutral selection [51]. The evolutionary pressure results showed that the *Ka*/*Ks* ratios of the studied paralogous pairs were less than 1, which indicates that the studied genes were influenced primarily by negative selection, suggesting that the studied genes received strong environmental pressure during evolution and did not experience significant functional differences during evolution (Table 1).

**Table 1:** Paralogous pairs of *P5CS*,* SSADH and DHN* genes and their *Ka*/*Ks* ratios

locus 1	locus 2	Ka	Ks	Ka/Ks	Time
SlP5CS-1	SlP5CS-2	0.082946	0.525195	0.157934	40.03011326
SlSSADH-3	SlSSADH-4	0.277503	0.413216	0.67157	31.49511041
SlSSADH-10	SlSSADH-15	0.113109	0.600983	0.188207	45.8066246
SlSSADH-13	SlSSADH-8	0.114247	0.603082	0.189438	45.96659601
SlDHN-5	SlDHN-6	0.339114	1.339928	0.253084	102.1286229
SlDHN-4	SlDHN-1	0.046869	0.179782	0.260697	13.70292634
SlDHN-10	SlDHN-15	0.016484	0.021584	0.763714	1.64513898
SlDHN-11	SlDHN-8	0.005435	0.044457	0.122247	3.38852572
SlDHN-12	SlDHN-13	0.005445	0.04413	0.123377	3.363595657

**Fig. 5:**
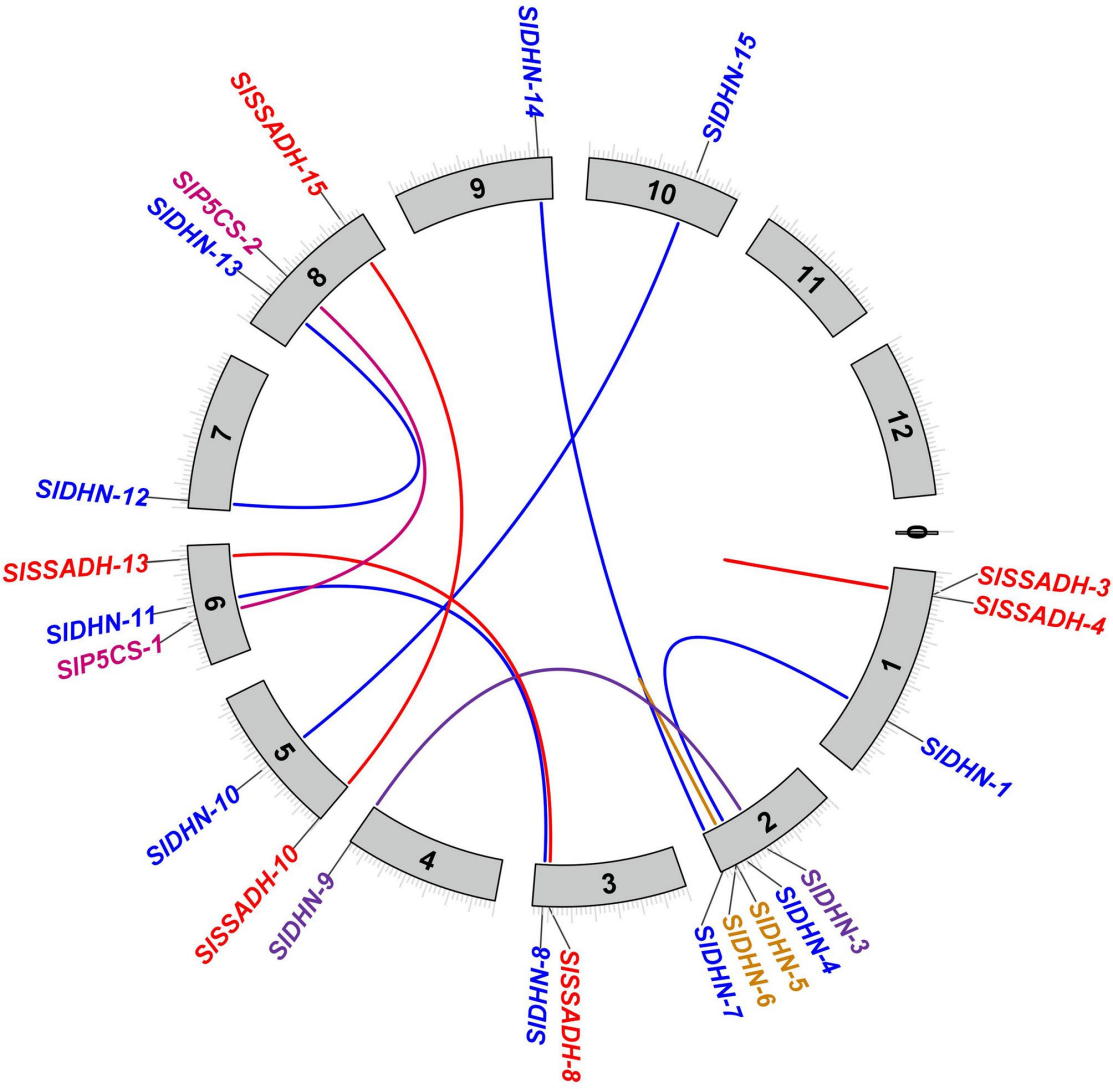
Duplication events among *P5CS*, *SSADH*, and *DHN* gene families in *S. lycopersicum*. The continuous lines indicate paralogous gene pairs resulting from duplication events. The duplication times vary across gene families, reflecting their evolutionary history

The duplication time of the *P5CS* paralogous gene pairs was approximately 40.030 Mya. The duplication time of the *SSADH* paralogous gene pair ranged from approximately 31.495 to 45.966 Mya. The duplications time of the *DHN* paralogous gene pairs ranged from approximately 1.645 to 102.128 Mya **(**Fig. 5).

**Fig. 6:**
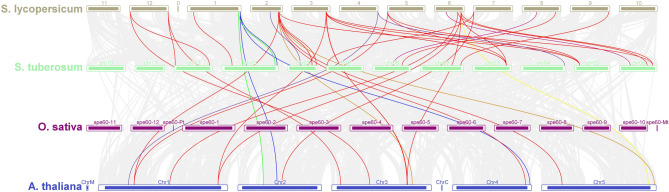
Interspecies collinearity analysis of *P5CS*, *SSADH*, and *DHN* genes between *S. lycopersicum* and three other species (*S. tuberosum*, *O. sativa*, and *A. thaliana*). Strong orthologous gene pairs were identified between *S. lycopersicum*, *S. tuberosum*, and *A. thaliana*, while no orthologs were detected in *O. sativa*. The number of orthologous gene pairs varied among gene families and species

The studied genes were analyzed for interspecies collinearity to determine the orthologous relationships of *S. lycopersicum* with *S. tuberosum*,* O. sativa* and *A. thaliana*. Collinearity analysis revealed robust orthologs genes among *S. lycopersicum* compared with *S. tuberosum* and *A. thaliana*,* whereas* no orthologs genes were detected in *O. sativa*. In addition, collinearity analysis revealed that 1 orthologous *P5CS* genes, 20 orthologous *SSADH* genes and 5 orthologous *DHN* genes were paired with those in *S. tuberosum*. Additionally, collinearity analysis revealed that 15 orthologous *SSADH* genes, 5 orthologous *DHN* genes, and no orthologous *P5CS* genes, were paired with those in *A. thaliana* **(**Fig. 6 and Table S2 Online Resource SI 1).

### Conserved domain, conserved motif, gene structure analyses and promoters of the *P5CS*, *SSADH* and *DHN* genes in *S. lycopersicum*

**Fig. 7:**
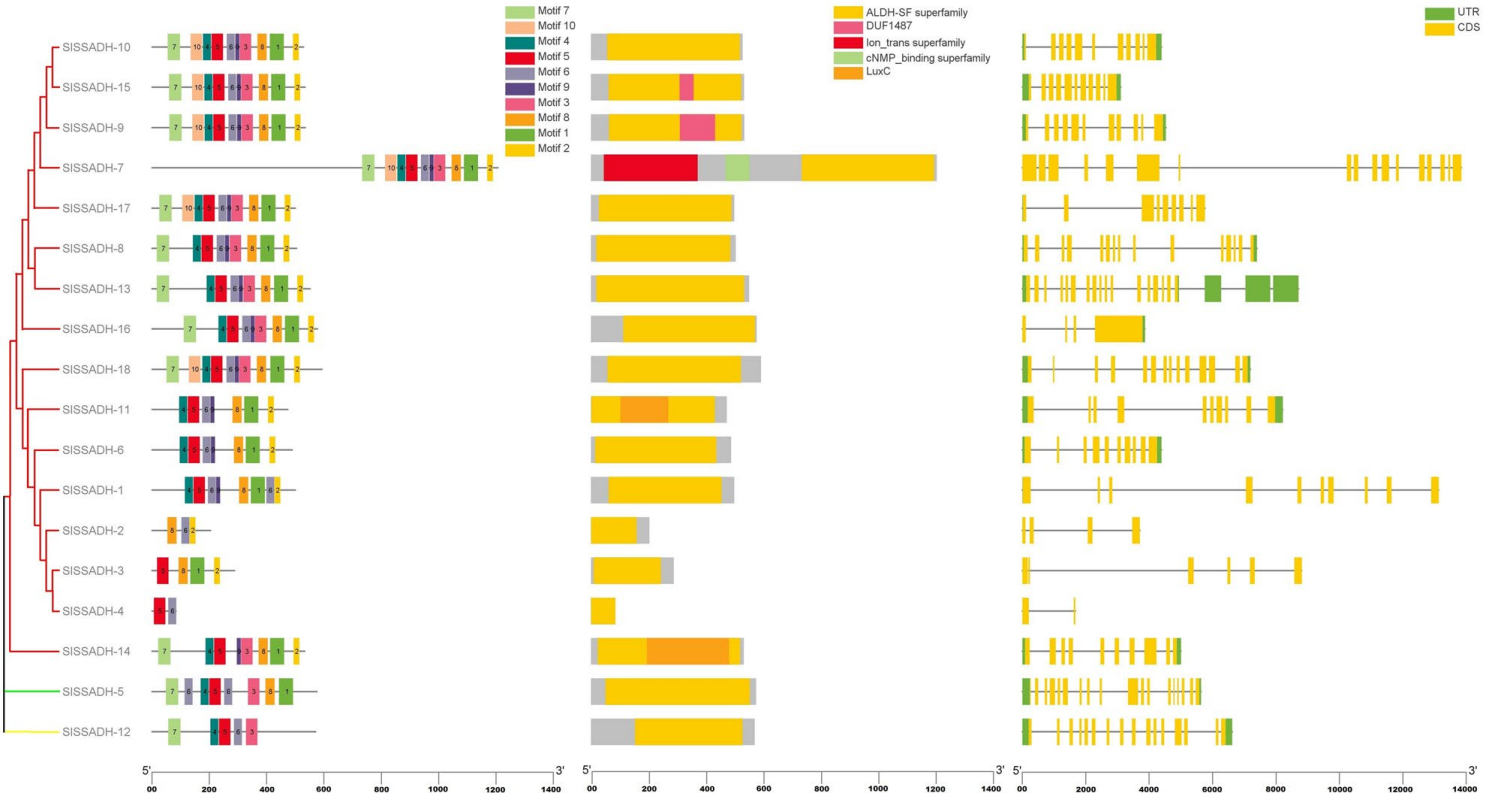
SSADH proteins. **a** Rectangular phylogenetic tree. **b** Conserved motifs were predicted using MEME. **c** Protein domains. **d** Gene structure

**Fig. 8:**
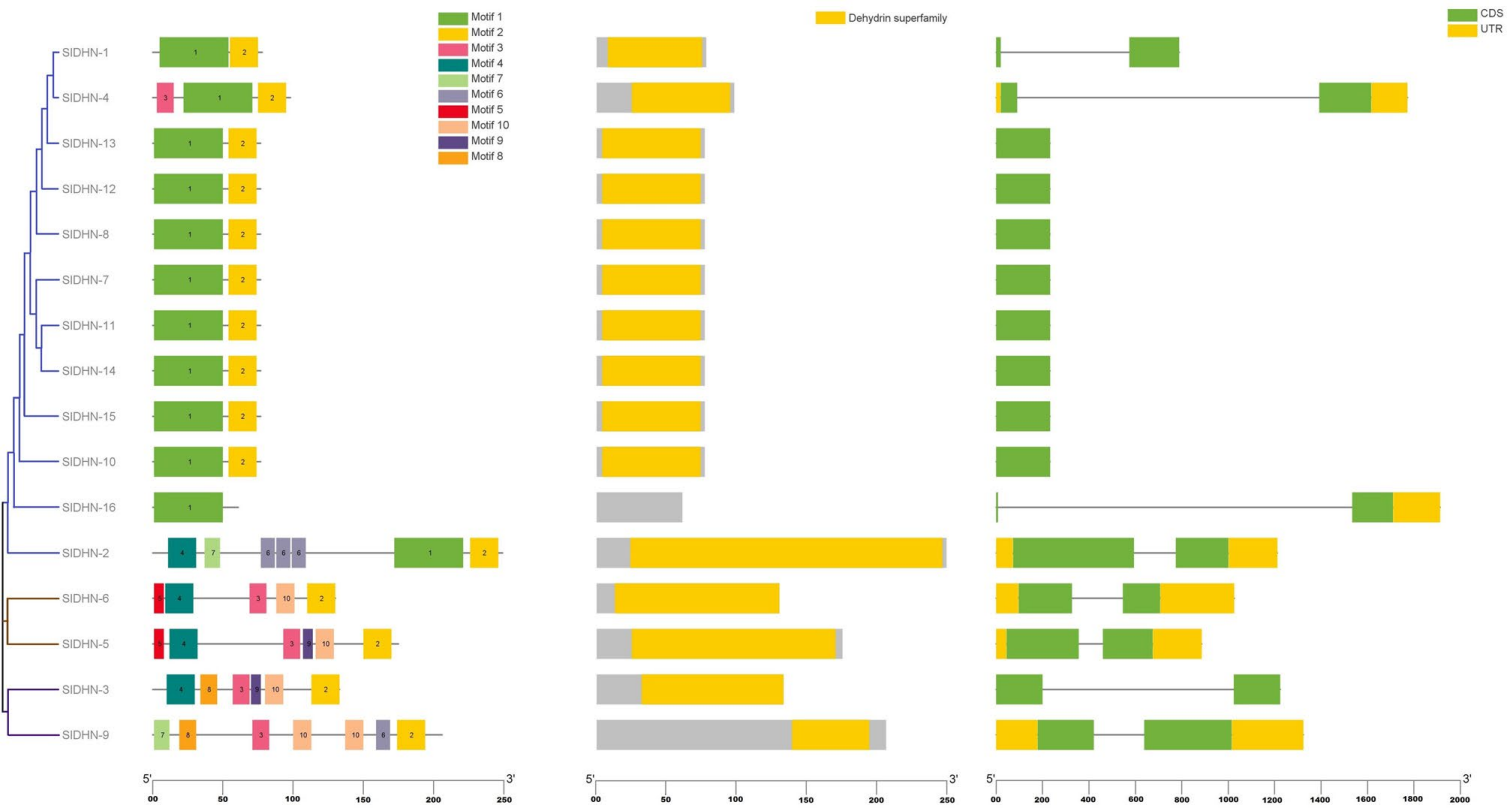
DHN proteins. **a** Rectangular phylogenetic tree. **b** Conserved motifs were predicted using MEME. **c** Protein domains. **d** Gene structure

Domain analysis was carried out for all studied proteins, and domain analysis confirmed the presence of the AAK superfamily domain **(**Fig. S1 Online Resource SI 2), ALDH-SF superfamily domain **(**Fig. 7) and dehydrin superfamily domain (Fig. 8) on the SlP5CS, SlSSADH and SlDHN proteins, respectively.

Motif analysis indicated that the phylogenetic relationships of the studied proteins were similar to the conserved motif distributions within the clade, with few differences. The P5CS motif distributions for the SlP5CS-1 and SlP5CS-2 proteins revealed conserved motif numbers of 1, 2, 3, 4, 5, 6, 7, 8, 9 and 10 (Fig. S1 Online Resource SI 2 and Sheet 2 Online Resource SI 1).

The SSADH motif distributions for the SlSSADH-10, SlSSADH-15, SlSSADH-9, SlSSADH-7 and SlSSADH-17 proteins revealed conserved motif numbers of 1, 2, 3, 4, 5, 6, 7, 8, 9 and 10. The motif distributions for the SlSSADH-8, SlSSADH-13, SlSSADH-16, SlSSADH-18 proteins revealed conserved motif numbers of 1, 2, 3, 4, 5, 6, 7, 8, and 9 **(**Fig. 7 and Sheet 3 Online Resource SI 1).

The DHN motif distributions for the SlDHN-1, SlDHN-4, SlDHN-13, SlDHN-12, SlDHN-8, SlDHN-7, SlDHN-11, SlDHN-14, SlDHN-15, and SlDHN-10 proteins presented conserved motif numbers of 1 and 2 **(**Fig. 8 and Sheet 4 Online Resource SI 1).

The exon‒intron structure plays a major role in plant genome evolution [52] and in maintaining genome stability [53]. The gene structure results revealed that all of the *P5CS* (Fig. S1 Online Resource SI 2), *SSADH* **(**Fig. 7) and 8 *DHN* **(**Fig. 8) genes had introns while SlDHN-13, SlDHN-12, SlDHN-8, SlDHN-7, SlDHN-11, SlDHN-14, SlDHN-10, SlDHN-15 not have introns.

**Fig. 9:**
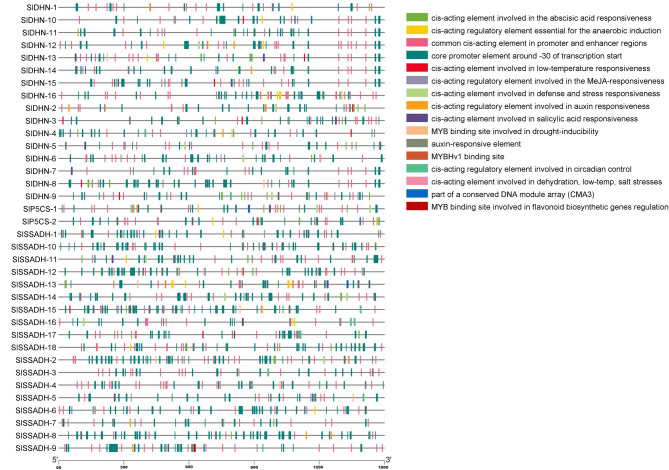
Cis-acting elements in the promoter regions (1500 bp upstream of the start codon) of the *P5CS*,* SSADH* and *DHN* genes

The studied gene sequences (1500 bp upstream of the start codon) (Table S3 Online Resource SI 1) were selected for cis-element analysis using the PlantCARE web tool to identify their biological functions (stress response, growth and development). The promoter regions of the studied genes contain a large number of plant hormone response elements. All studied genes contain cis-acting elements involved in defense and stress responsiveness, cis-acting elements involved in abscisic acid responsiveness, cis-acting elements involved in methyl jasmonate (MeJA) responsiveness, salicylic acid, cis-acting elements involved in dehydration and the MYB binding site (MBS) involved in drought inducibility, which are involved in the drought response (Fig. 9).

### Subcellular localization, nuclear localization signal, transmembrane helices, and phosphorylation sites of the *P5CS*, *SSADH* and *DHN* proteins in *S. lycopersicum*

Subcellular localization analysis revealed that the P5CS proteins were predicted to be expressed in chloroplasts and the endoplasmic reticulum (ER).

SSADH proteins were predicted to be expressed in the peroxisomes, mitochondria, chloroplasts, the nucleus, and extracellular regions. DHN proteins were predicted to be expressed in the nucleus. A heatmap was constructed to predict the subcellular localization of the P5CS, SSADH and DHN proteins, as shown in Fig. S2 Online Resource SI 2 and Table S4 Online Resource SI 1.

One nuclear localization signal (NLS) was predicted for the SlSSADH-16 protein, 7 NLSs were predicted for the SlDHN protein, and no NLSs were predicted for the P5CS protein (Fig. S3, S10 Online Resource SI 2 and Table S5 Online Resource SI 1).

The TMHMM results predicted the transmembrane helices in the SlSSADH-6, SlSSADH-7 and SlSSADH-18 proteins, whereas no transmembrane helices were predicted for the SlP5CS and SlDHN proteins (Fig. S11:S13 Online Resource SI 2 and Table S6 Online Resource SI 1).

The phosphorylation site prediction results for the studied proteins for kinases are shown in Fig. S14: Fig. S16 Online Resource SI 2 and Table S7 Online Resource SI 1.

### Three-dimensional (3-D) structure prediction and functional interaction network analysis of the *P5CS*, *SSADH* and *DHN* proteins in *S. lycopersicum*

**Fig. 10:**
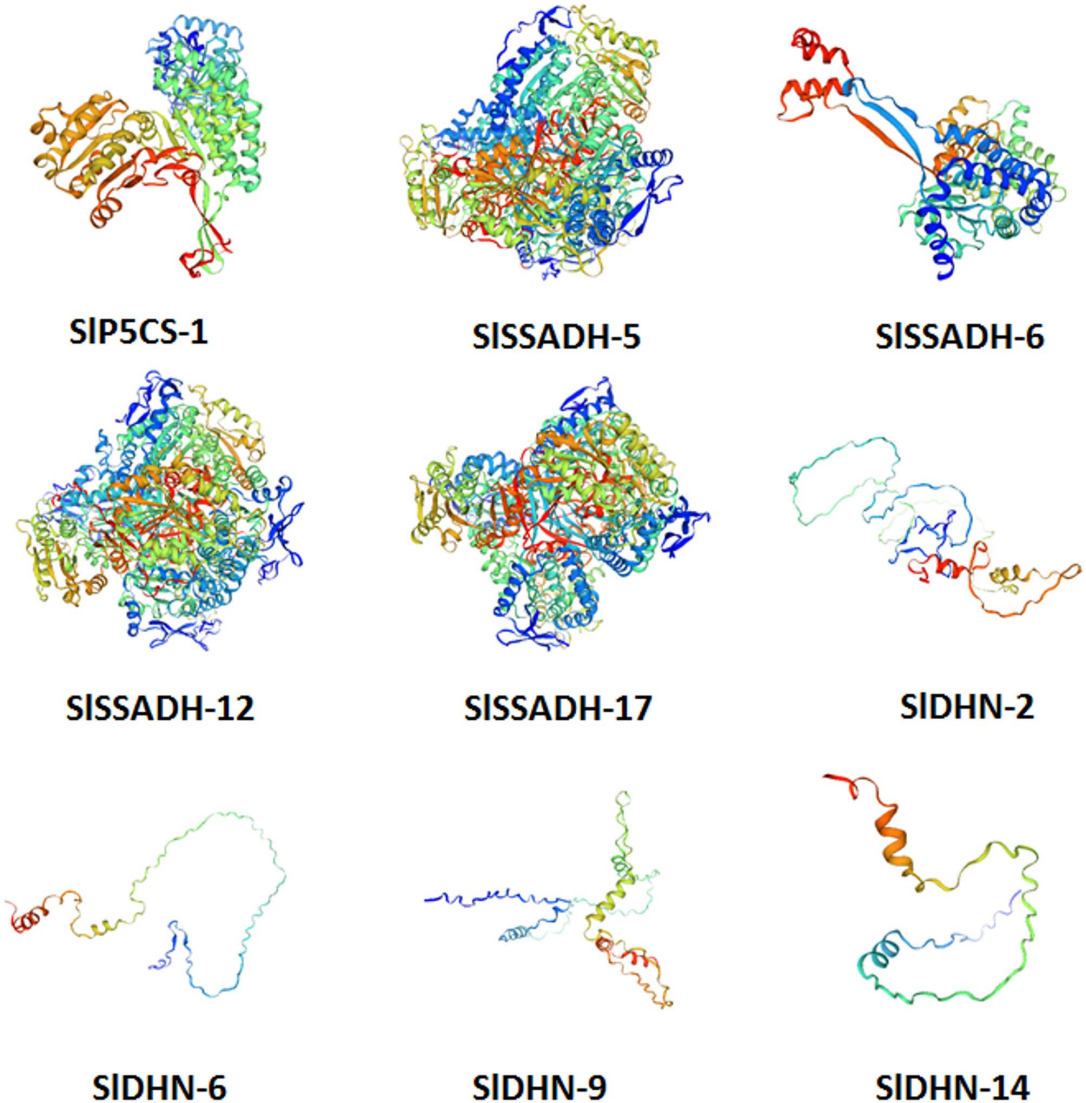
Structural analysis of the SlP5CS-1, SlSSADH-5, SlSSADH-6, SlSSADH-12, SlSSADH-17, SlDHN-2, SlSSADH-6, SlDHN-9 and SlDHN-14 proteins

To investigate the putative functions of the studied proteins, three-dimensional (3D) protein models were generated using Swiss-Model for structural prediction (Fig. 10 and S17:S25 Online Resource SI 2), while I-TASSER was employed to identify structural analogs from the Protein Data Bank (PDB) based on crystallographic templates. Model confidence was evaluated using C-score, TM-score, and RMSD values.

The closest structural analogs for SlP5CS-1 and SlP5CS-2 was with 7f5uA, suggesting strong similarity to known crystallographic models. The closest structural analogs for the SSADH isoforms was with 7na0A, 6k0zA, 5ur2A, and 1o01C. The closest structural analogs for the SlDHN proteins was with 4nl6A, 1ubmL, 4ke2A, and 2eh3A2 (Table 2). Since proteins with similar 3D structures often share comparable biological functions, these models support further structural and functional characterization of P5CS and SSADH proteins in drought tolerance, whereas DHN models require further refinement or experimental validation.

To further explore the potential functions of studied proteins in protein‒protein interactions (PPIs) with other proteins, a protein–protein interaction (PPI) network was constructed using the Search Tool for the Retrieval of Interacting Proteins (STRING) database, as shown in Fig. S26, S43 Online Resource SI 2.

Both SlP5CS-1 and SlP5CS-2 were predicted to interact with several enzymes related to nitrogen and proline metabolism, including glutamine synthetases, glutamate dehydrogenases, and ornithine aminotransferases (Fig. S26 Online Resource SI 2). Various SlSSADH isoforms showed distinct but overlapping interaction patterns with amino oxidases, aldehyde dehydrogenases, protein kinases, and other stress-related proteins (Fig. S27, S35 Online Resource SI 2). Likewise, SlDHN was predicted to interact with multiple lipid-transfer proteins, and actin-related proteins (Fig. S36, S43 Online Resource SI 2).

These predicted interactions suggest that P5CS, SSADH, and DHN proteins may be involved in complex stress-responsive networks, potentially coordinating metabolic and signaling pathways to enhance plant tolerance to abiotic stresses such as drought and salinity.

**Table 2:** Structural modeling results of *S. lycopersicum* P5CS, SSADH, and DHN proteins predicted by I-TASSER. The table includes C-score, TM-score, and RMSD values, as well as the best structural analogs identified from the protein data bank (PDB). Higher C-scores and TM-scores indicate greater confidence in the predicted structure, while lower RMSD values reflect better structural alignment with known proteins

Protein	C-Score	TM-Score	RMSD (Å)	Best Identified Structural Analogs in PDB				
				PDB Hit	TM-Score^a^	RMSD^a^	IDEN^a^	Cov
SlP5CS-1	1.19	0.88 ± 0.07	5.5 ± 3.5	7f5uA	0.941	0.53	0.473	0.944
SlSSADH-5	−0.61	0.64 ± 0.13	9.0 ± 4.6	7na0A	0.848	2.58	0.278	0.908
SlSSADH-6	0.05	0.72 ± 0.11	7.1 ± 4.2	6k0zA	0.933	0.51	0.395	0.937
SlSSADH-12	−0.67	0.63 ± 0.14	9.2 ± 4.6	5ur2A	0.838	2.71	0.189	0.898
SlSSADH-17	0.71	0.81 ± 0.09	5.8 ± 3.6	1o01C	0.979	0.96	0.512	0.988
SlDHN-2	−3.29	0.35 ± 0.12	13.7 ± 4.0	4nl6A	0.778	3.61	0.056	0.996
SlDHN-6	−3.78	0.30 ± 0.10	13.2 ± 4.1	1ubmL	0.488	5.10	0.031	0.908
SlDHN-9	−4.93	0.21 ± 0.06	17.7 ± 2.5	4ke2A	0.507	4.75	0.043	0.762
SlDHN-14	−3.69	0.31 ± 0.10	11.6 ± 4.5	2eh3A2	0.616	3.00	0.093	0.922

### Prediction of miRNAs targeting the P5CS, SSADH and DHN proteins in *S. lycopersicum*

Using a cut-off threshold of 5 for the search parameters, 13 microRNAs were predicted to target the proteins in 2 *P5CS*, 134 microRNAs were predicted to target the genes in 18 *SSADH*, and 26 microRNAs were predicted to target the genes in 16 *DHN*. The use of an expectation score of less than 3.5, can be considered more reliable. This resulted in 3 microRNAs being predicted to target the genes in 2 SSADH, 14 microRNA to target the proteins in 13 DHN, and no microRNA to target the P5CS genes. The microRNA targeting relationships for the *P5CS*, *SSADH*, and *DHN* genes are presented in Table S8 of Online Resource SI 1.

The results from the prediction of RNA secondary structures with pseudoknots for the *P5CS* (SlP5CS-1), *SSADH* (SlSSADH-5, SlSSADH-6, SlSSADH-12, and SlSSADH-17) and *DHN* (SlDHN-2, SlDHN-6, SlDHN-9, and SlDHN-14) proteins are shown in Fig. S44: S52 Online Resource SI 2.

### Gene Ontology enrichment and functional relationship analysis of the *P5CS*,*SSADH* and *DHN* genes in *S. lycopersicum*

To further determine the functions of the studied genes, we performed enrichment analysis and gene ontology (GO) analysis based on biological processes and molecular functions. GO terms help us to understand the function of genes at the molecular level (Fig. S53:S55 Online Resource SI 2). GO terms for the studied genes confirmed the functional role of studied genes as stress responsive genes (Fig. S56:S58 Online Resource SI 2).

In this study, qRT‒PCR analysis revealed that the studied proteins were expressed in leaves, and drought increased the expression levels of *P5CS* and *DHN* by 2.39 and 1.23, respectively, whereas the expression level of *SSADH* decreased to 0.73 (Sheet 1 Online Resource SI 1). Domain structure, promoter and gene ontology enrichment analyses confirmed the functional role of the studied proteins in drought responses.

## Discussion

Genome-wide identification and characterization of gene families play a fundamental role in unraveling the molecular mechanisms underlying plant responses to abiotic stresses. In tomato (*S*. *lycopersicum*), such comprehensive analyses are essential to elucidate the specific functions of key gene families involved in drought tolerance, enabling targeted genetic improvement and stress management strategies [54–56]. In this context, our study represents the first genome-wide identification of *P5CS*, *SSADH*, and *DHN* genes in *S*. *lycopersicum* under drought stress, revealing 2 *P5CS*, 18 *SSADH*, and 16 *DHN* genes.

Our phylogenetic classification results revealed that the 18 SSADH proteins identified in *S*. *lycopersicum* could be divided into three distinct clades. Similar classifications have been reported in other plant species, including *A*. *thaliana*, where *SSADH* genes show distinct evolutionary groupings related to functional divergence [57]. Comparable results were also observed in other *Solanaceae* members such as *S*. *tuberosum* [58] and *Capsicum annuum* [59], suggesting a conserved phylogenetic pattern across closely related species. These findings support the hypothesis that *SSADH* gene diversification is functionally relevant and maintained through plant evolution. Likewise, phylogenetic analysis of the 16 SlDHN proteins revealed that the identified in *S. lycopersicum* are grouped into three distinct clades, suggesting possible functional divergence among the subfamilies [8]. This classification is consistent with previous studies that reported similar phylogenetic clustering patterns of dehydrin proteins in various plant species [60].

Chromosomal distribution analysis revealed that *P5CS* genes were located on chromosomes 6 and 8. *SSADH* genes were found on chromosomes 1, 2, 3, 5, 6, 7, 8, 9, and 12, while *DHN* genes were distributed across chromosomes 1, 2, 3, 4, 5, 6, 7, 8, 9, 10, and 12. The chromosomal distribution of *SSADH* genes suggests potential gene duplication events and segmental rearrangements that may have contributed to their evolutionary expansion. Similar patterns of dispersed chromosomal localization have been reported in *S*. *tuberosum*, where gene family members related to drought tolerance are often spread across multiple chromosomes, reflecting their diversification and functional specialization [58]. Chromosomal mapping of *SlDHN* genes in *S. lycopersicum* revealed an uneven distribution: two genes on chromosome 1, five on chromosome 2, and one gene each on chromosomes 3 through 10 and 12. This uneven distribution suggests that gene duplication events might have contributed to the expansion of the *DHN* gene family, particularly on chromosome 2. Similar patterns of scattered distribution have also been reported in other plant species [60, 61]. Although only two *P5CS* members were identified, their presence on separate chromosomes (6 and 8) may indicate an ancient duplication event or functional divergence, as observed in other *Solanaceae* species.

In this study, the Ka/Ks ratios indicated that the *P5CS*, *SSADH*, and *DHN* genes were primarily subjected to purifying selection, suggesting that these genes have been evolutionarily conserved due to strong functional constraints. Such selection pressure implies that mutations in these genes are likely deleterious, and therefore eliminated through evolutionary processes to preserve their essential roles in stress responses. This finding is consistent with previous reports in *A*. *thaliana* and other crops, where key drought-responsive gene families—particularly those involved in osmoprotection and ROS detoxification—exhibited low Ka/Ks ratios, reflecting evolutionary conservation under environmental pressure [62, 63]. These evolutionary constraints reinforce the idea that studied genes have retained essential biological roles—namely in proline biosynthesis, GABA metabolism, and dehydration tolerance—throughout plant evolution.

In this study, our gene duplication analysis revealed distinct evolutionary patterns among the studied gene families. The single duplication event of the *P5CS* gene pair was estimated to have occurred approximately 40.030 million years ago (Mya), while the *DHN* paralogous pair showed an even earlier duplication time of around 102.128 Mya and have exhibited a broader range of duplication events, with estimated divergence times spanning from approximately 1.645 to 102.128 Mya indicating multiple independent duplication events over a long evolutionary timeframe. The duplication time of the *SSADH* paralogous gene pair ranged from approximately 31.495 to 45.966 Mya. Previous studies showed that the diversification of *SSADH* genes may be linked to subfunctionalization or neofunctionalization processes, allowing functional specialization in response to varying stress signals and tissue-specific demands—a pattern also observed in large stress-responsive gene families in other plant species [64, 65].

Domain architecture analysis revealed that the P5CS, SSADH, and DHN proteins contain the AAK superfamily domain, the ALDH-SF domain, and the dehydrin domain, respectively. These conserved domains are known to be closely associated with plant responses to abiotic stress, particularly drought, salinity, and temperature extremes. The AAK (Amino Acid Kinase) domain in P5CS plays a central role in proline biosynthesis, which is a key osmoprotectant during water deficit conditions [66]. The ALDH-SF (Aldehyde Dehydrogenase Superfamily) domain found in SSADH proteins contributes to cellular detoxification by converting reactive aldehydes into less harmful compounds, thereby enhancing stress tolerance [67]. Likewise, the dehydrin domain is a hallmark of *LEA* (*Late Embryogenesis Abundant*) proteins that accumulate under dehydration and protect cellular structures from damage [8]. The presence of these domains across the three gene families reinforces their functional relevance in stress adaptation. Moreover, the conservation of these domains across plant species suggests evolutionary pressure to retain their structure and function under adverse environmental conditions.

The conservation of motif patterns and gene structures among closely related gene members implies strong functional constraint within each gene family. Genes clustering together in the phylogenetic tree—such as SlP5CS-1/2, SlSSADH-10/15/5/9/7/17, and SlDHN-1/4/13/12/8/7/11/14/15/10—shared highly similar motif compositions, supporting their evolutionary relatedness and possibly redundant or complementary functional roles. These findings are consistent with previous reports indicating that motif and exon-intron conservation reflect selective pressure to maintain key biological functions during plant adaptation to stress [68, 69].

Cis-acting regulatory elements are crucial in mediating plant responses to abiotic stresses. These elements, located in the promoter regions of stress-responsive genes, regulate transcriptional activity in response to environmental cues. Fan et al. [70] reported that conserved cis-elements in promoter regions are functionally associated with multi-stress tolerance mechanisms. Our analysis revealed that the promoter regions of studied genes harbor multiple cis-acting regulatory elements associated with abiotic stress responses. These include elements involved in defense and general stress responsiveness (e.g., TC-rich repeats), abscisic acid (ABRE), methyl jasmonate (MeJA; CGTCA-motif and TGACG-motif), salicylic acid (TCA-element), dehydration (e.g., DRE), and MYB binding sites (MBS) linked to drought inducibility. The presence of these elements suggests transcriptional regulation of these genes under diverse stress conditions.

The predicted subcellular localization patterns of the studied proteins highlight their potential functional specialization in different cellular compartments during drought stress responses. The localization of P5CS proteins in chloroplasts and the endoplasmic reticulum (ER) suggests their involvement in both osmoprotectant biosynthesis and protein processing, consistent with previous findings showing chloroplast-localized P5CS enzymes catalyze proline biosynthesis under osmotic stress [6]. The broader distribution of SSADH proteins—including peroxisomes, mitochondria, chloroplasts, the nucleus, and extracellular regions—may reflect its role in reactive oxygen species (ROS) detoxification and GABA metabolism across multiple organelles, as previously suggested in Arabidopsis and other plants [7, 71]. Meanwhile, the nuclear localization of DHN proteins supports their protective function in stabilizing chromatin and nuclear macromolecules under dehydration, which aligns with their classification as LEA group 2 proteins [72].

Synteny analysis showed clear evolutionary partitioning of the studied gene families between eudicots and monocots. 1 orthologous *P5CS* gene, 20 *SSADH* genes, and 5 *DHN* genes in *S*. *lycopersicum* aligned collinearly with those of the closely related Solanaceae species *S*. *tuberosum*, reflecting strong conservation within this family [73, 74]. A smaller but still significant set of orthologous pairs—15 for *SSADH* and 5 for *DHN*—were identified as collinear orthologs in the more distantly related eudicot *A. thaliana*, indicating that core members of these stress-response families were preserved after the divergence of rosids and asterids (~ 120 Mya) [75]. In contrast, no collinear orthologs were detected with the monocot *O*. *sativa*, supporting the view that most lineage-specific expansions or contractions of these families occurred after the monocot–dicot split (~ 150–170 Mya) and may have been reshaped further by independent whole-genome duplication and fractionation events in dicots and grasses [76]. The dense retention of *SSADH* paralogs within *Solanaceae* versus their partial loss in *A*. *thaliana* and complete absence of recognizable orthologs in rice underscores the likelihood of sub- or neofunctionalization of *SSADH* copies in eudicots, whereas *P5CS* and *DHN* appear to have remained under tighter copy-number constraint, consistent with their essential osmoprotective and dehydration-protection roles.

Our qRT‒PCR results indicated that *P5CS* and *DHN* were upregulated under drought stress, with fold changes of 2.39 and 1.23, respectively, whereas the expression level of *SSADH* decreased to 0.73. These findings are consistent with previous reports highlighting the role of *P5CS* and *DHN* genes in drought responses. For instance, Tian et al. [77] observed a significant increase in *AmP5CS* expression beginning on the third day of drought, peaking at day twelve in *Agropyron mongolicum*. Similarly, Carbonnel et al. [78] reported that *SlDHN* expression was upregulated at an early stage of drought treatment in tomatoes, while Choi et al. [79] found that ten *HvDhn* genes were induced by dehydration in barley (*Hordeum vulgare* L.). In contrast, our findings regarding *SSADH* expression align with those of Palabıyık et al. [80], who demonstrated that drought stress alone did not significantly induce *SSADH* activity. Instead, its expression was notably upregulated only upon co-treatment with flg22, a microbial elicitor. This suggests that the *SSADH* may require additional signaling cues beyond water deficit alone to become functionally active, indicating a more nuanced role in stress response pathways.

## Conclusion

This study provides the first comprehensive genome-wide characterization of the studied gene families in *S*. *lycopersicum* under drought stress. The upregulation of *P5CS* and *DHN* and the downregulation of *SSADH* highlight their coordinated roles in osmoprotection and oxidative stress responses. Phylogenetic and synteny analyses suggest strong evolutionary conservation and purifying selection shaping these gene families. Promoter and subcellular localization analyses further support their involvement in drought response mechanisms. These insights offer promising targets for the genetic enhancement of drought tolerance in tomatoes.

## Electronic supplementary material

Below is the link to the electronic supplementary material.

Supplementary Material 1

Supplementary Material 2

## Data Availability

Data is provided within the manuscript and supplementary information files.

## Abbreviations

P5CS: Pyrroline-5-carboxylate synthetase
SSADH: Succinic semialdehyde dehydrogenase
DHN: Dehydrin
Ka/Ks: Ratio of nonsynonymous/synonymous
RMSD: Root mean square deviation
