# Supplementary material for: Genome-wide identification, characterization and evolutionary analysis of the pyrroline-5-carboxylate synthetase (P5CS), succinic semialdehyde dehydrogenase (SSADH), and dehydrin (DHN) genes in Solanum lycopersicum under drought stress
Source: BMC Plant Biol. 2025 Aug 9;25:1060. doi: 10.1186/s12870-025-07057-w (PMC12335051; doi:10.1186/s12870-025-07057-w)
Supplement: Supplementary file 2 — Supplementary Material 2 [file 12870_2025_7057_MOESM2_ESM.pdf]

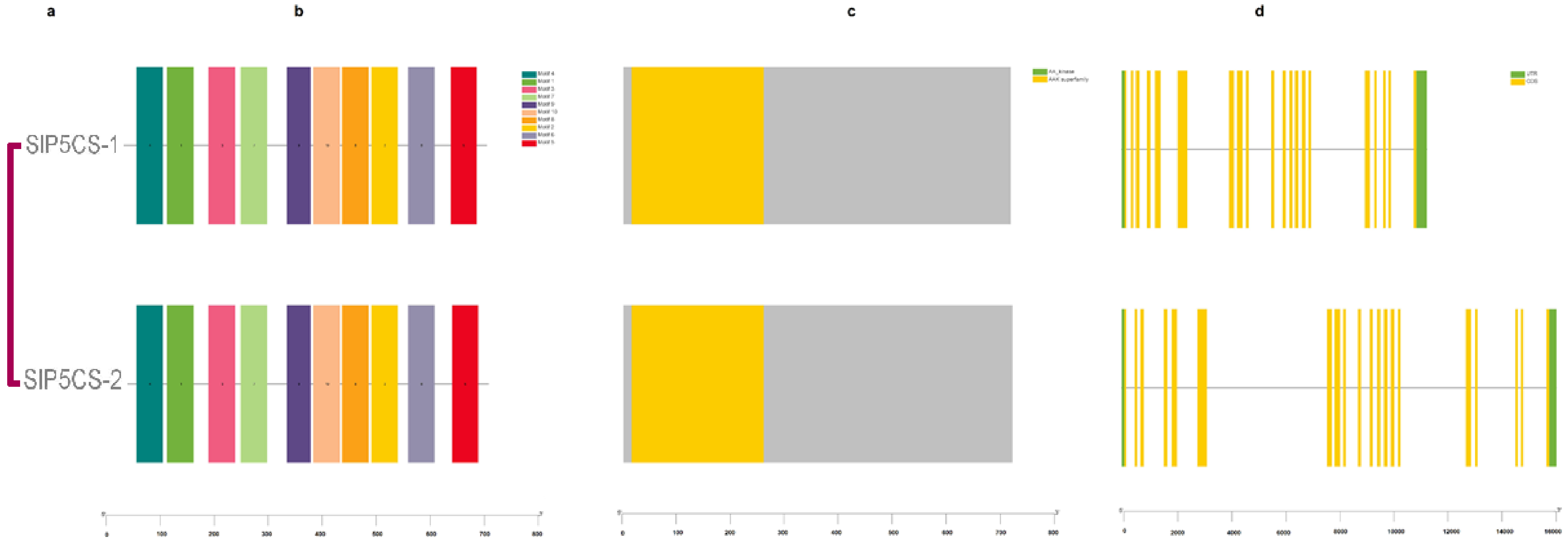

Fig. S1. P5CS proteins. (**a**) Rectangular phylogenetic tree. (**b**) Conserved motifs were predicted using MEME. (c) Protein domains. (d) Gene structure.

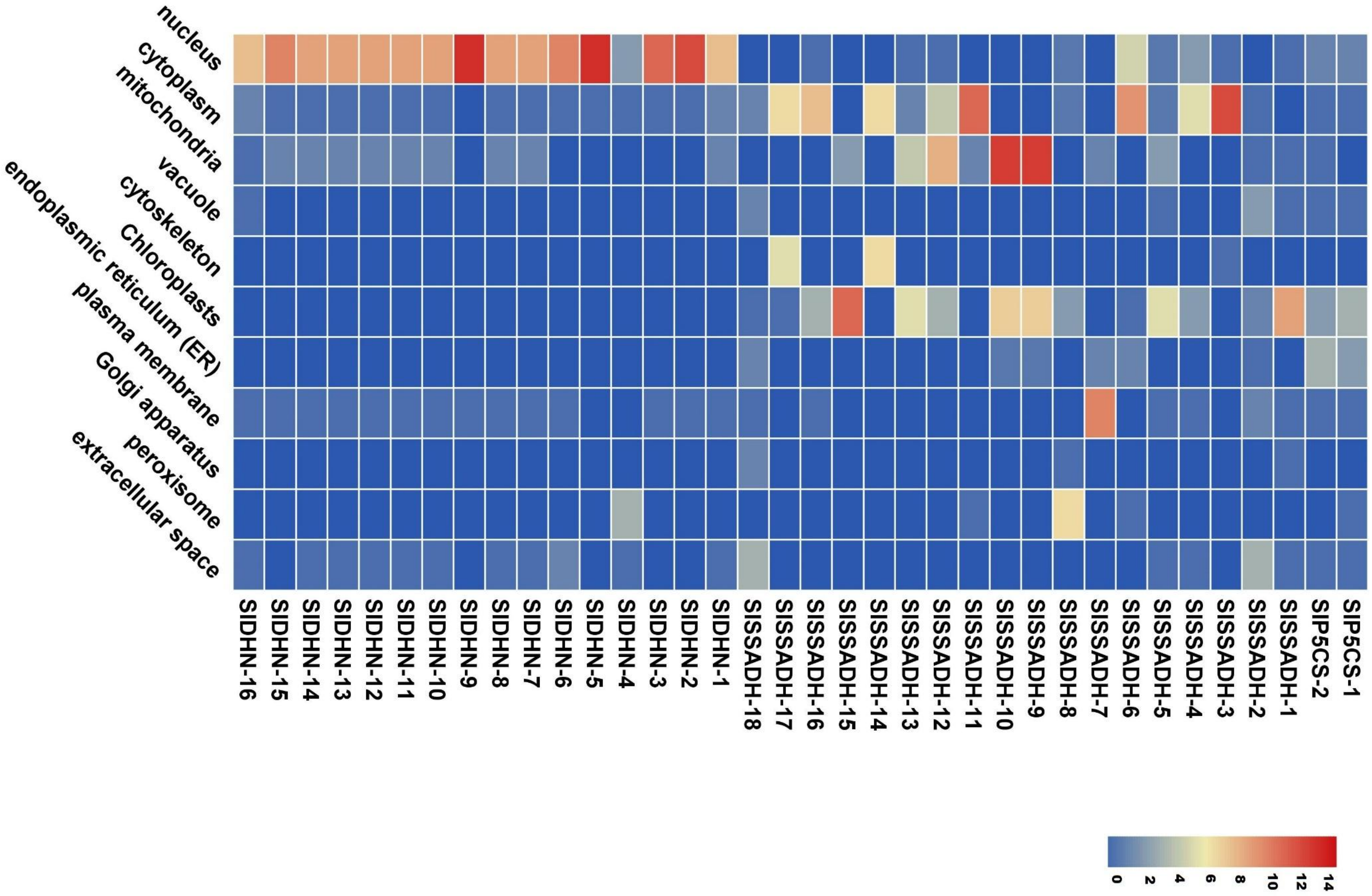

Fig. S2. Heatmap showing P5CS, SSADH and DHN proteins localization prediction in deferent organelle.

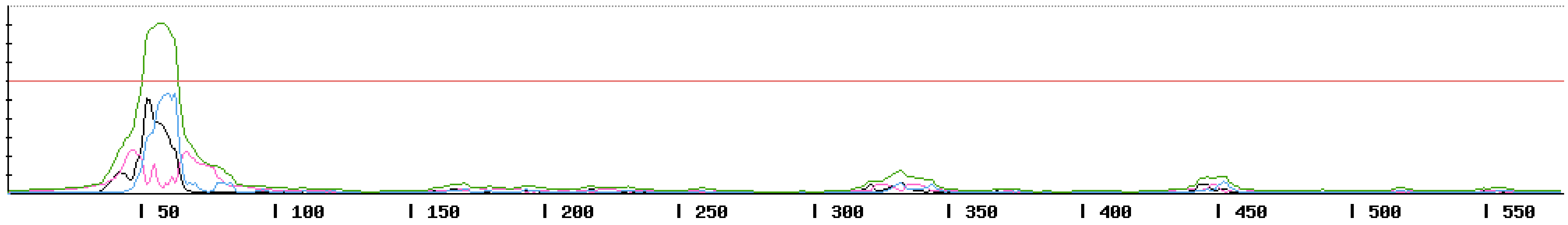

SlSSADH-16

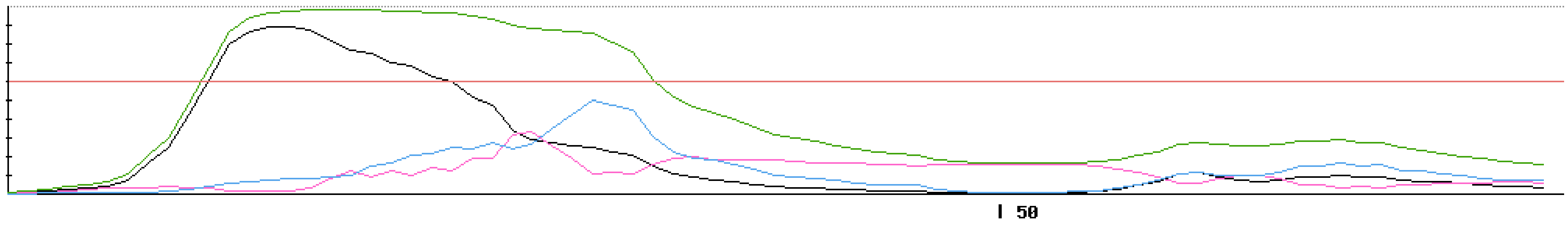

SlDHN-15

Fig. S3. Nuclear localization signal (NLS) prediction for SlSSADH-16 proteins

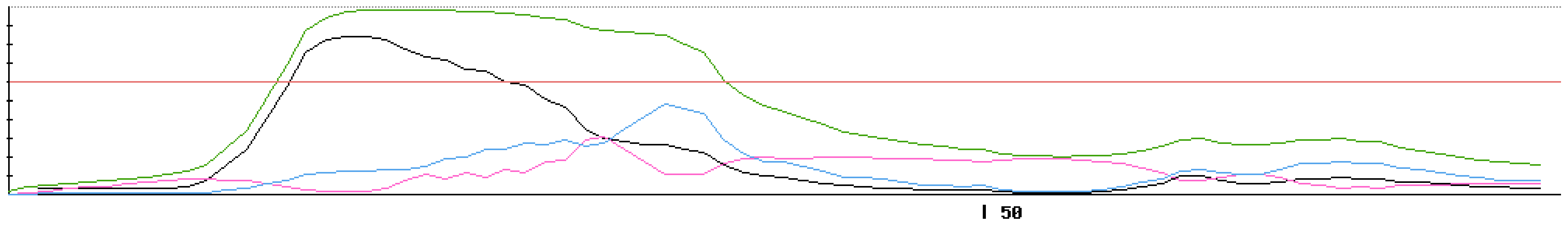

SlDHN-1

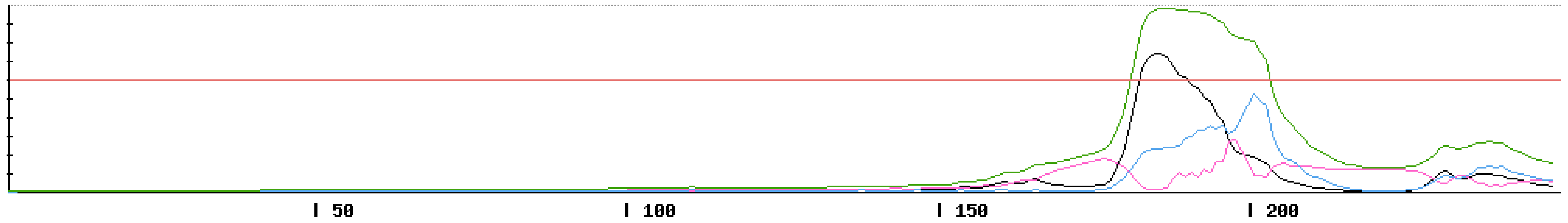

SlDHN-2

Fig. S4. Nuclear localization signal (NLS) prediction for SlDHN-6 and SlDHN-9 proteins

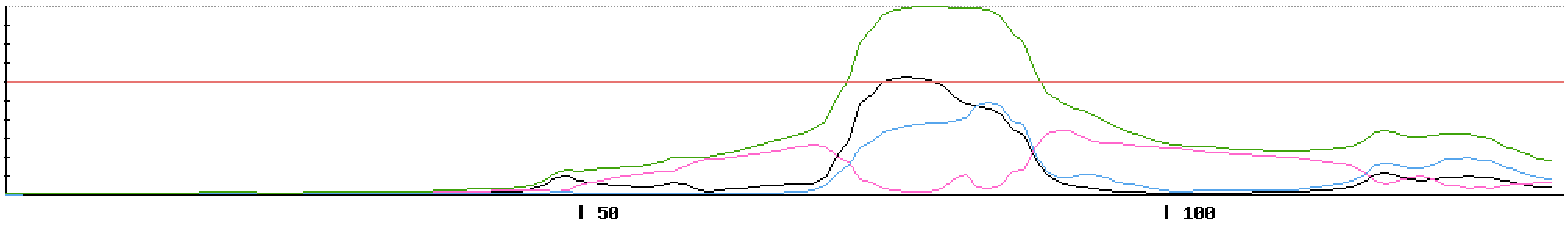

SlDHN-3

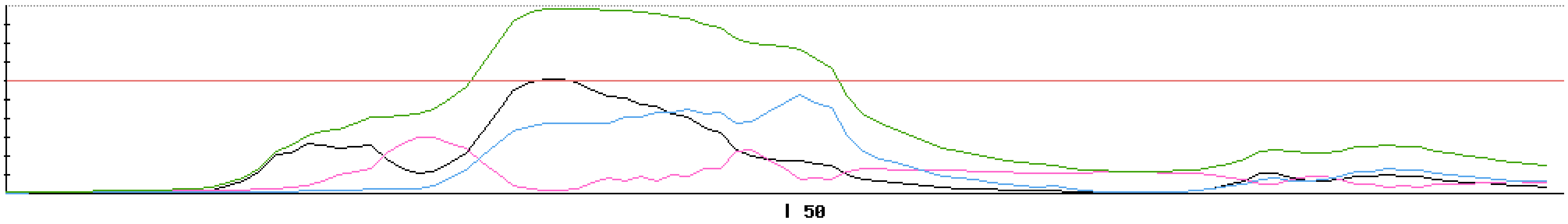

SlDHN-4

Fig. S5. Nuclear localization signal (NLS) prediction for SlDHN-6 and SlDHN-9 proteins

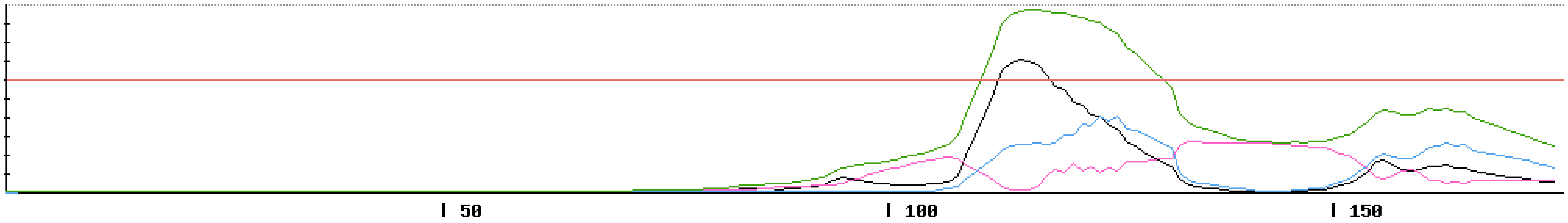

SlDHN-5

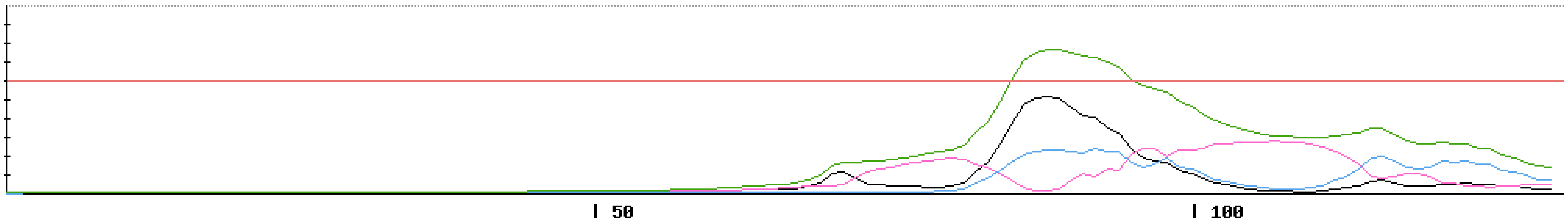

SlDHN-6

Fig. S6. Nuclear localization signal (NLS) prediction for SlDHN-6 and SlDHN-9 proteins

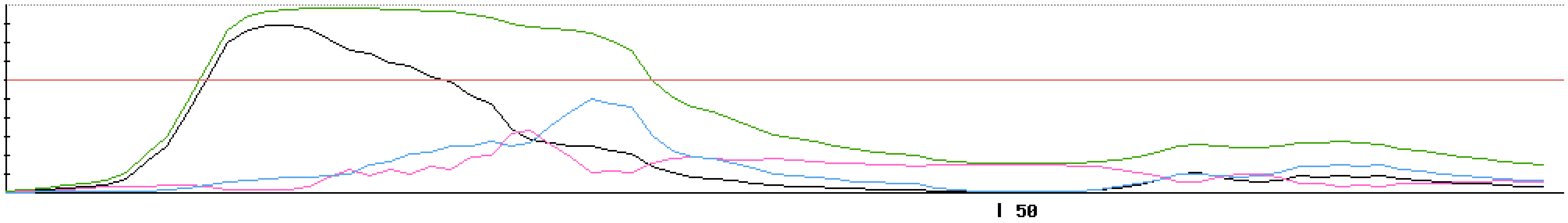

SlDHN-7

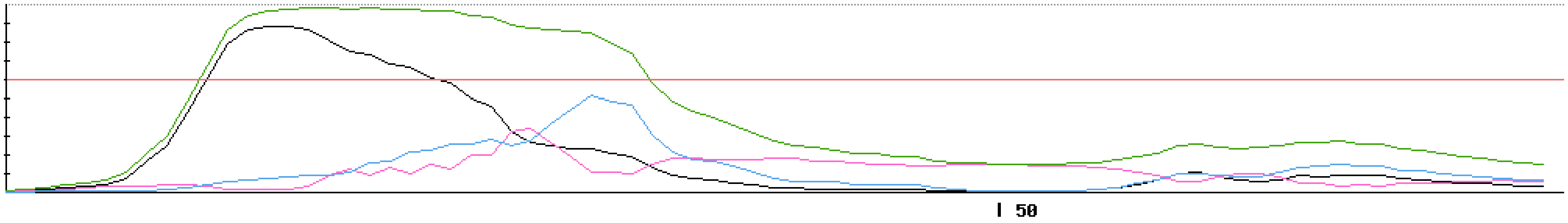

SlDHN-8

Fig. S7. Nuclear localization signal (NLS) prediction for SlDHN-6 and SlDHN-9 proteins

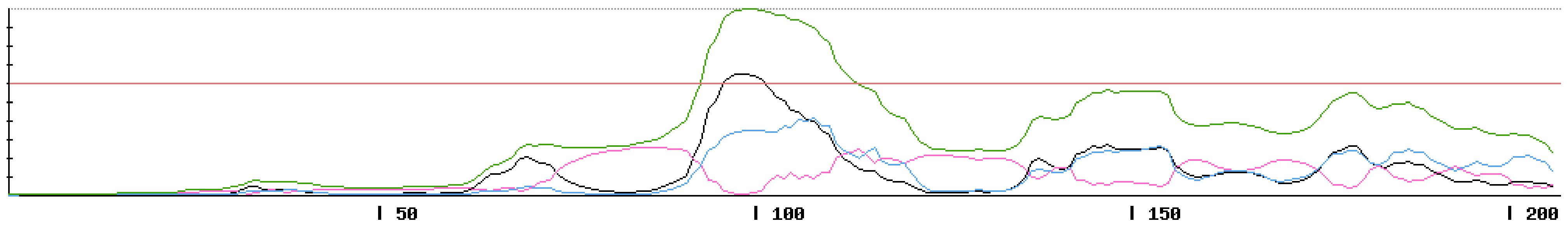

SlDHN-9

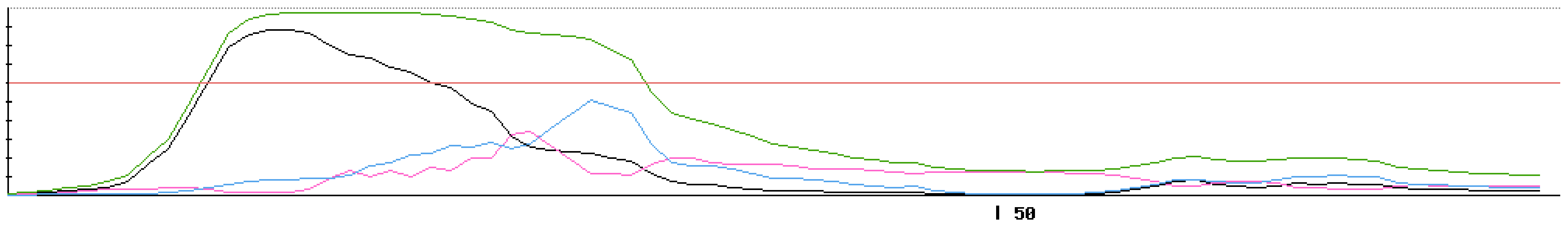

SlDHN-10

Fig. S8. Nuclear localization signal (NLS) prediction for SlDHN-6 and SlDHN-9 proteins

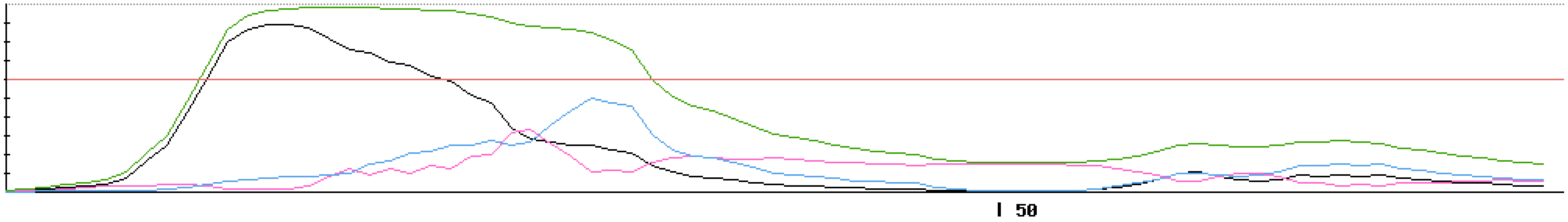

SlDHN-11

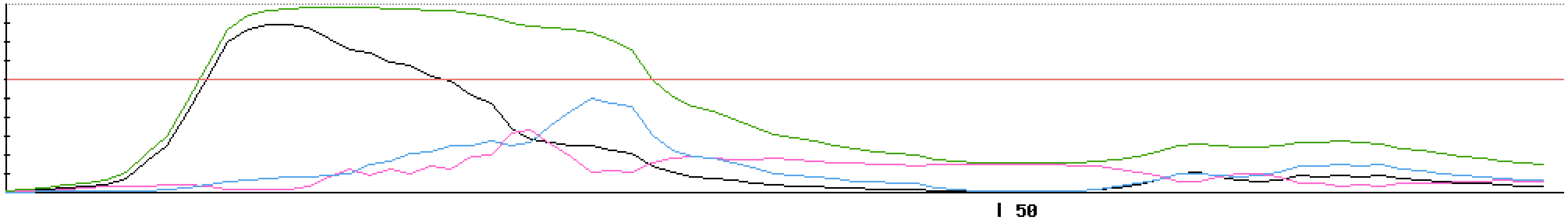

SlDHN-12

Fig. S9. Nuclear localization signal (NLS) prediction for SlDHN-6 and SlDHN-9 proteins

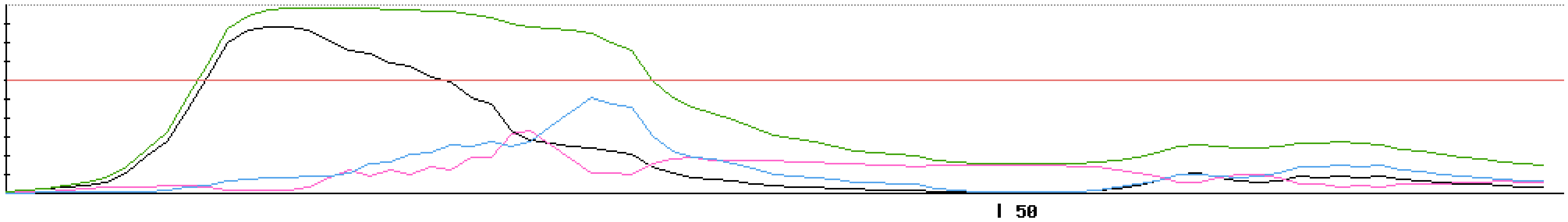

SlDHN-13

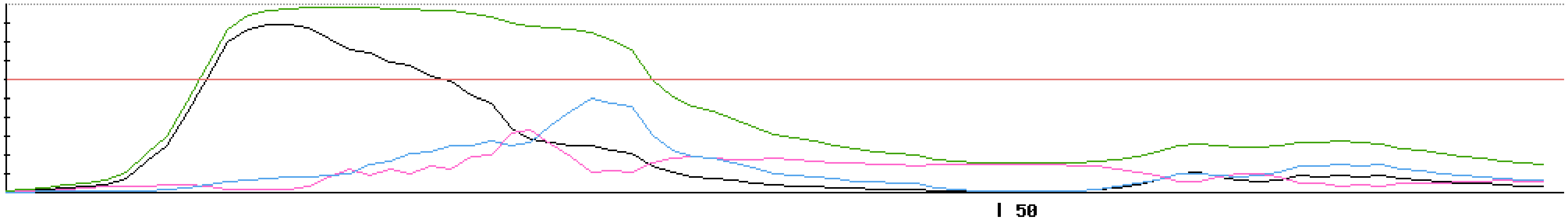

SlDHN-14

Fig. S10. Nuclear localization signal (NLS) prediction for SlDHN-6 and SlDHN-9 proteins

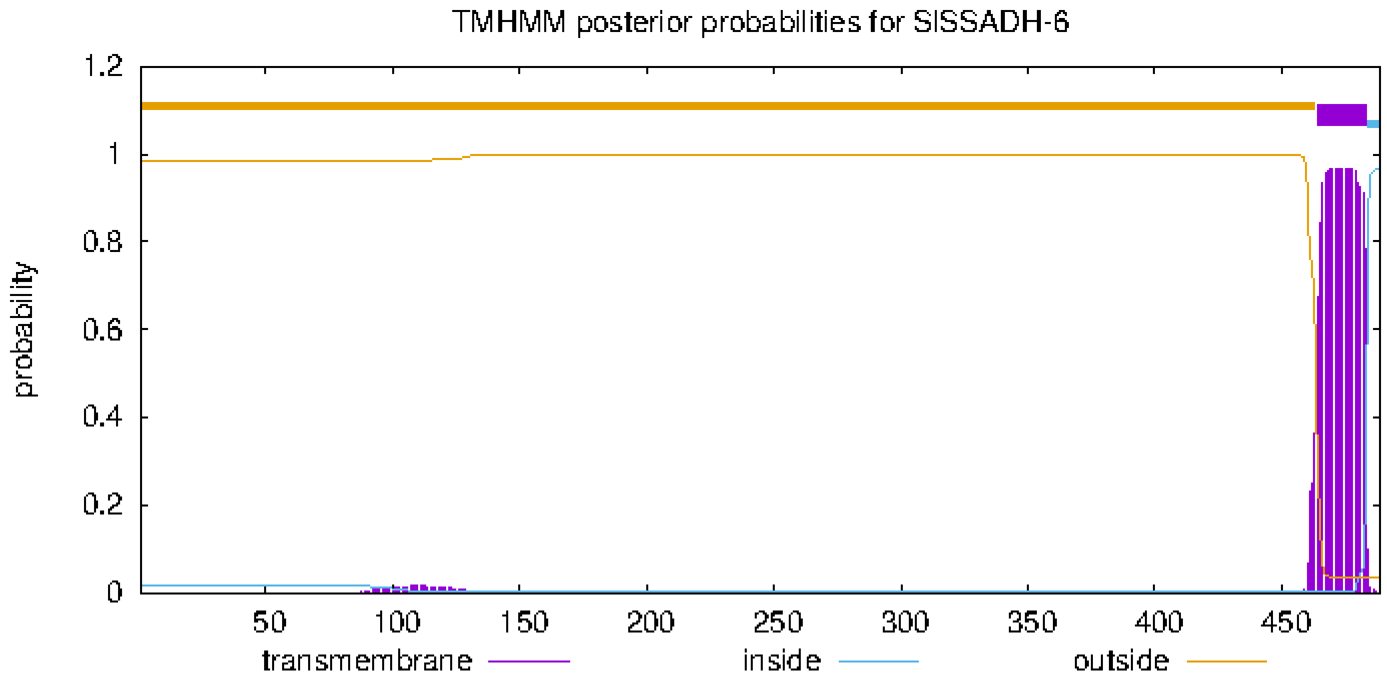

Fig. S11. The transmembrane helical in SlSSADH-6

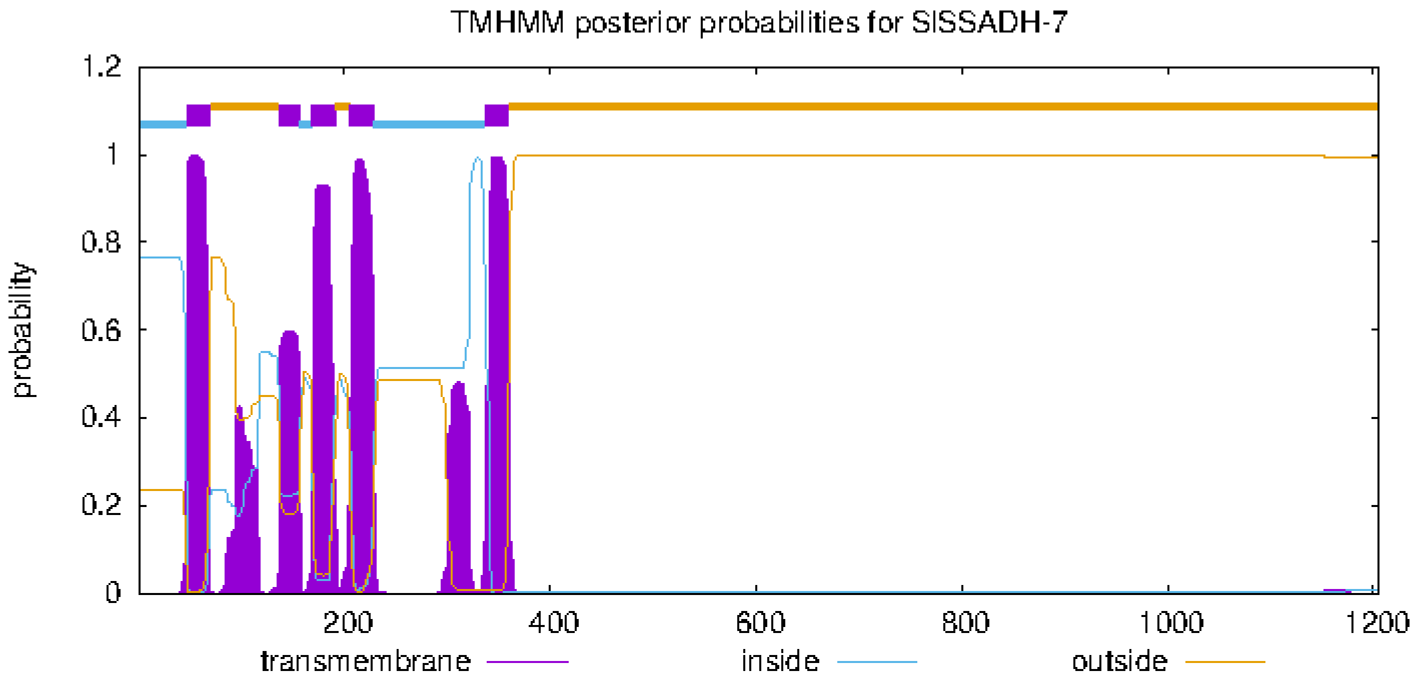

Fig. S12. The transmembrane helical in SlSSADH-7

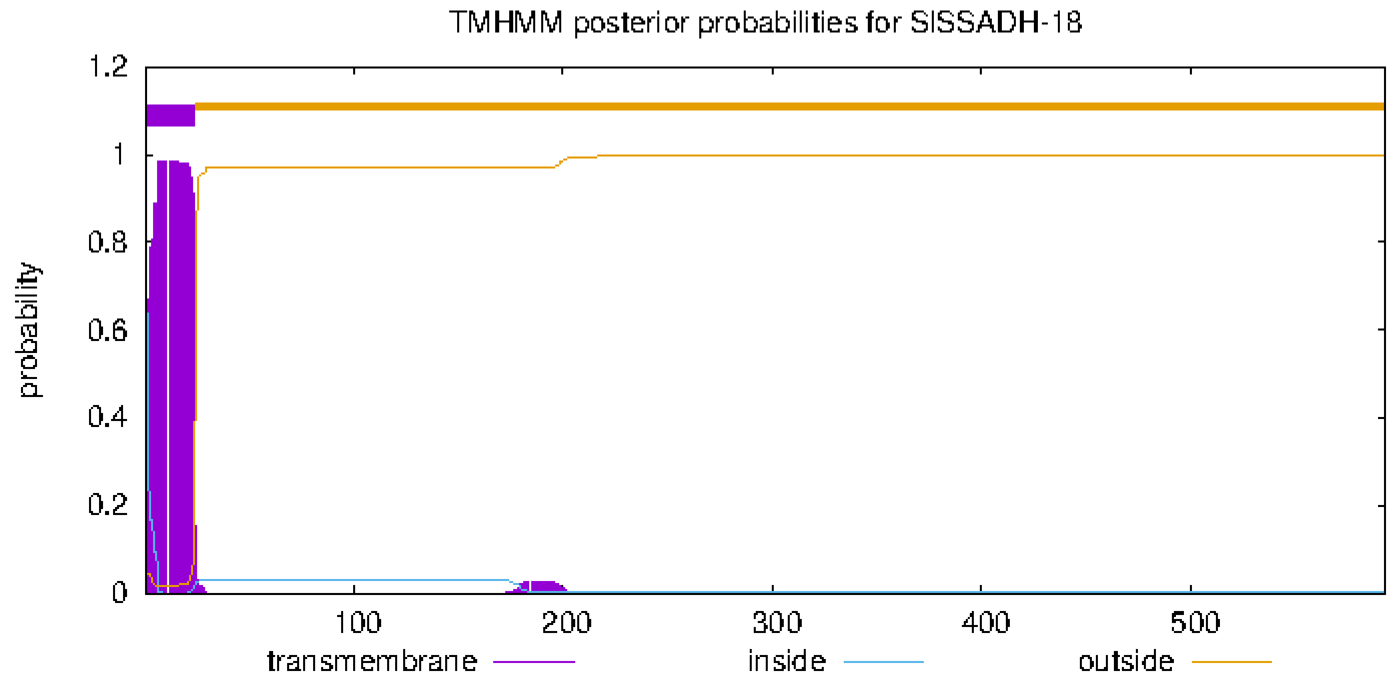

Fig. S13. The transmembrane helical in SlSSADH-18

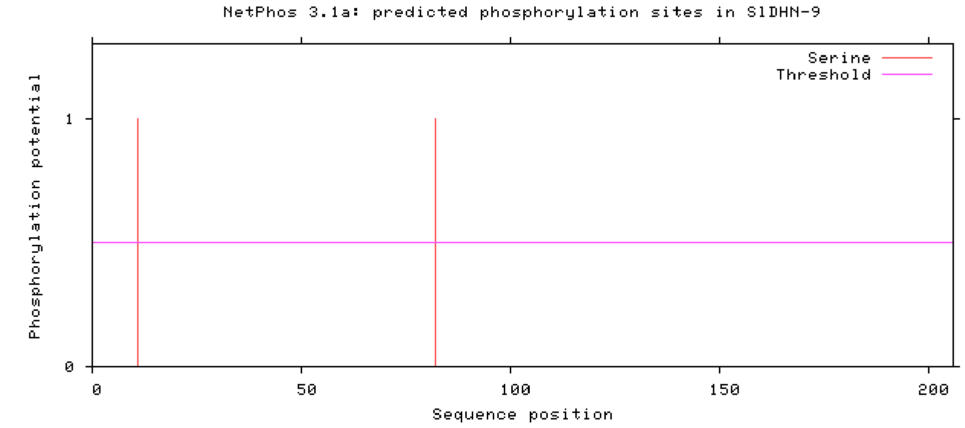

SlDHN-9

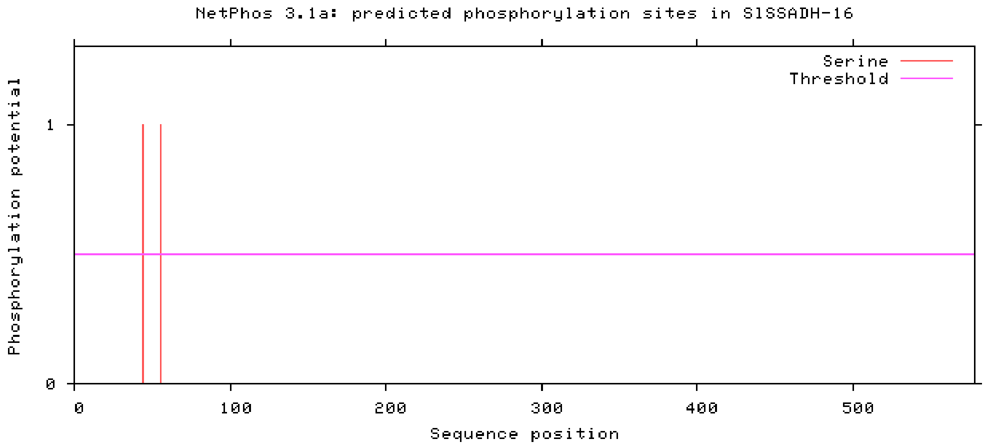

SlSSADH-16

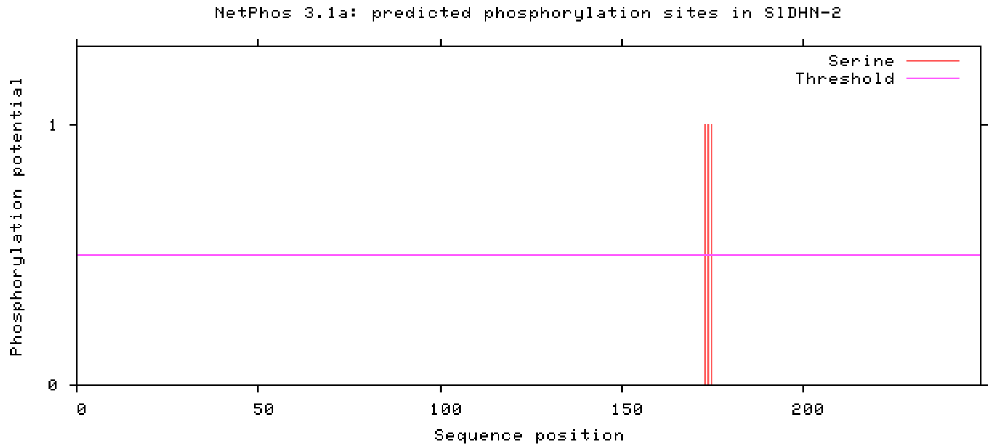

SlDHN-2

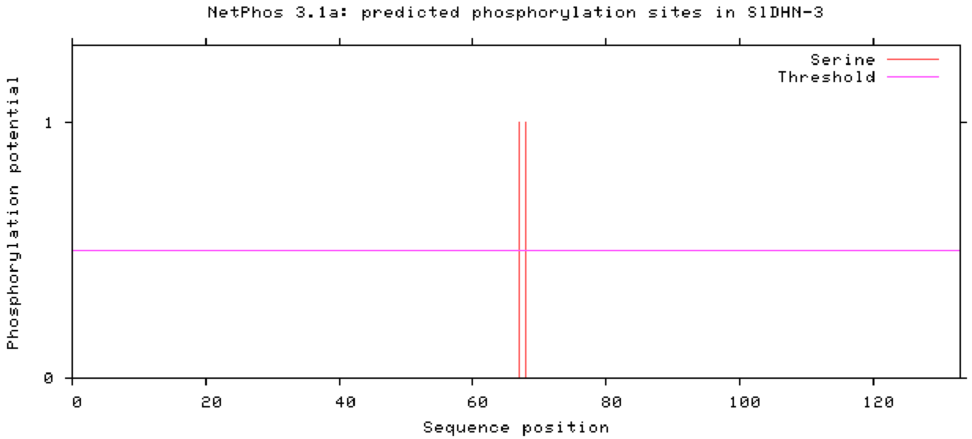

SlDHN-3

Fig. S14. Proteins phosphorylation with serine, threonine and tyrosine

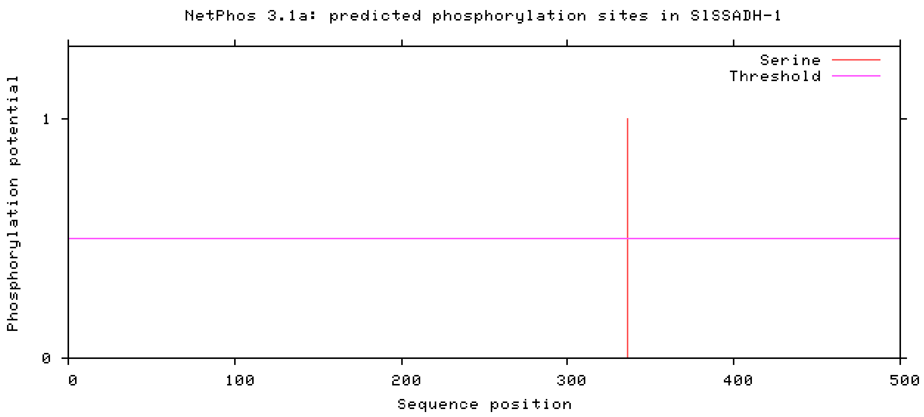

SlSSADH-1

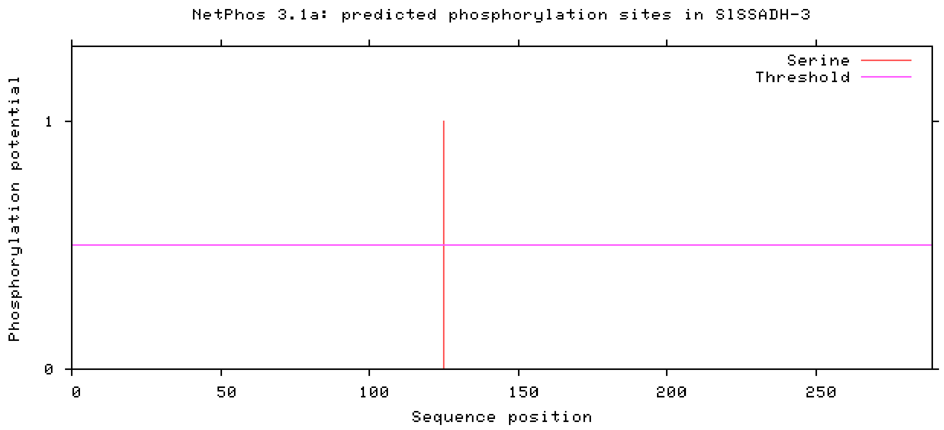

SlSSADH-3

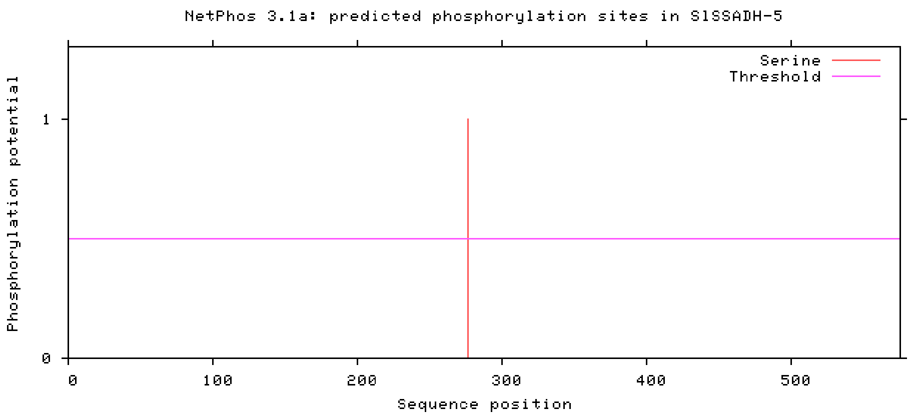

SlSSADH-5

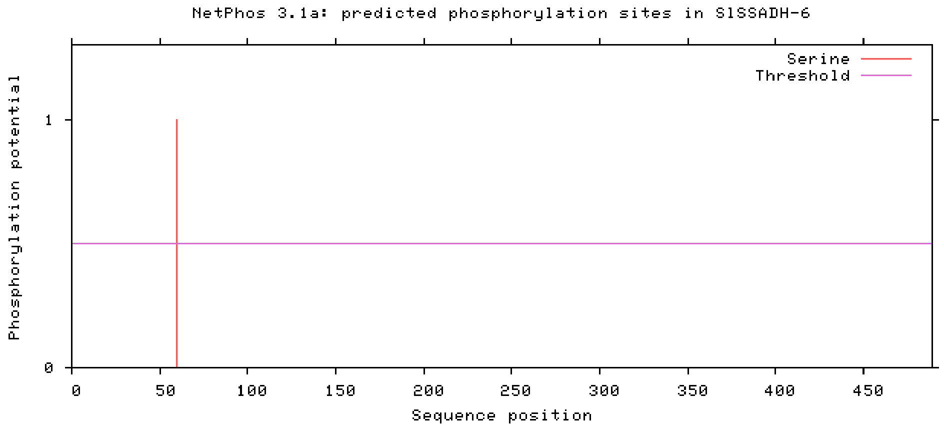

SlSSADH-6

Fig. S15. Proteins phosphorylation with serine, threonine and tyrosine

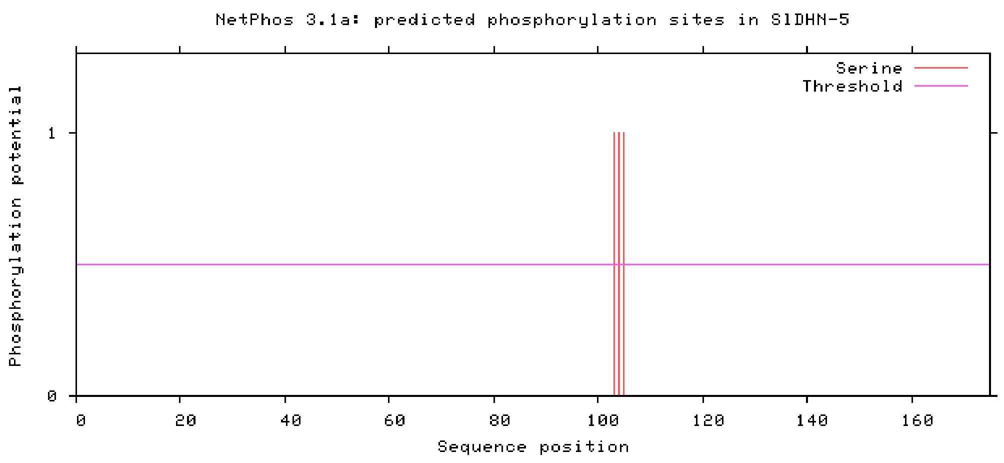

SlDHN-5

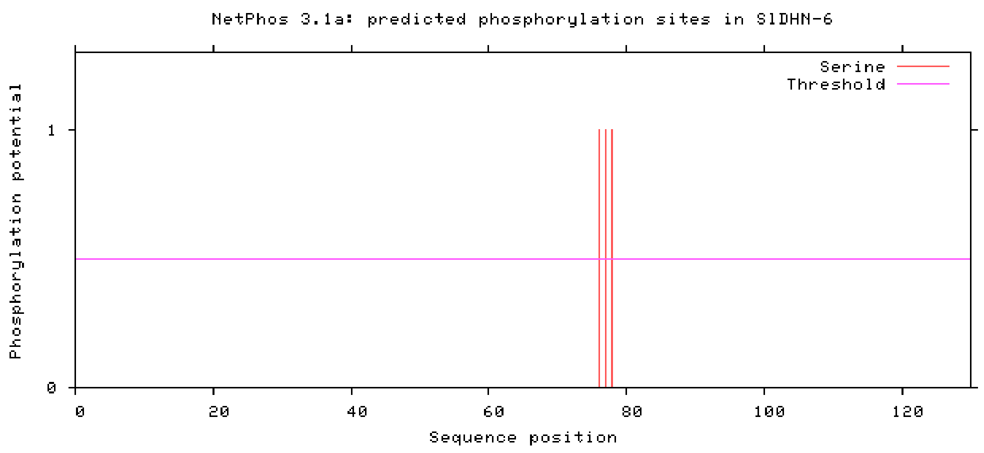

SlDHN-6

Fig. S16. Proteins phosphorylation with serine, threonine and tyrosine

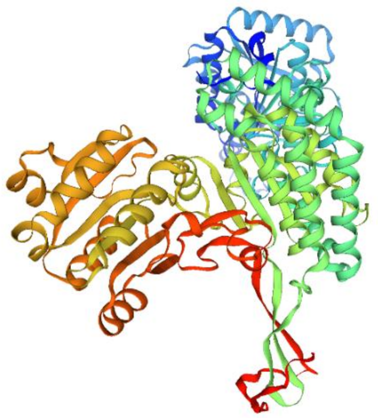

SlP5CS -1

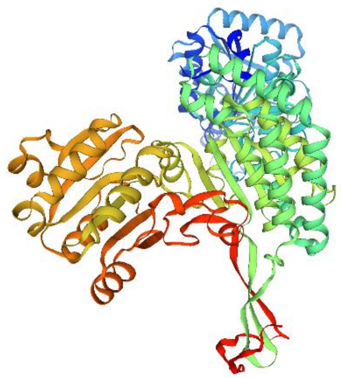

SlP5CS -2

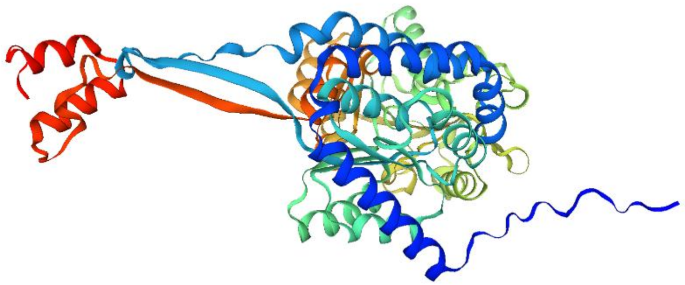

SlSSADH-1

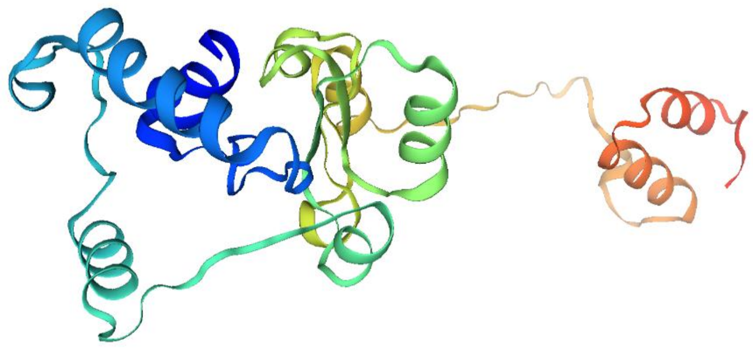

SlSSADH-2

Fig. S17. Proteins modeled with swiss-model

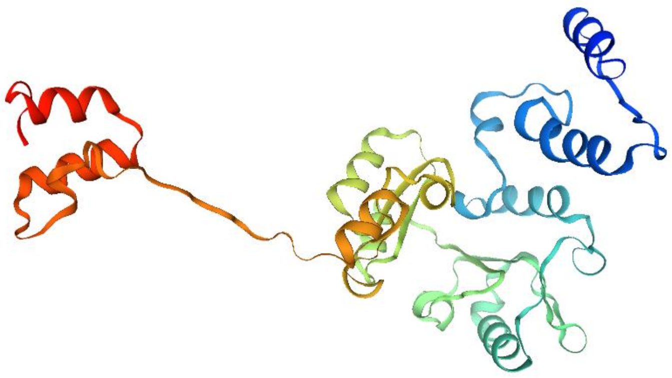

SlSSADH-3

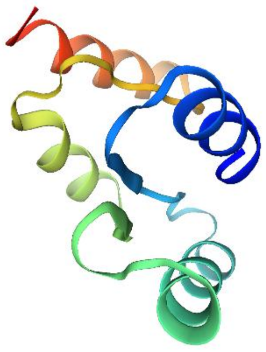

SlSSADH-4

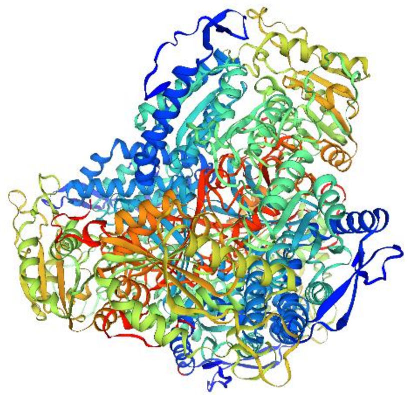

SlSSADH-5

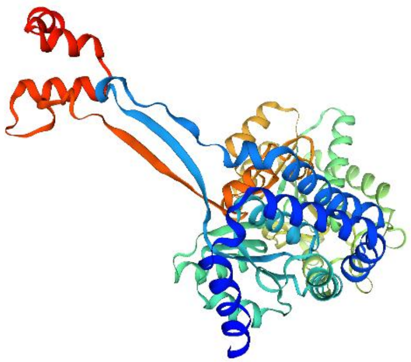

SlSSADH-6

Fig. S18. Proteins modeled with swiss-model

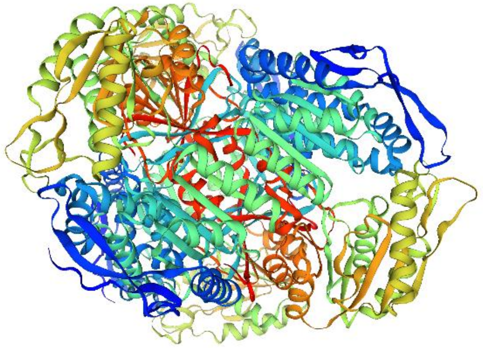

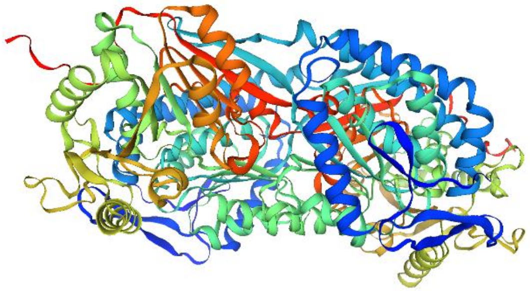

SlSSADH-8

SlSSADH-7

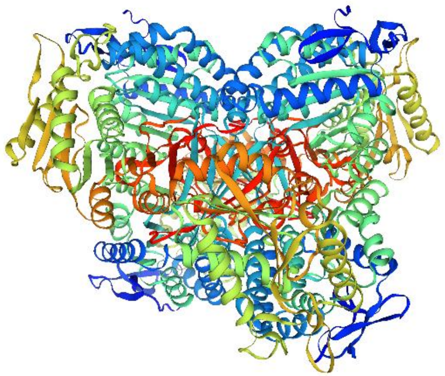

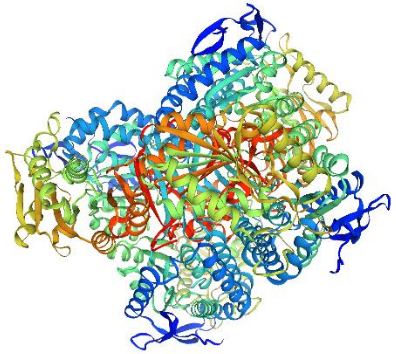

SlSSADH-9

SlSSADH-10

Fig. S19. Proteins modeled with swiss-model

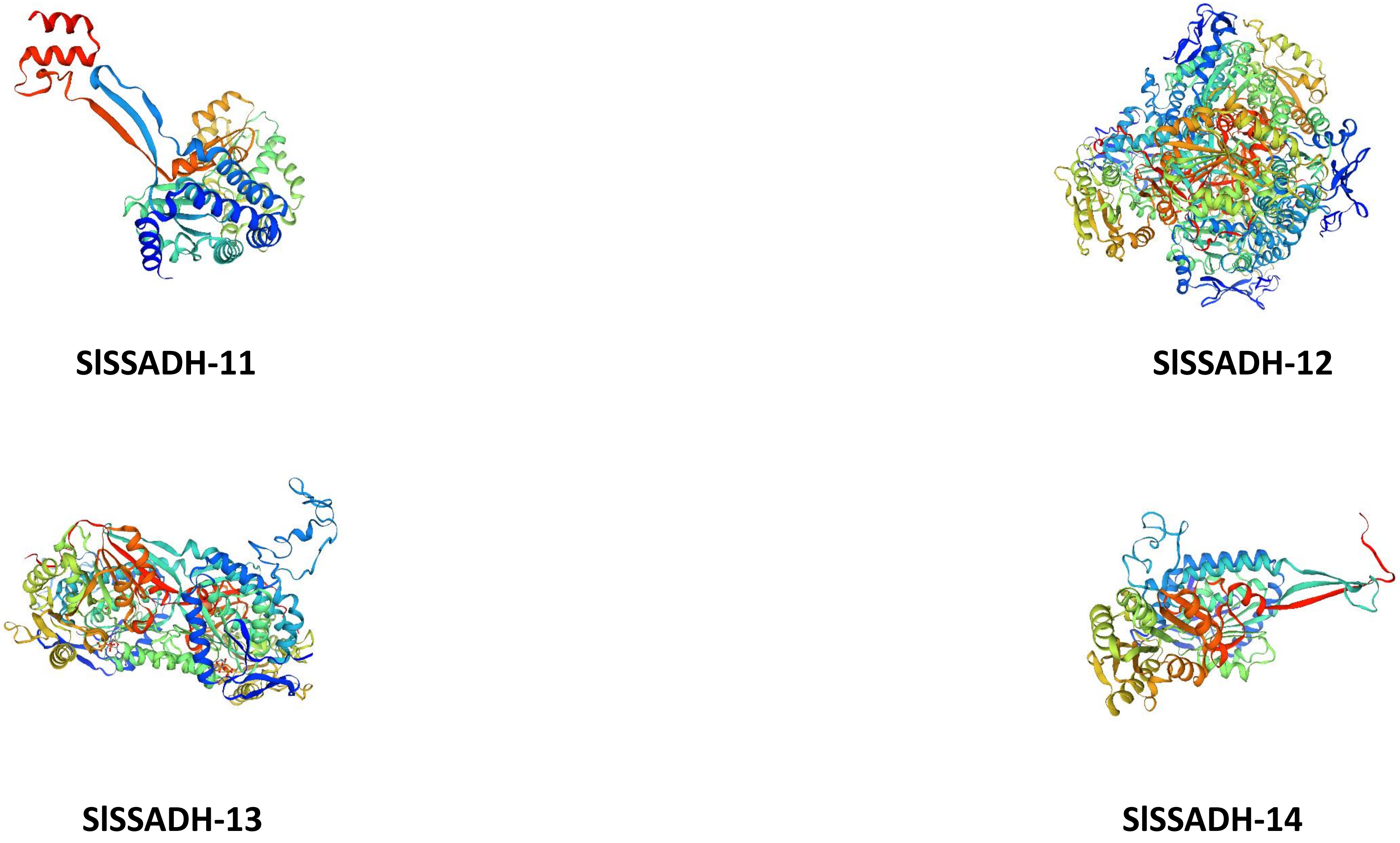

Fig. S20. Proteins modeled with swiss-model

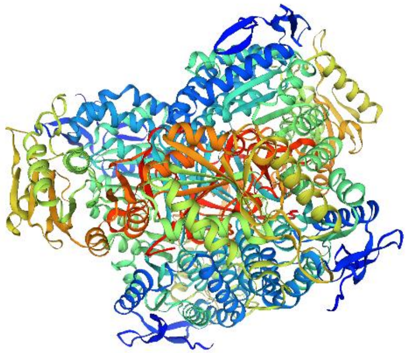

SlSSADH-15

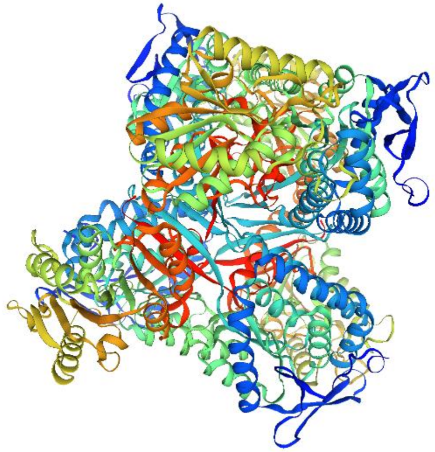

SlSSADH-16

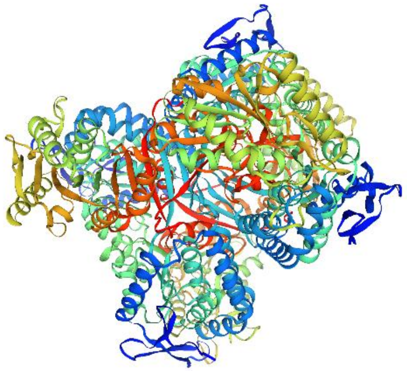

SlSSADH-17

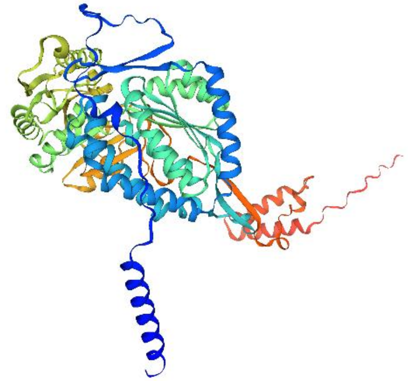

SlSSADH-18

Fig. S21. Proteins modeled with swiss-model

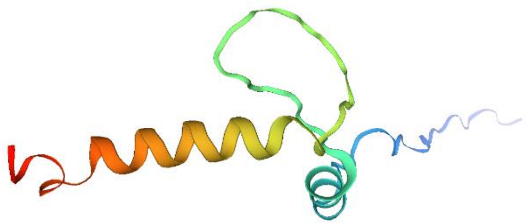
SlDHN-1

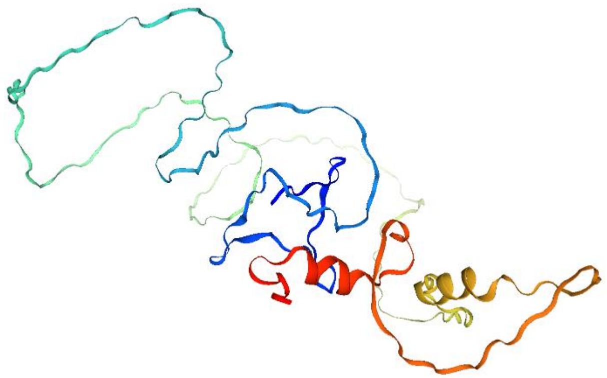
SlDHN-2

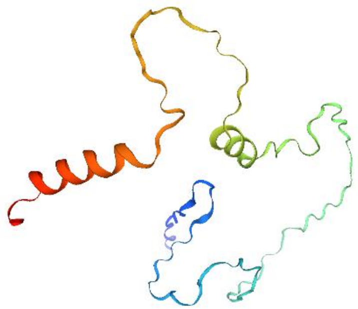
SlDHN-3

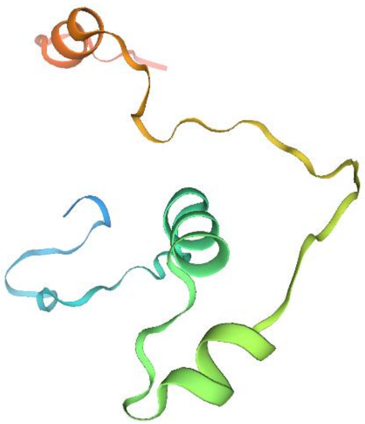
SlDHN-4

Fig. S22. Proteins modeled with swiss-model

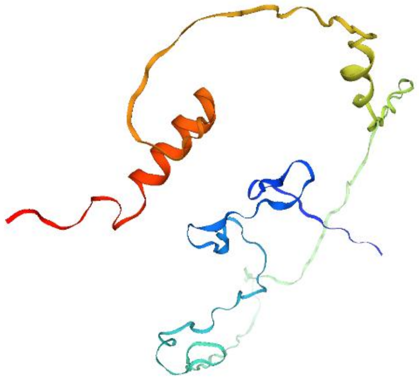

SlDHN-5

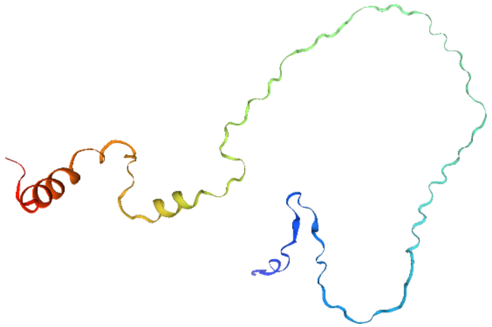

SlDHN-6

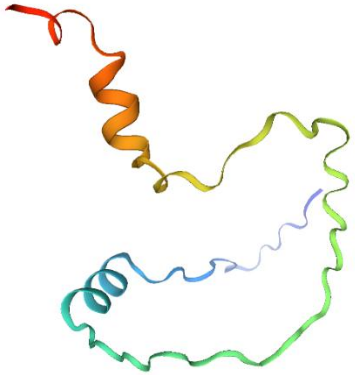

SlDHN-7

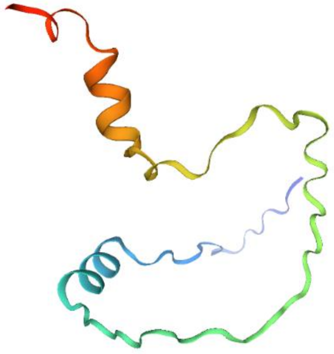

SlDHN-8

Fig. S23. Proteins modeled with swiss-model

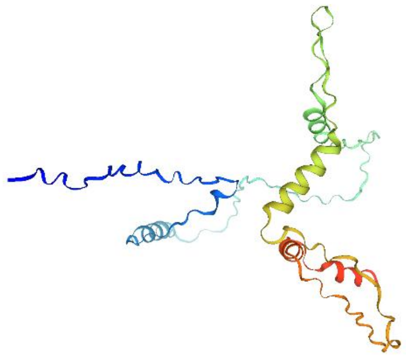

SlDHN-9

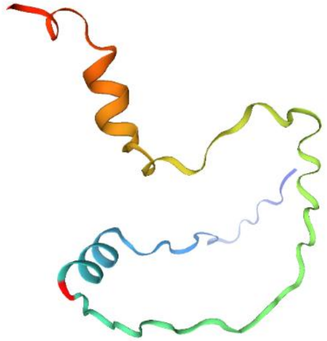

SlDHN-10

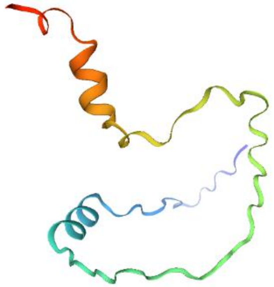

SlDHN-11

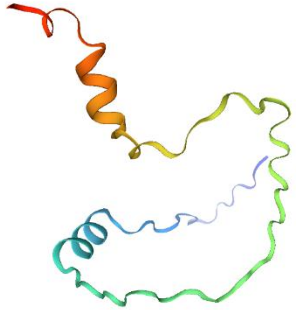

SlDHN-12

Fig. S24. Proteins modeled with swiss-model

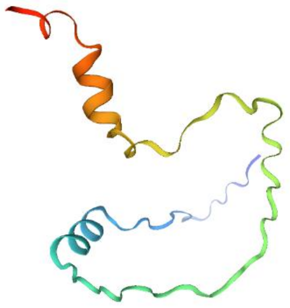

SlDHN-13

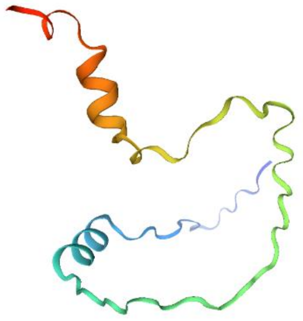

SlDHN-14

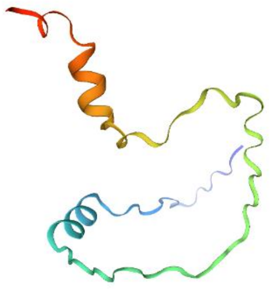

SlDHN-15

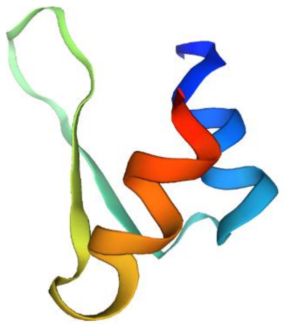

SlDHN-16

Fig. S25. Proteins modeled with swiss-model

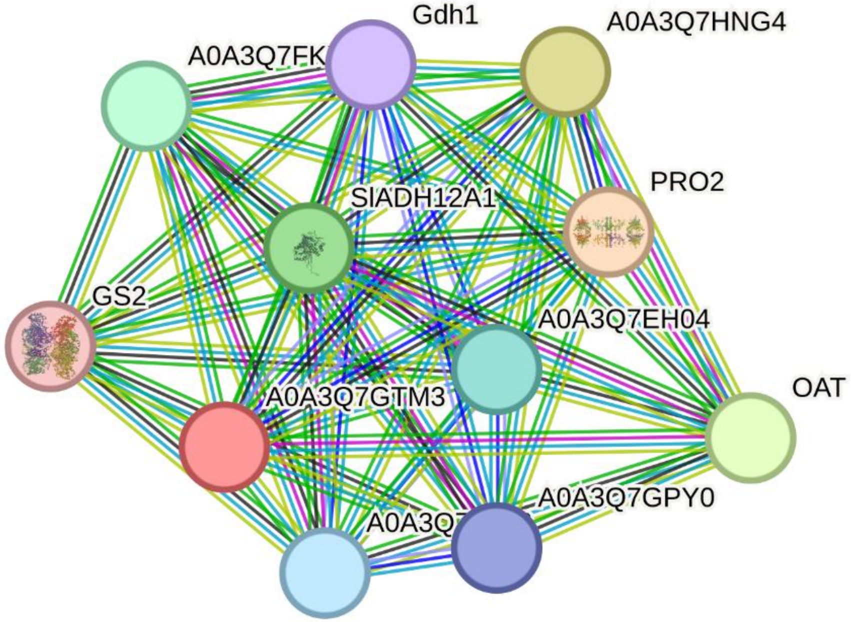

SlP5CS-1

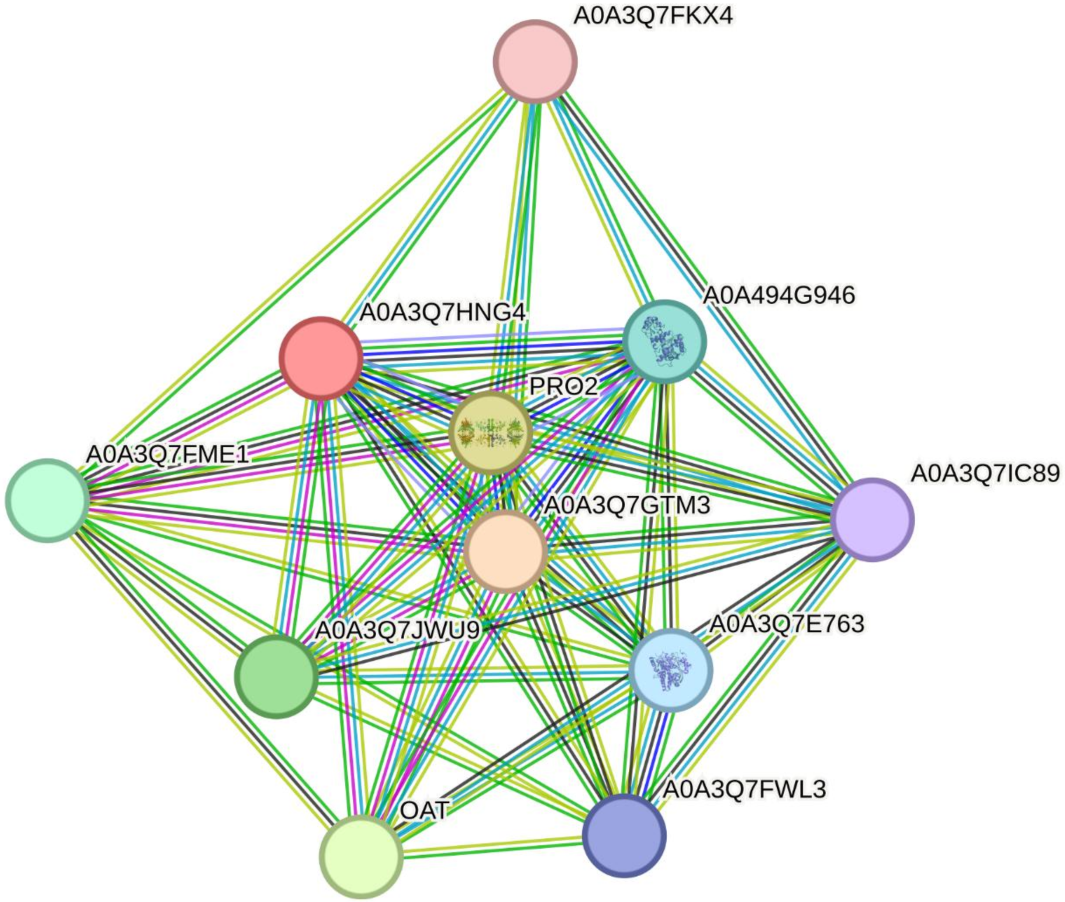

SlP5CS-2

Fig. S26. Protein-protein interaction (PPI) network of SlP5CS clusters

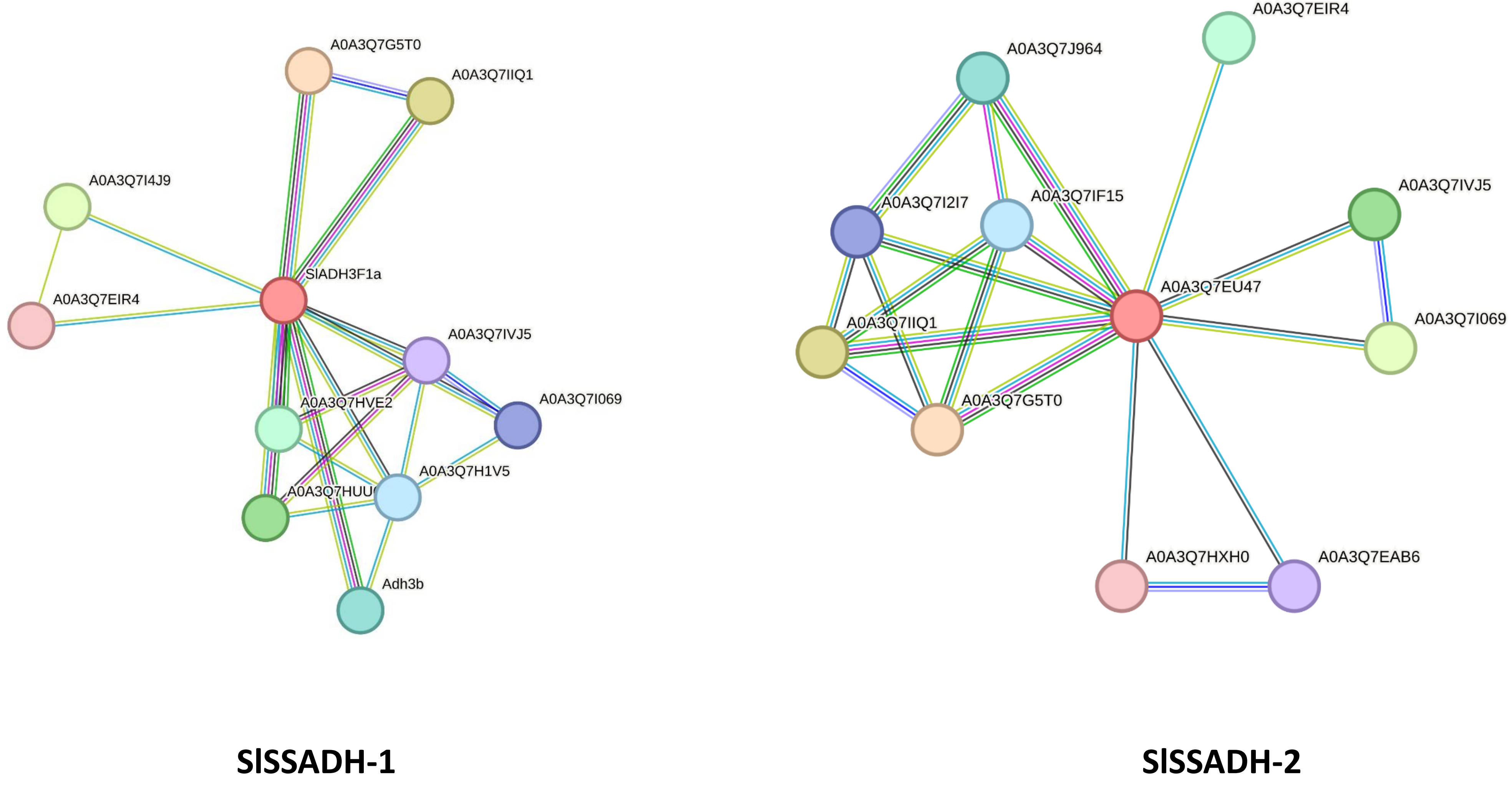

Fig. S27. Protein-protein interaction (PPI) network of SlSSADH clusters

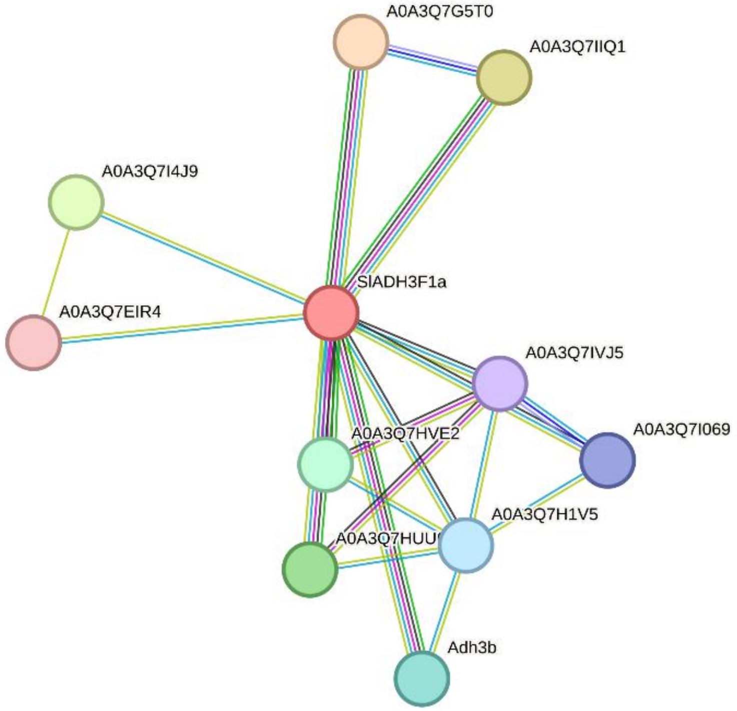

SlSSADH-3

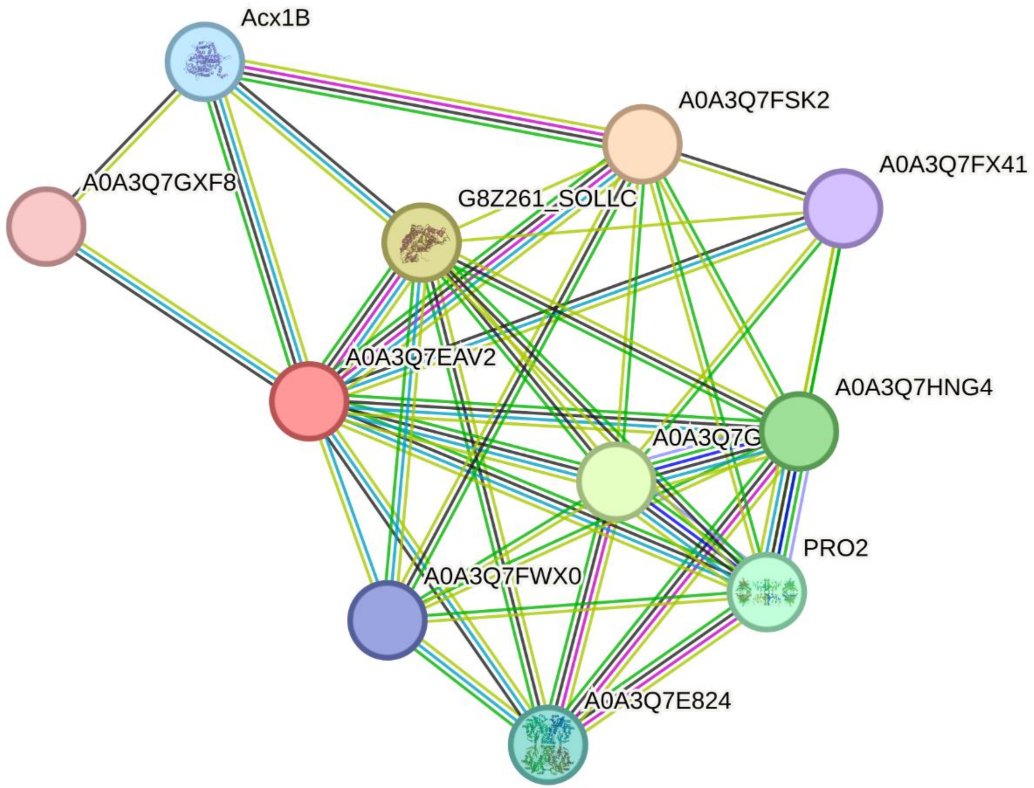

SlSSADH-4

Fig. S28. Protein-protein interaction (PPI) network of SlSSADH clusters

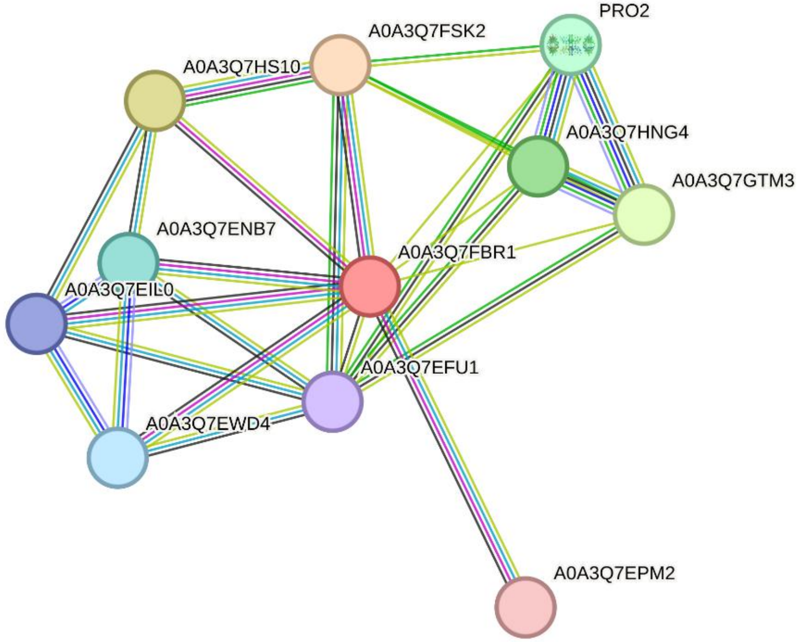

SlSSADH-5

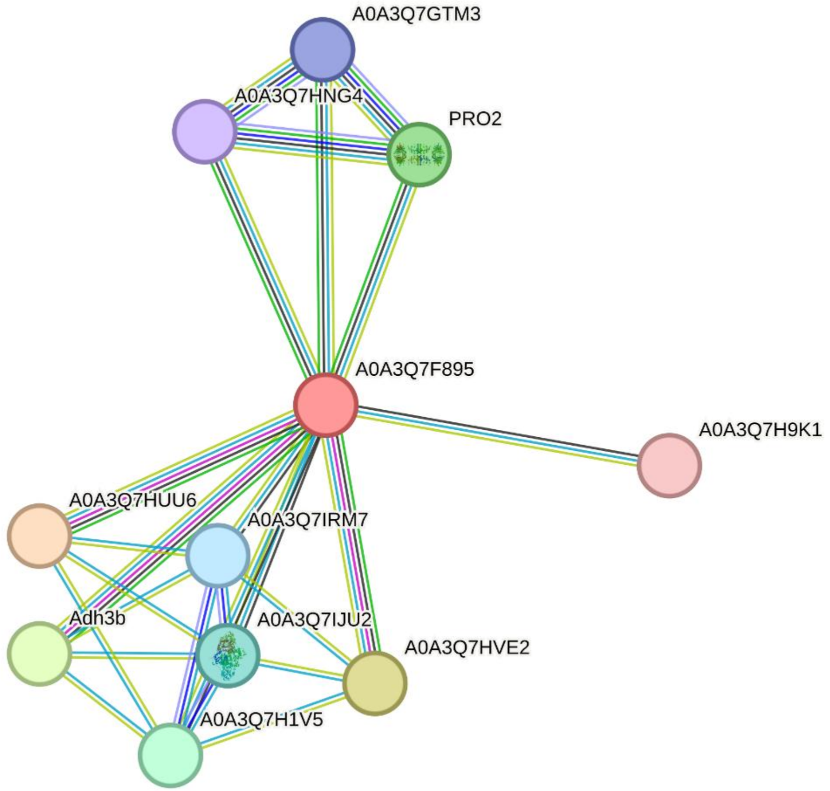

SlSSADH-6

Fig. S29. Protein-protein interaction (PPI) network of SlSSADH clusters

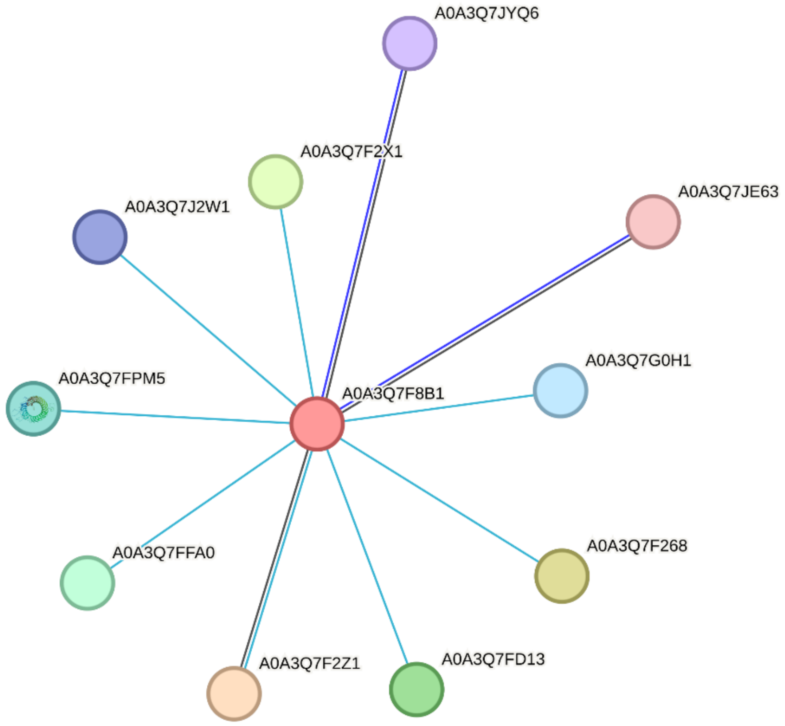

SlSSADH-7

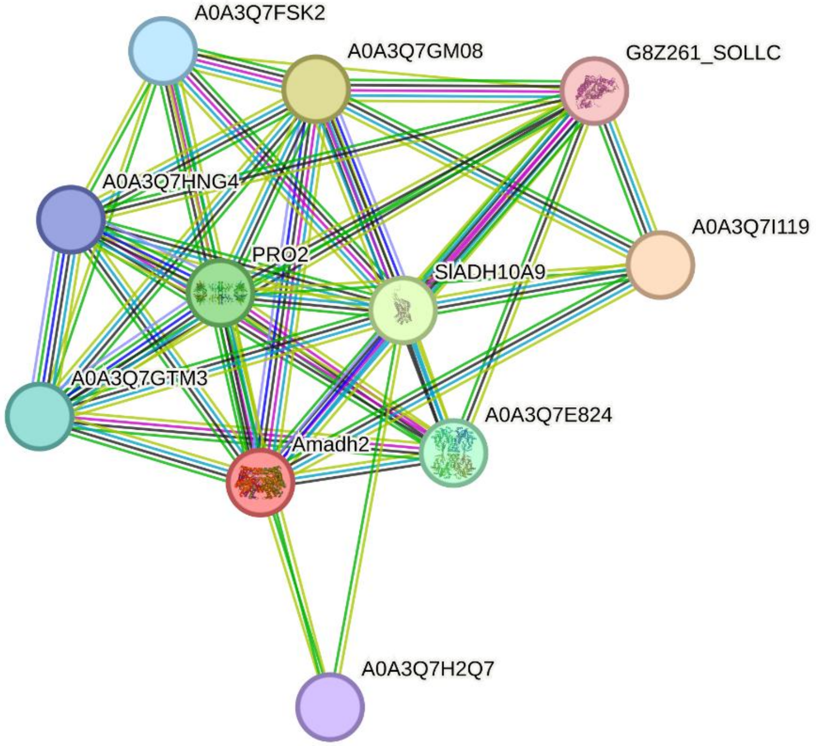

SlSSADH-8

Fig. S30. Protein-protein interaction (PPI) network of SlSSADH clusters

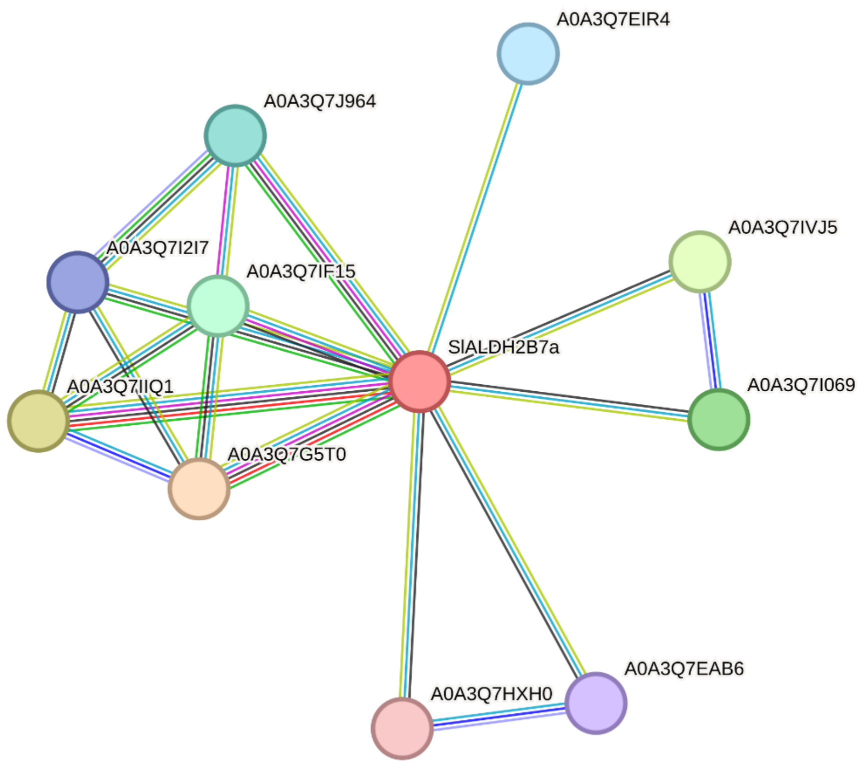

SlSSADH-9

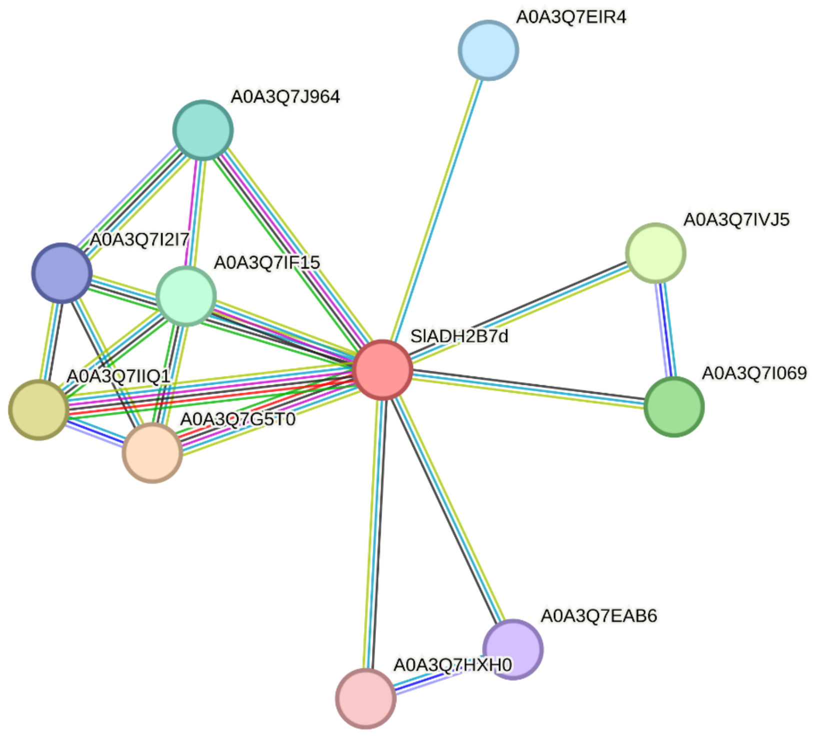

SlSSADH-10

Fig. S31. Protein-protein interaction (PPI) network of SlSSADH clusters

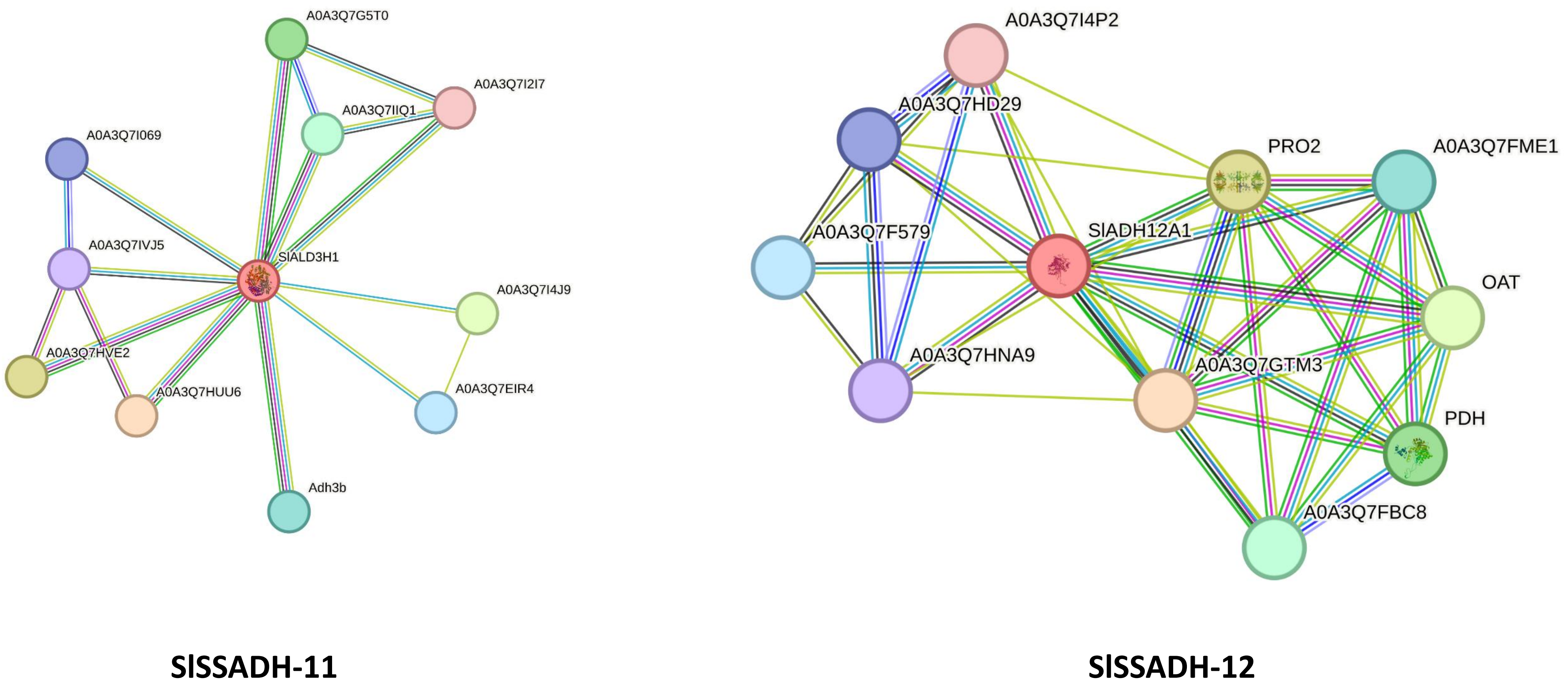

Fig. S32. Protein-protein interaction (PPI) network of SlSSADH clusters

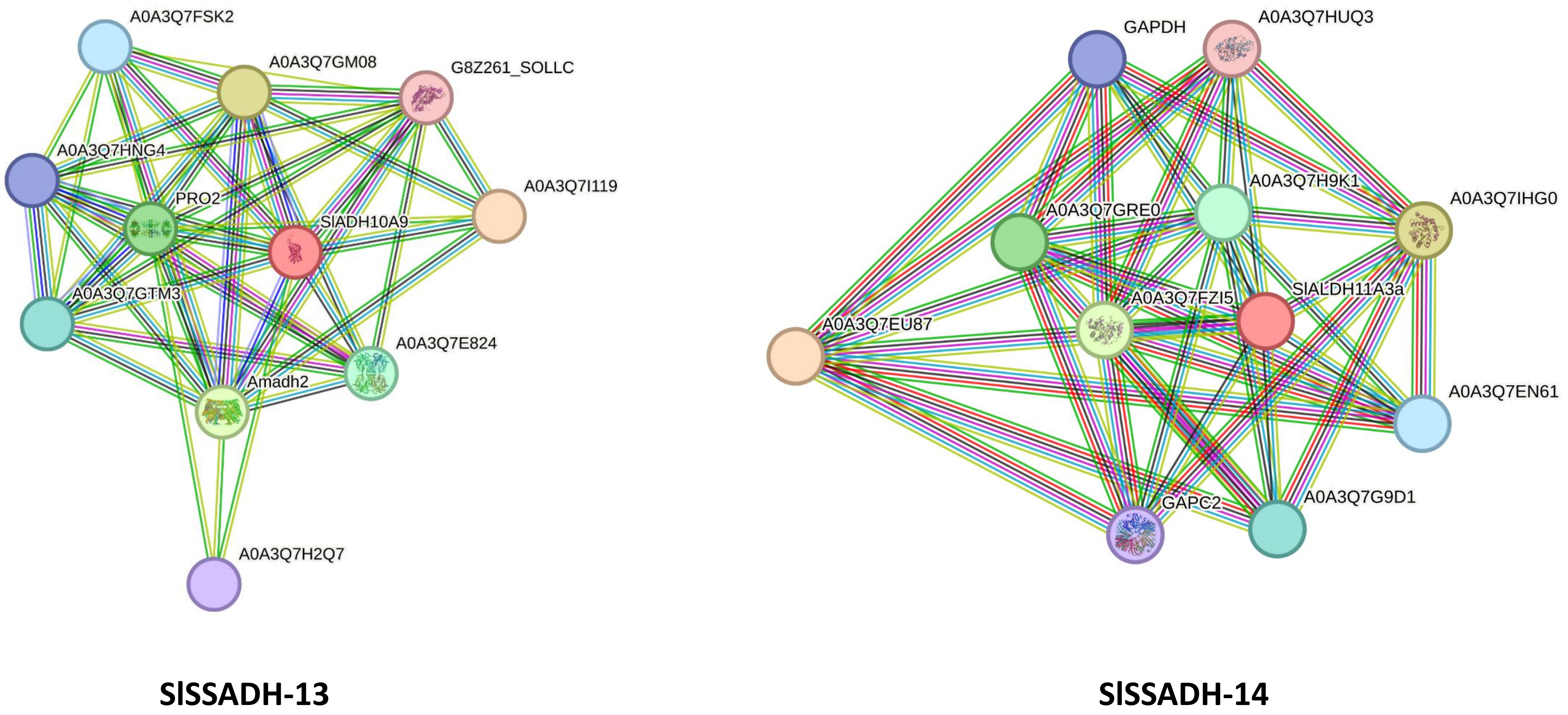

Fig. S33. Protein-protein interaction (PPI) network of SlSSADH clusters

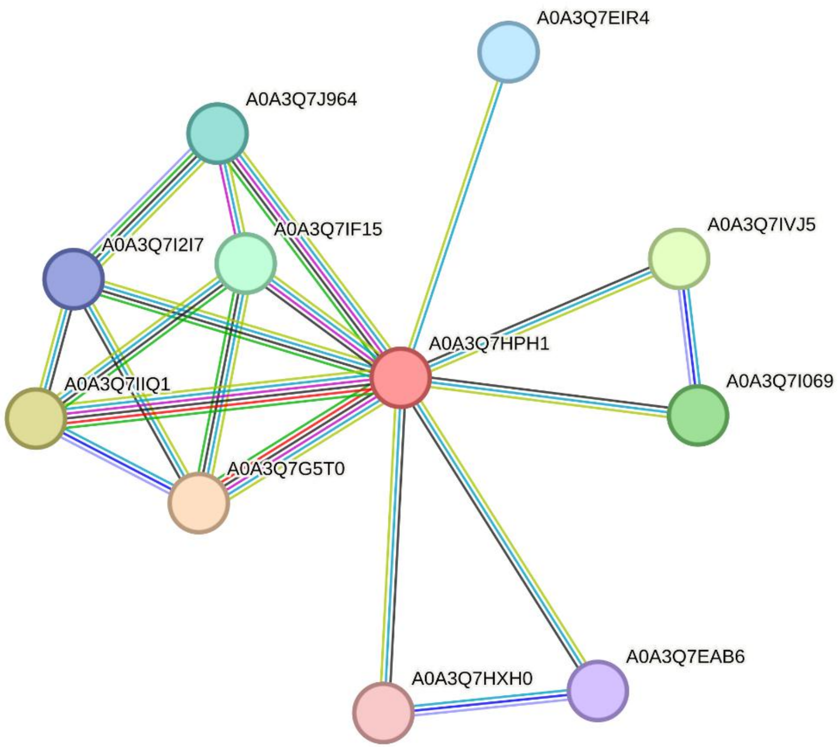

SlSSADH-15

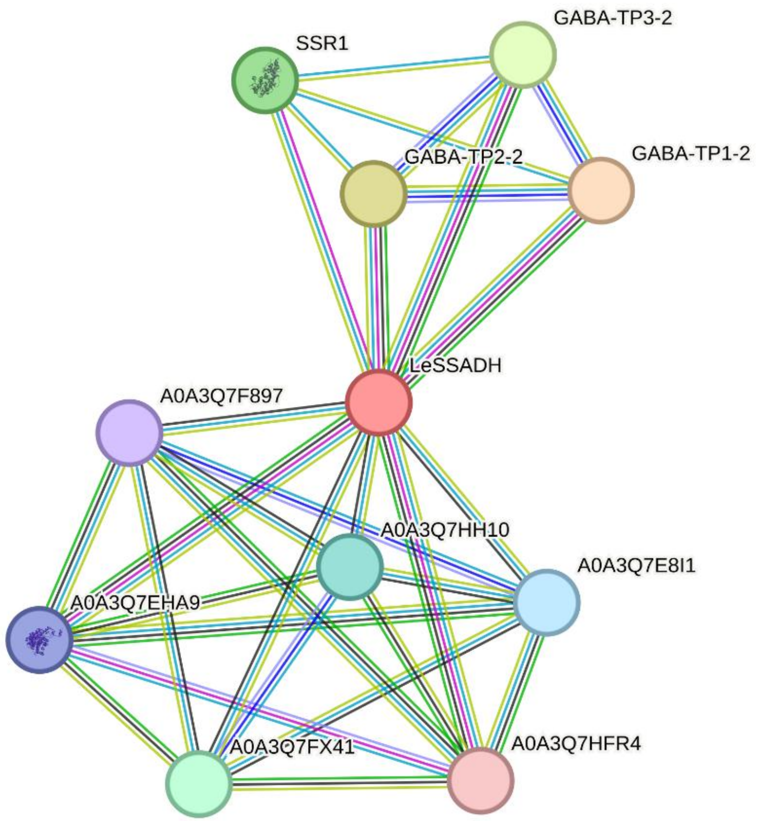

SlSSADH-16

Fig. S34. Protein-protein interaction (PPI) network of SlSSADH clusters

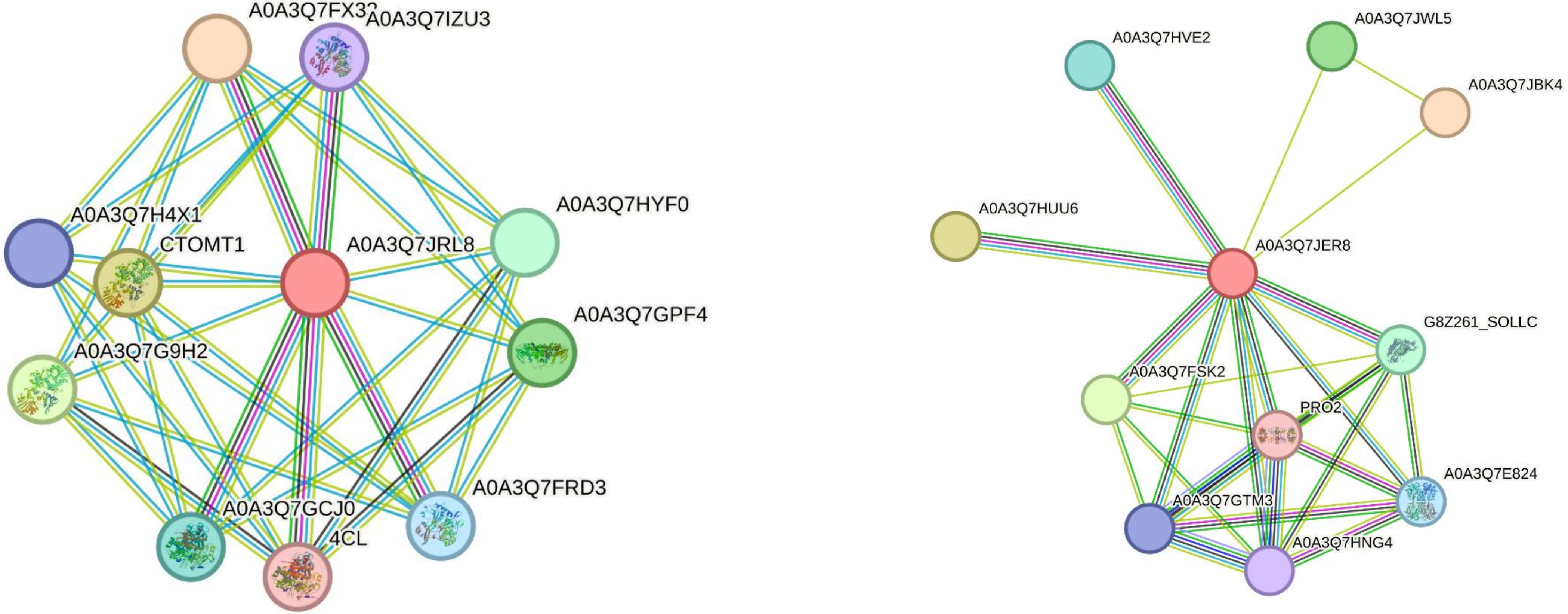

SlSSADH-17 **SlSSADH-18**

Fig. S35. Protein-protein interaction (PPI) network of SlSSADH clusters

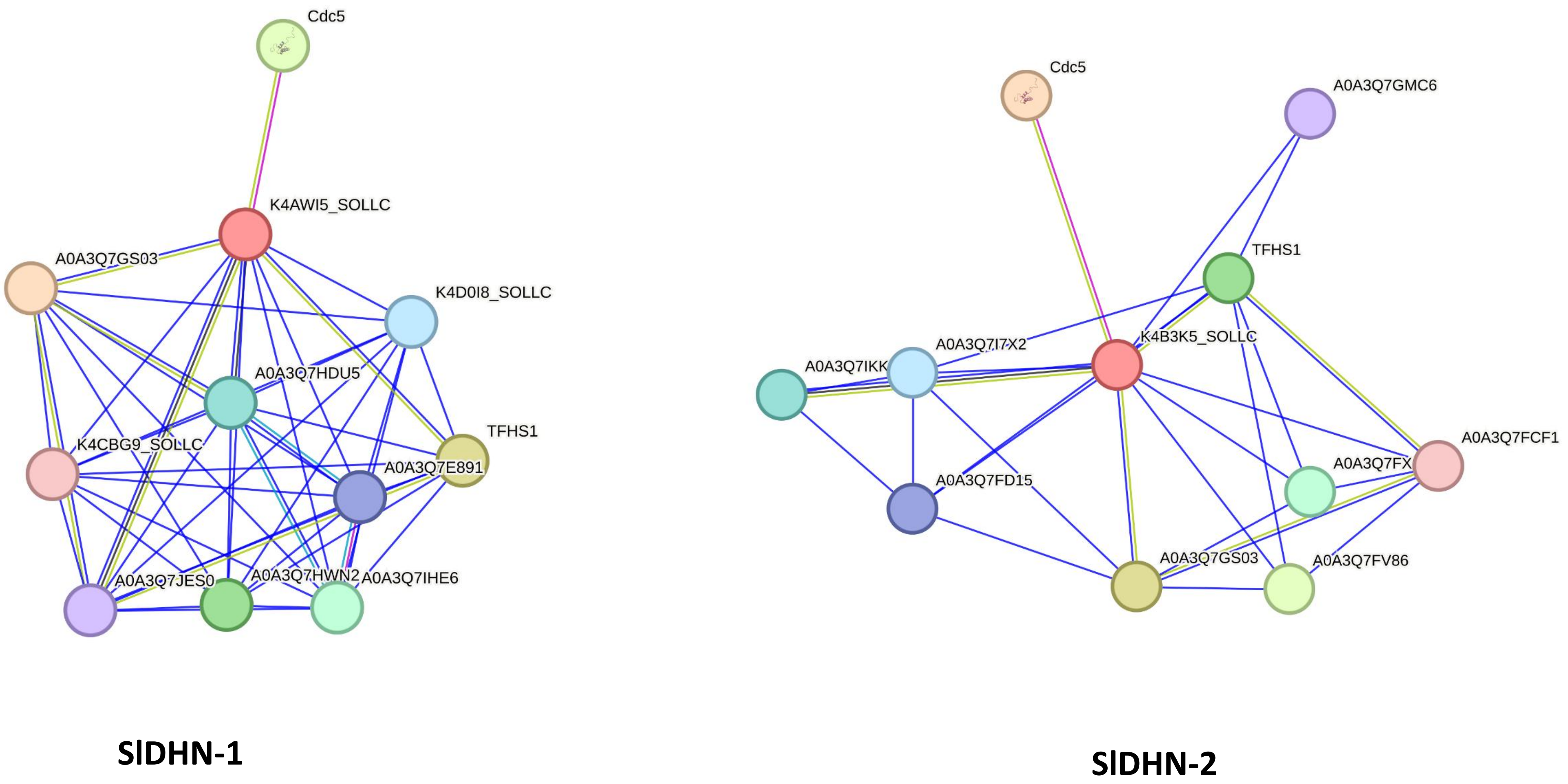

Fig. S36. Protein-protein interaction (PPI) network of Dehydrin clusters

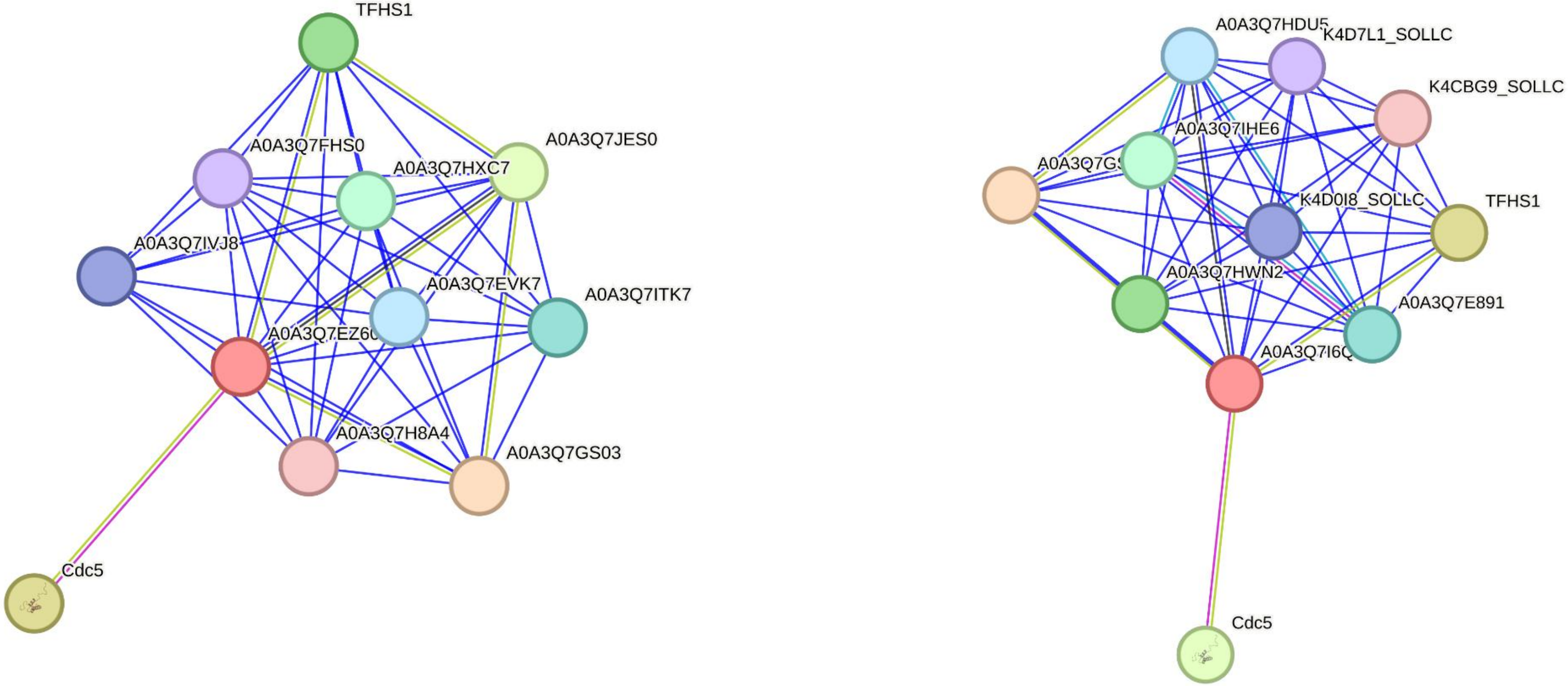

SlDHN-3

SlDHN-4

Fig. S37. Protein-protein interaction (PPI) network of Dehydrin clusters

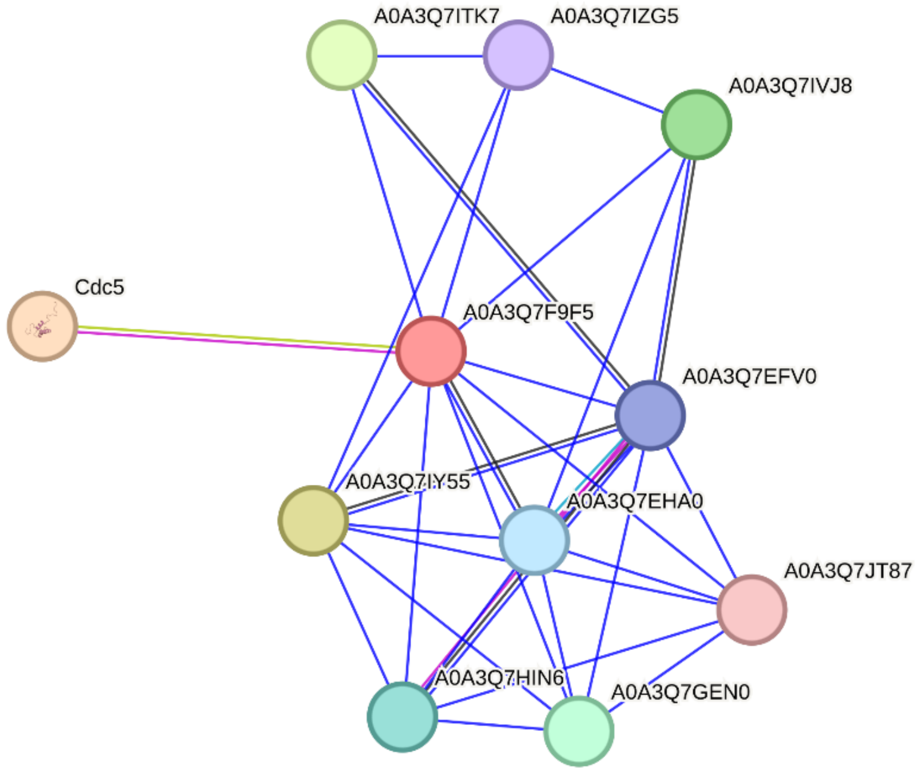

SlDHN-5

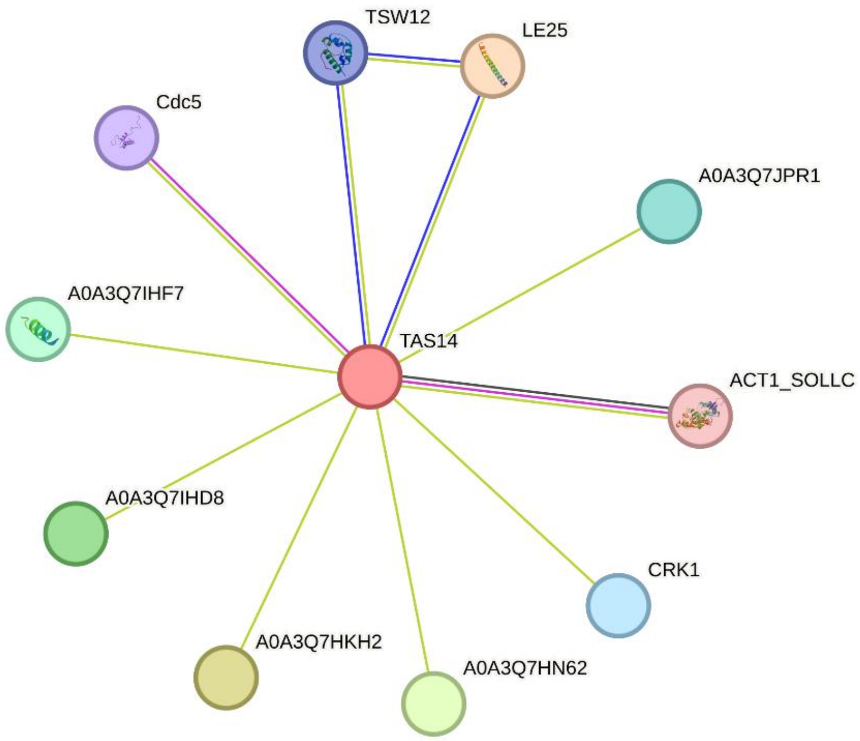

SlDHN-6

Fig. S38. Protein-protein interaction (PPI) network of Dehydrin clusters

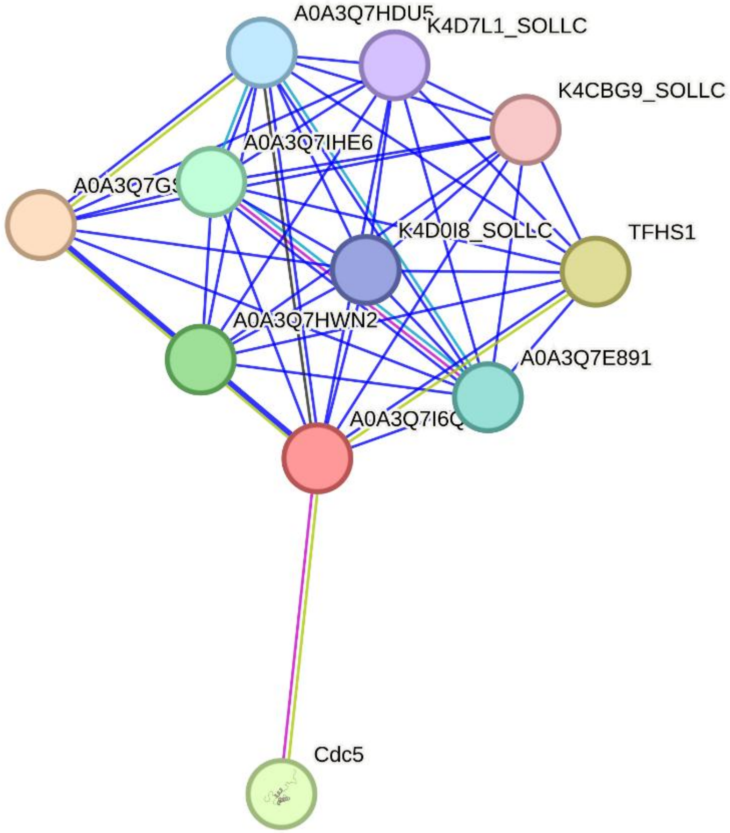

SlDHN-7

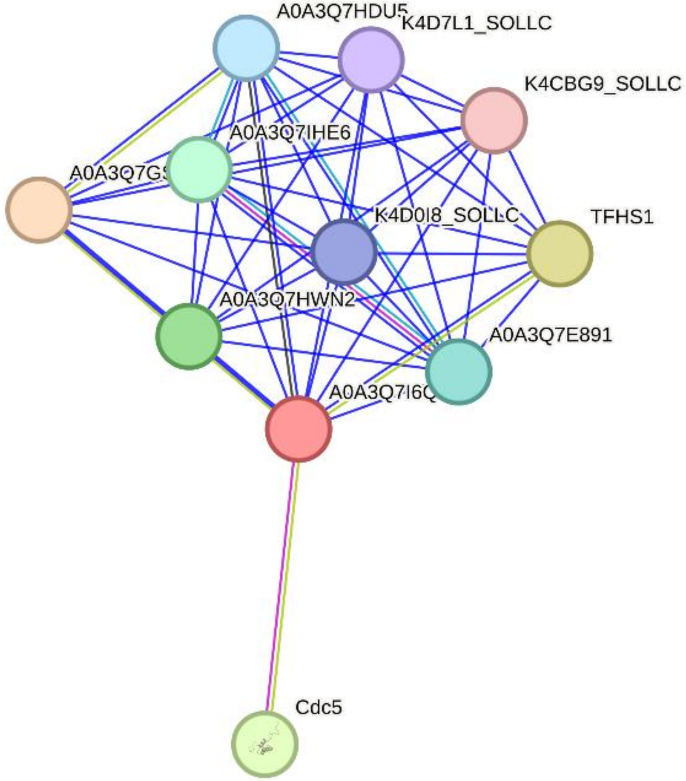

SlDHN-8

Fig. S39. Protein-protein interaction (PPI) network of Dehydrin clusters

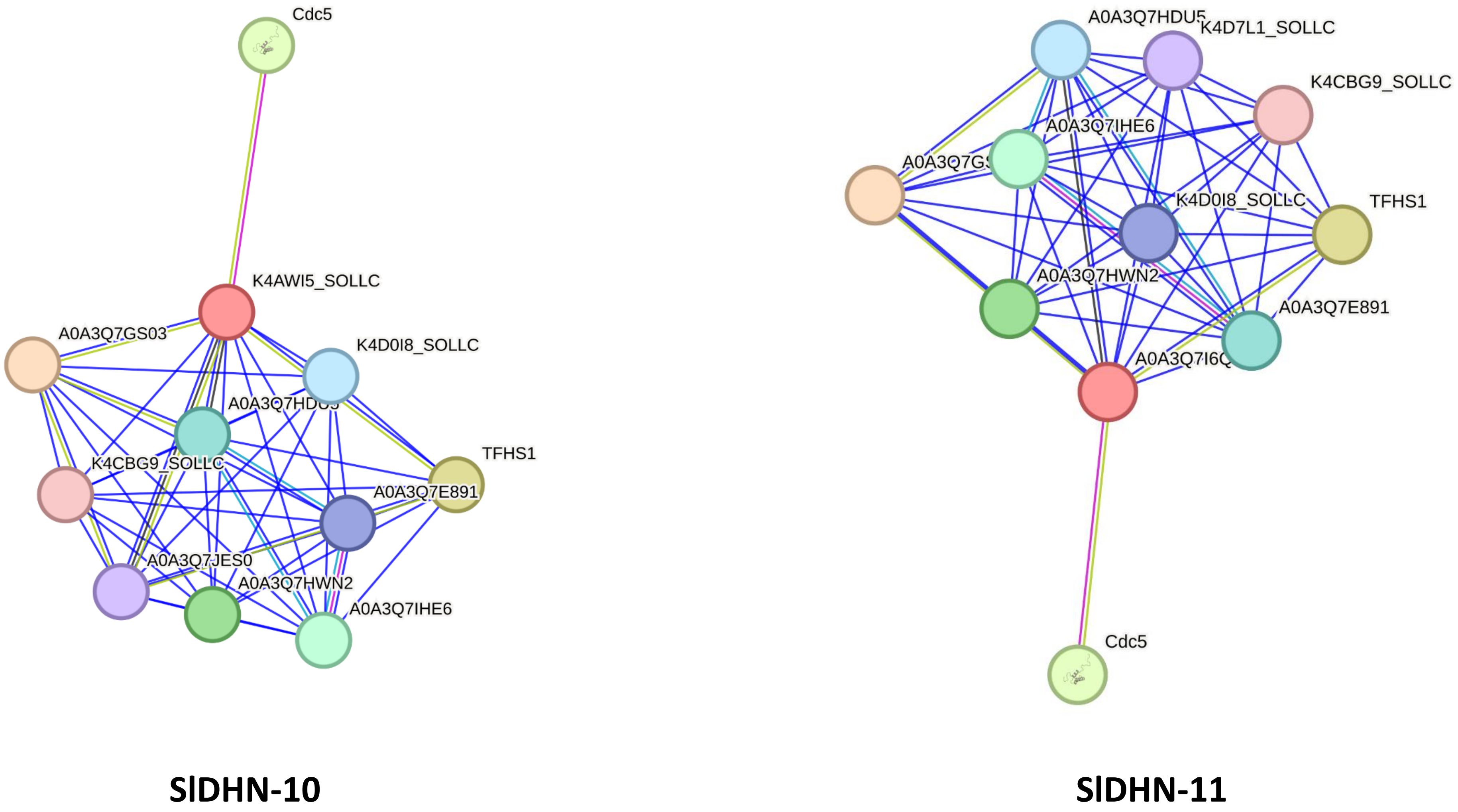

Fig. S40. Protein-protein interaction (PPI) network of Dehydrin clusters

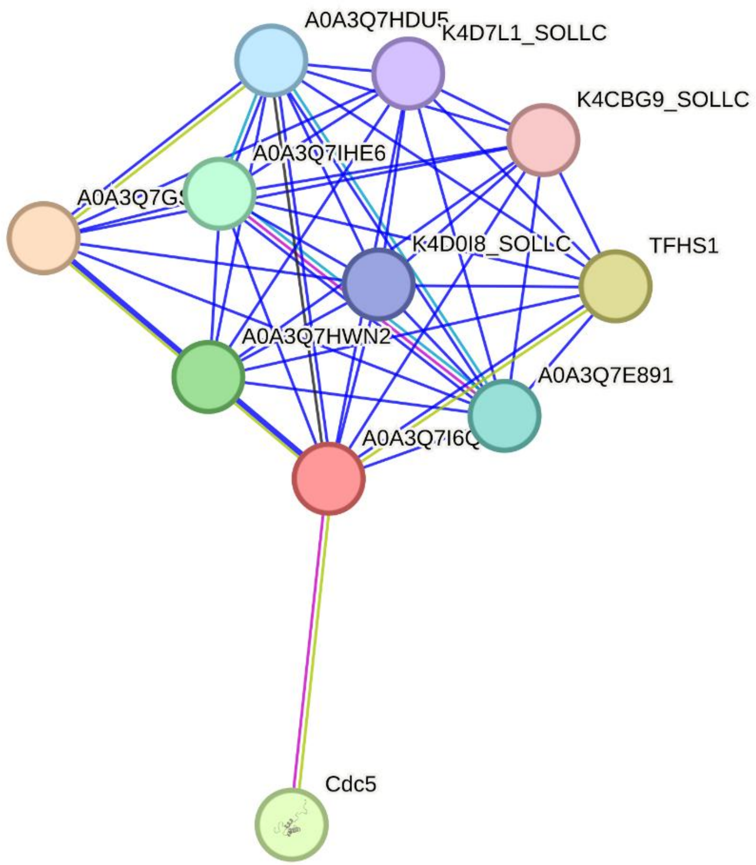

SlDHN-12

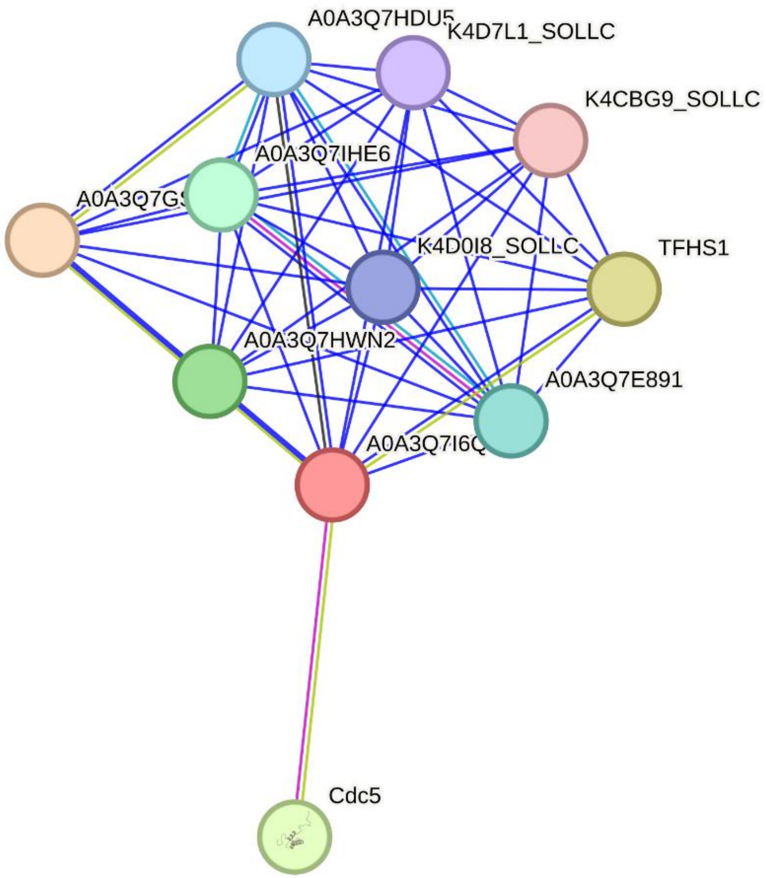

SlDHN-13

Fig. S41. Protein-protein interaction (PPI) network of Dehydrin clusters

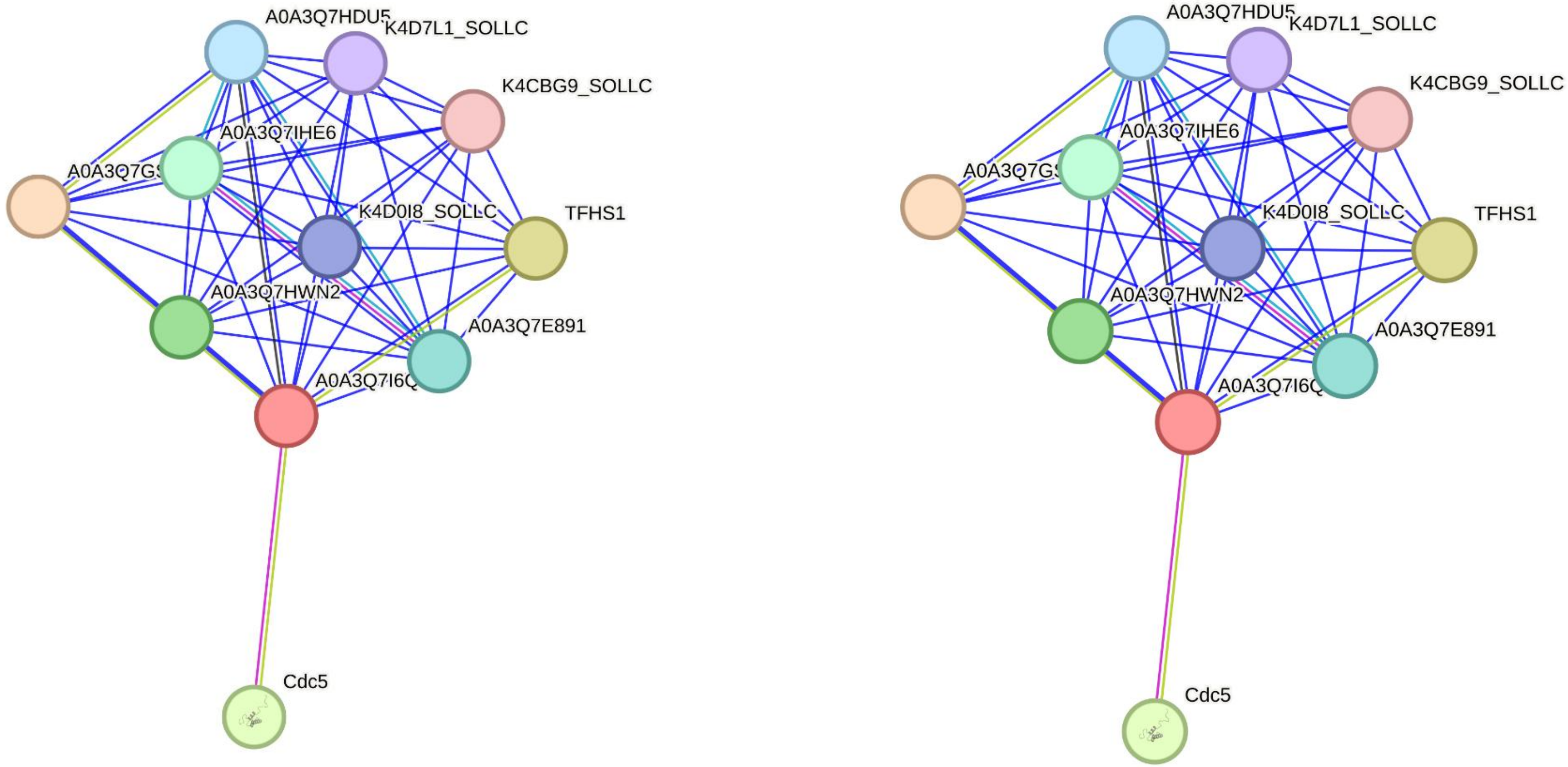

SlDHN-14

SlDHN-15

Fig. S42. Protein-protein interaction (PPI) network of Dehydrin clusters

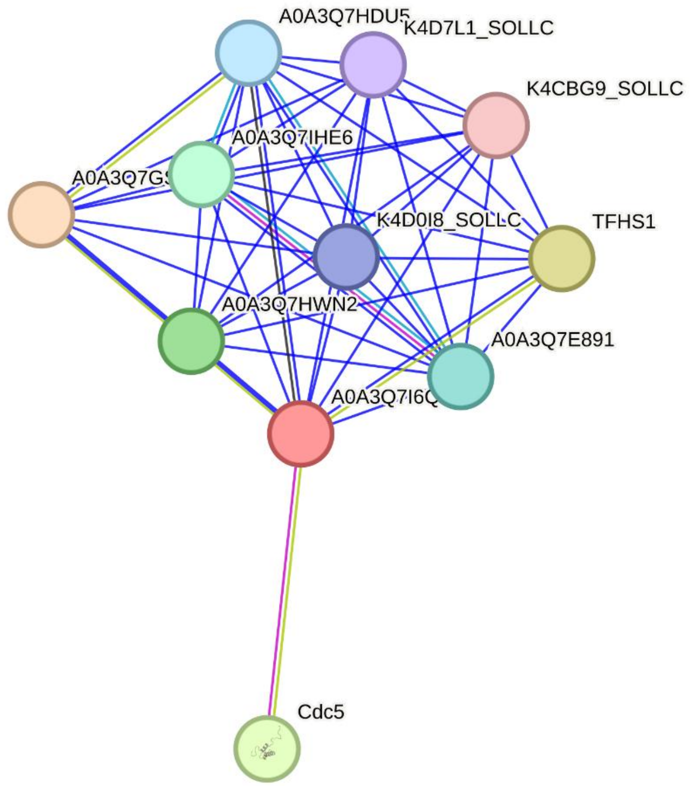

SlDHN-16

Fig. S43. Protein-protein interaction (PPI) network of Dehydrin clusters

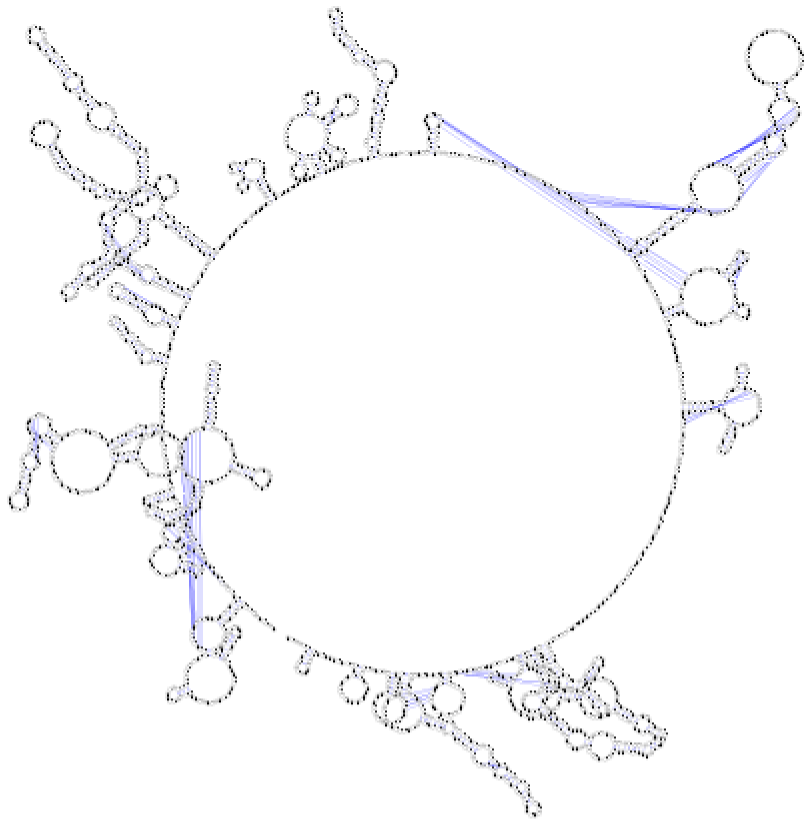

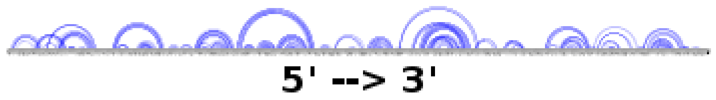

Fig. S44. Predicting RNA secondary structures of SlP5CS-1

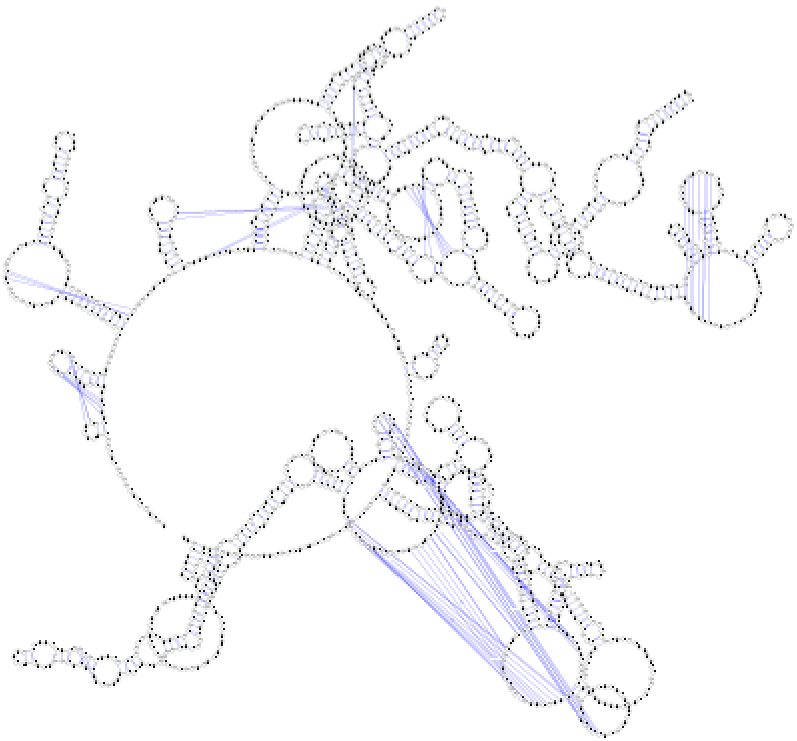

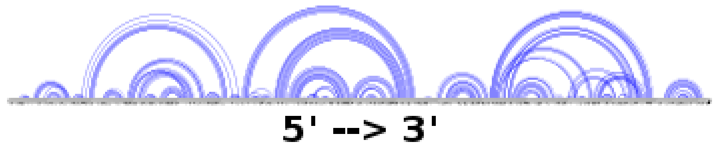

Fig. S45. Predicting RNA secondary structures of SlSSADH-5

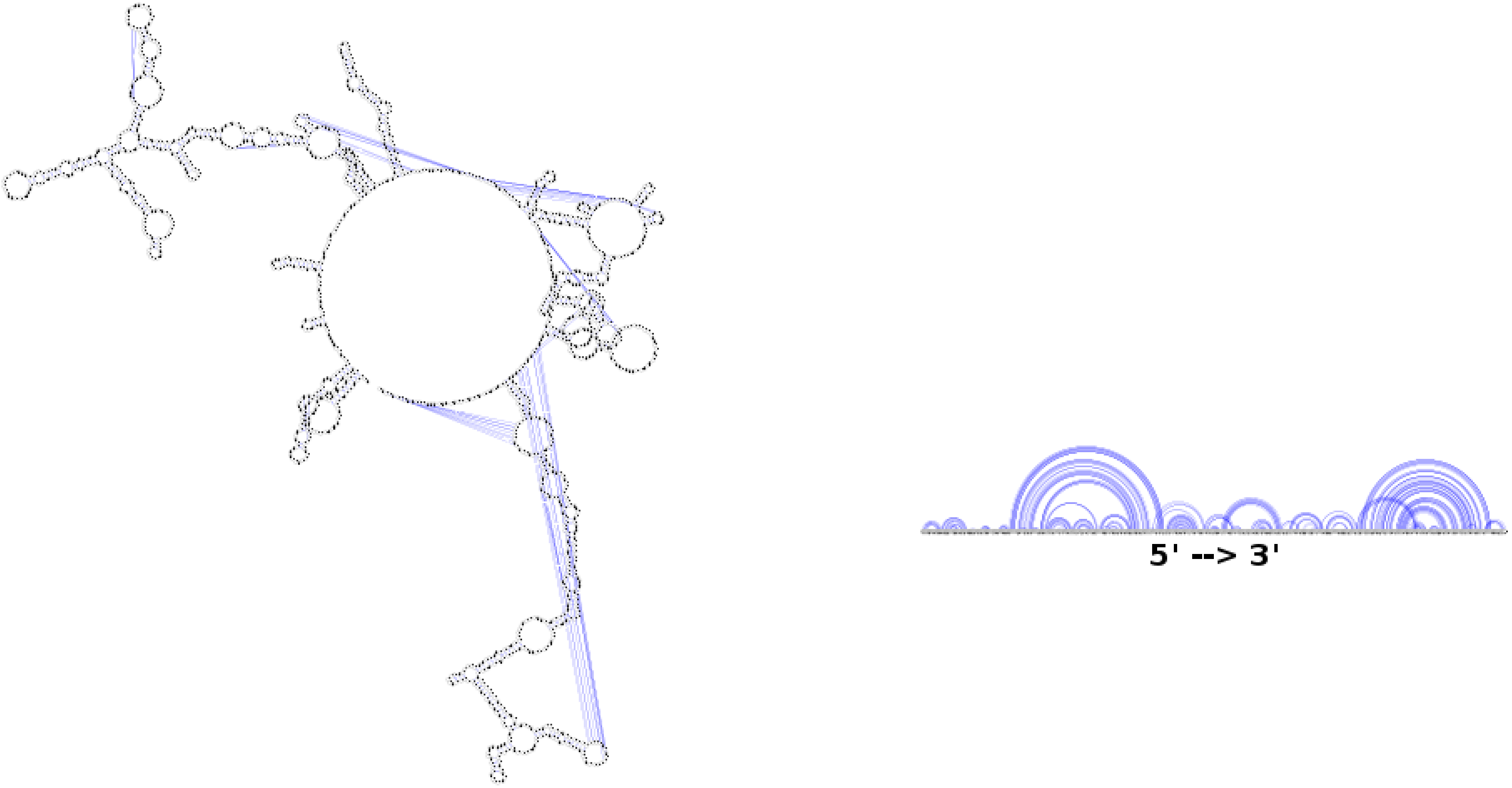

Fig. S46. Predicting RNA secondary structures of SlSSADH-6

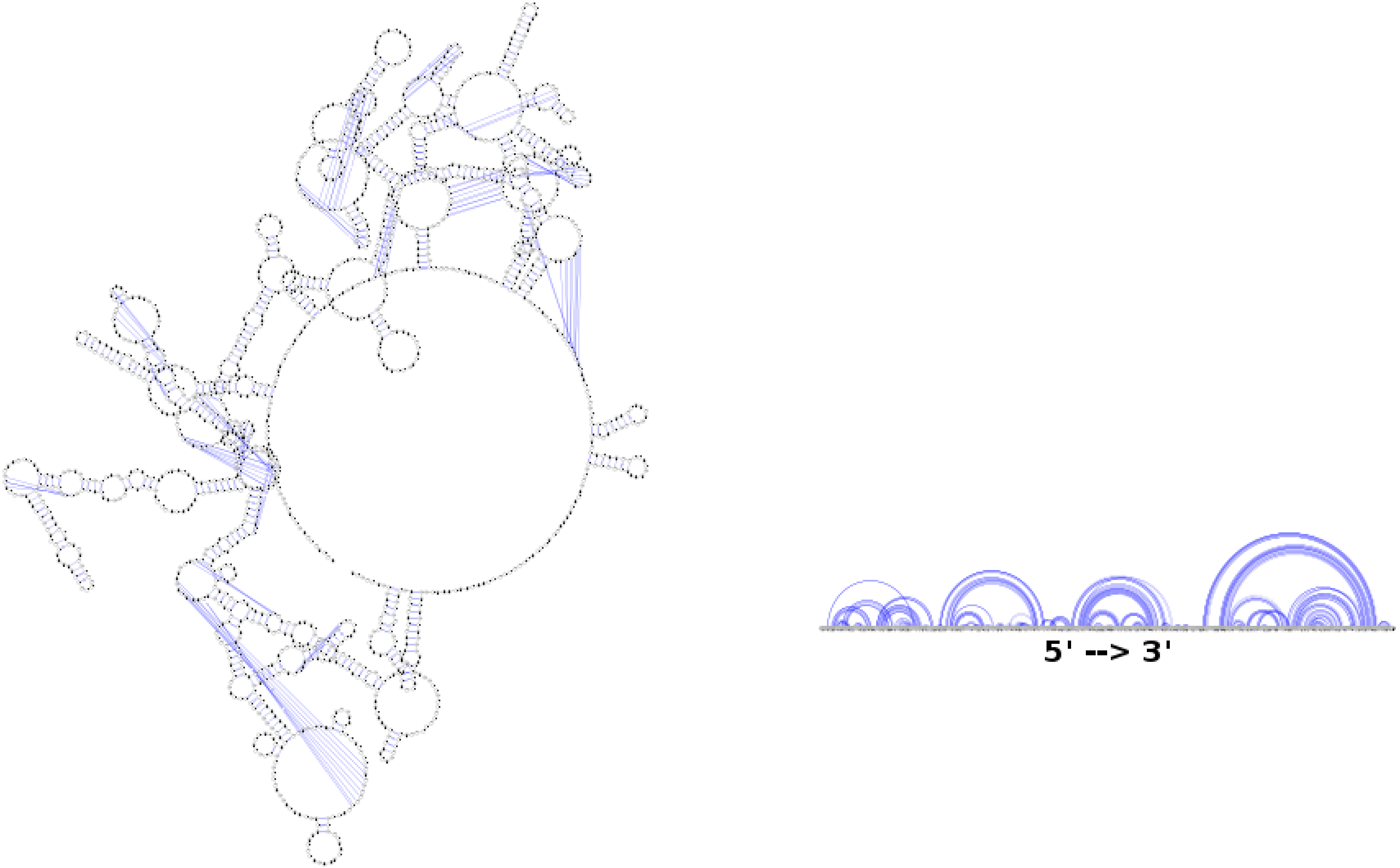

Fig. S47. Predicting RNA secondary structures of SlSSADH-12

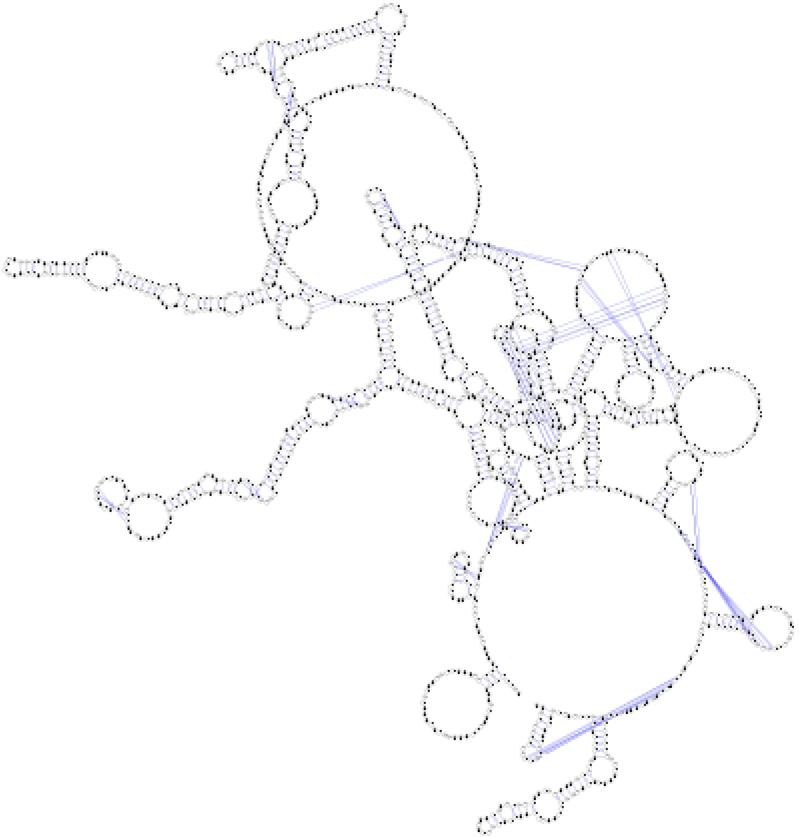

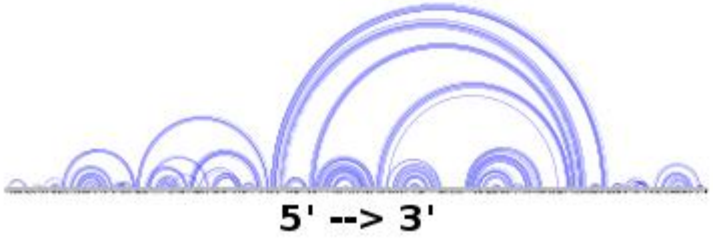

Fig. S48. Predicting RNA secondary structures of SlSSADH-17

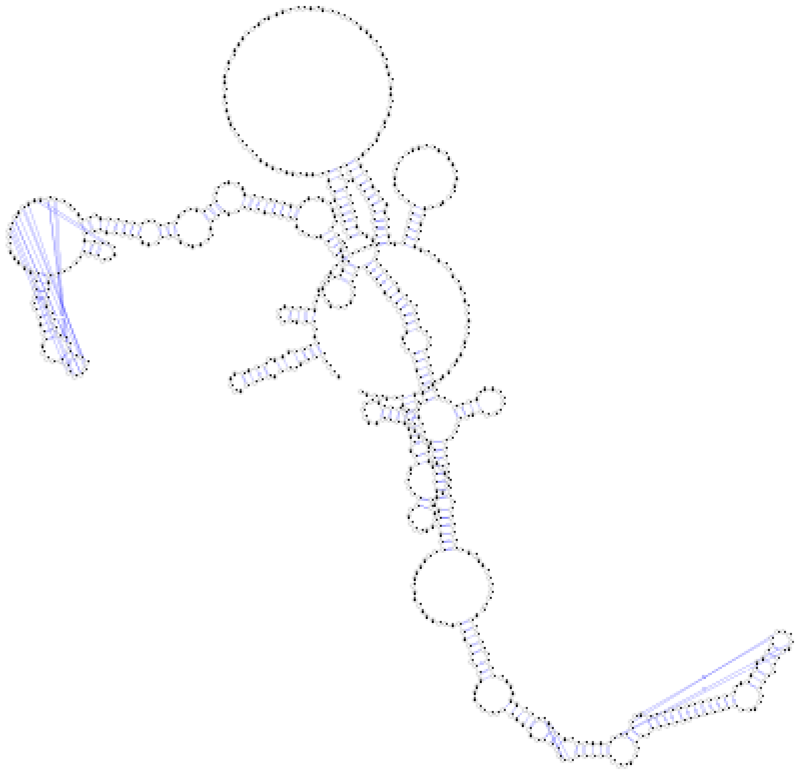

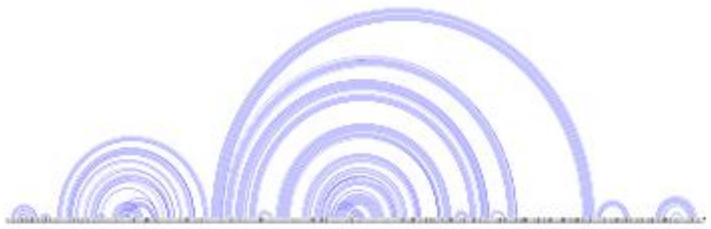

Fig. S49. Predicting RNA secondary structures of SlDHN-2

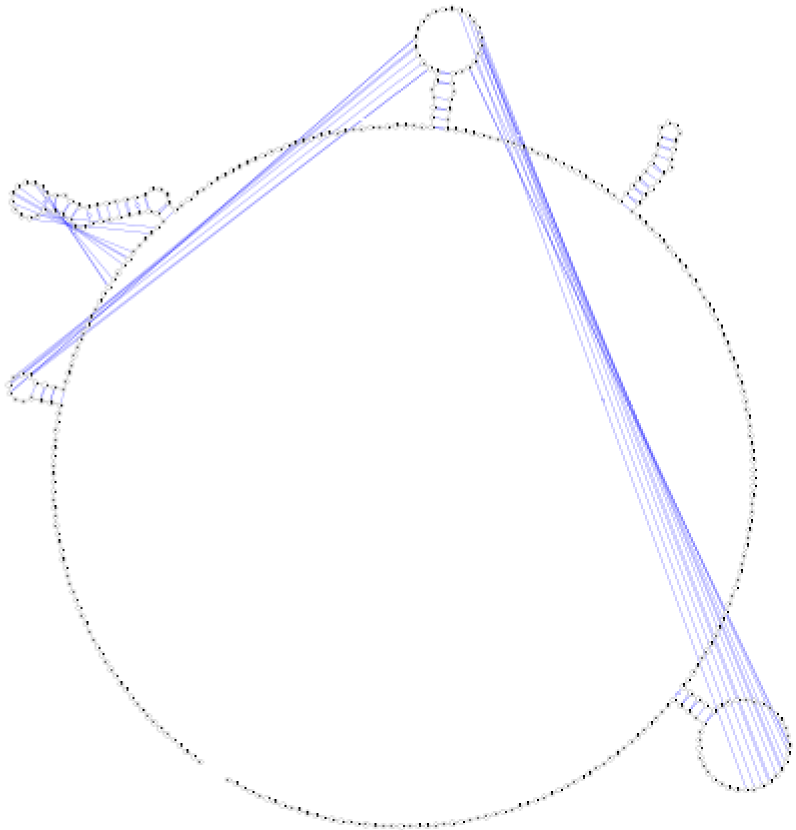

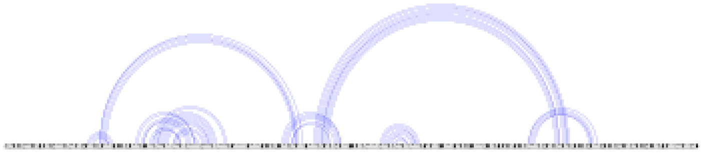

Fig. S50. Predicting RNA secondary structures of SlDHN-6

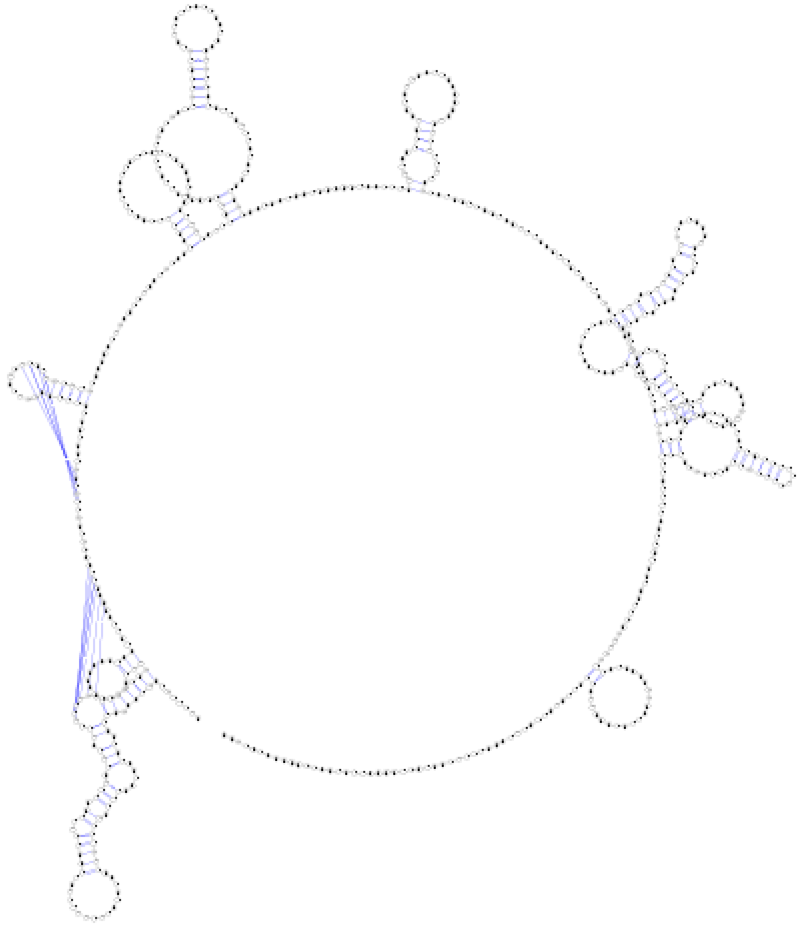

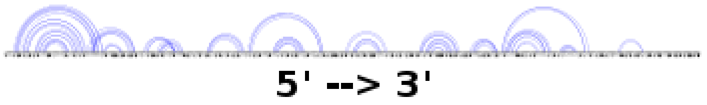

Fig. S51. Predicting RNA secondary structures of SlDHN-9

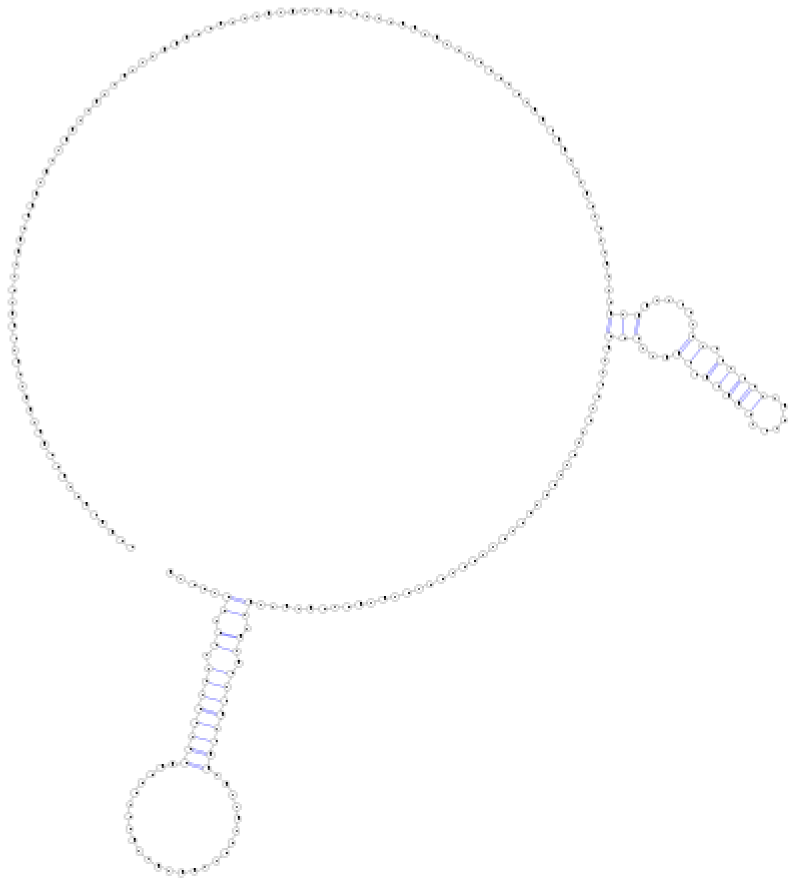

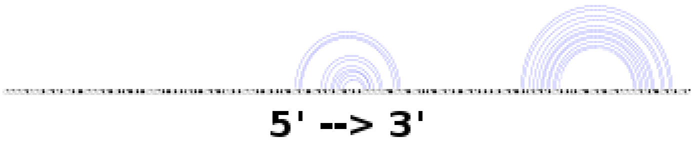

Fig. S52. Predicting RNA secondary structures of SlDHN-14

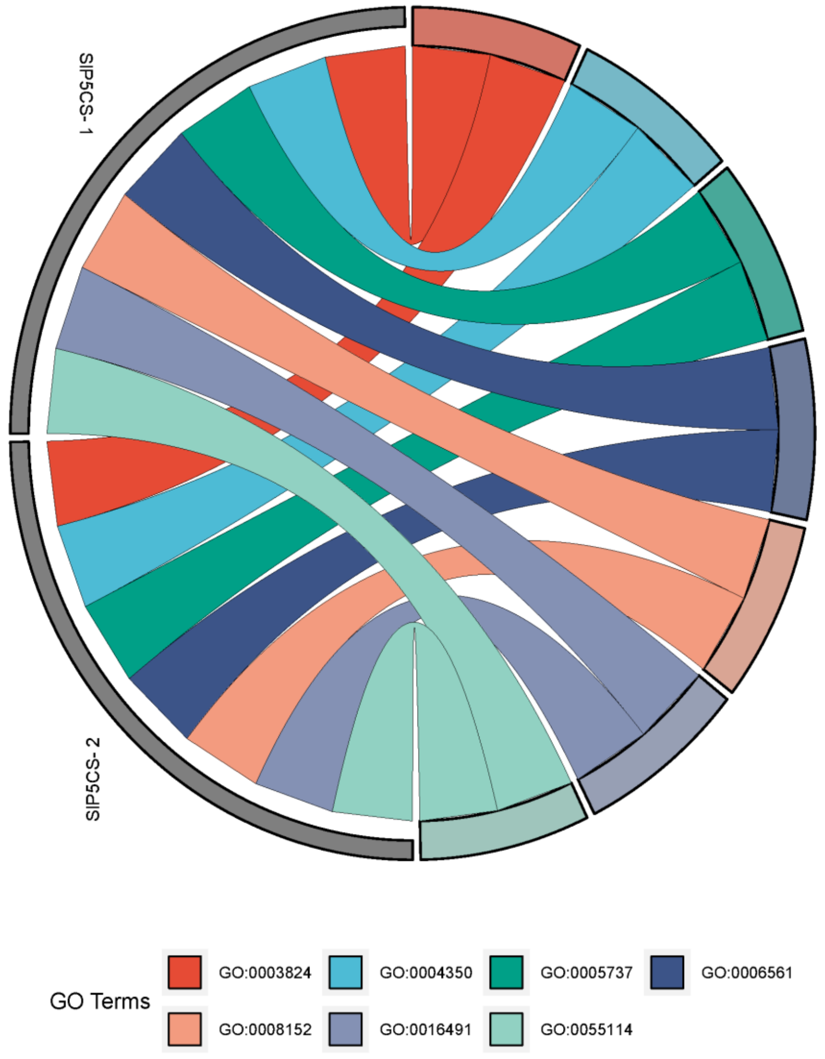

Fig. S53. GO terms for *P5CS* genes

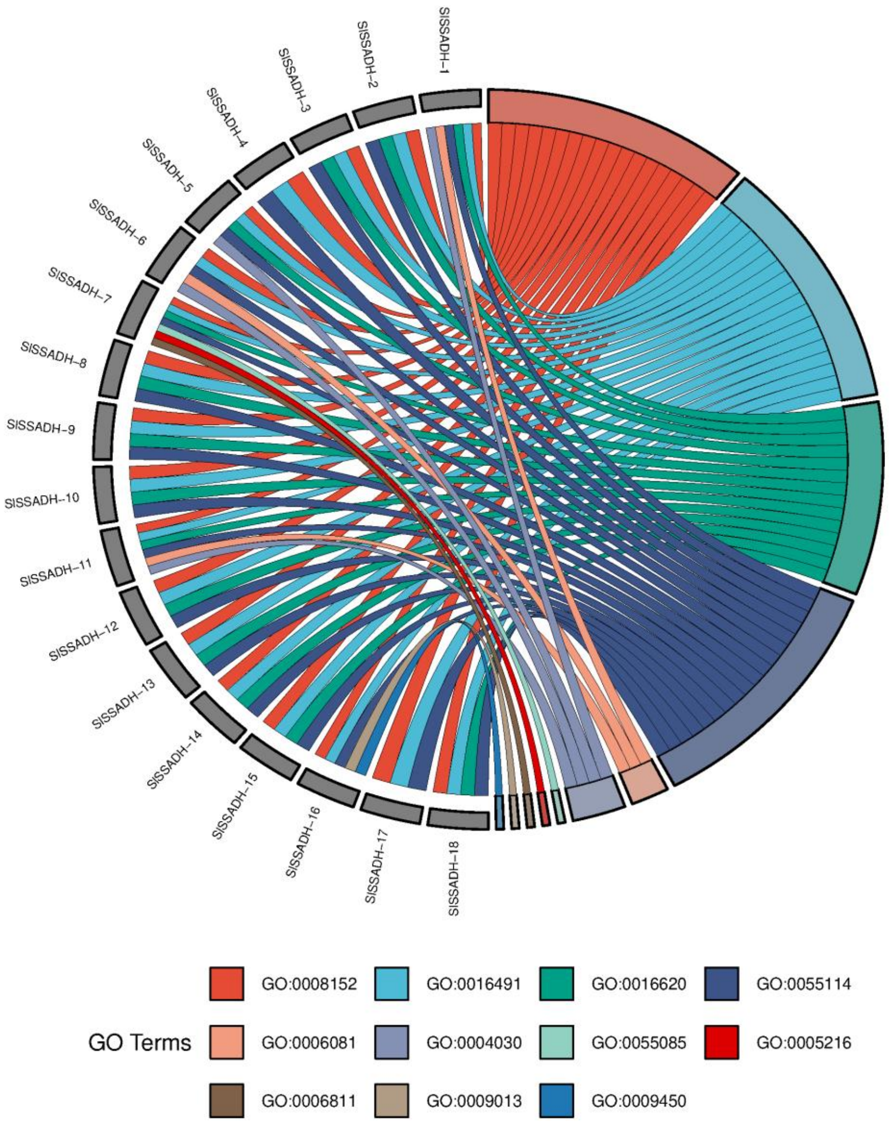

Fig. S54. GO terms for *SSADH* genes

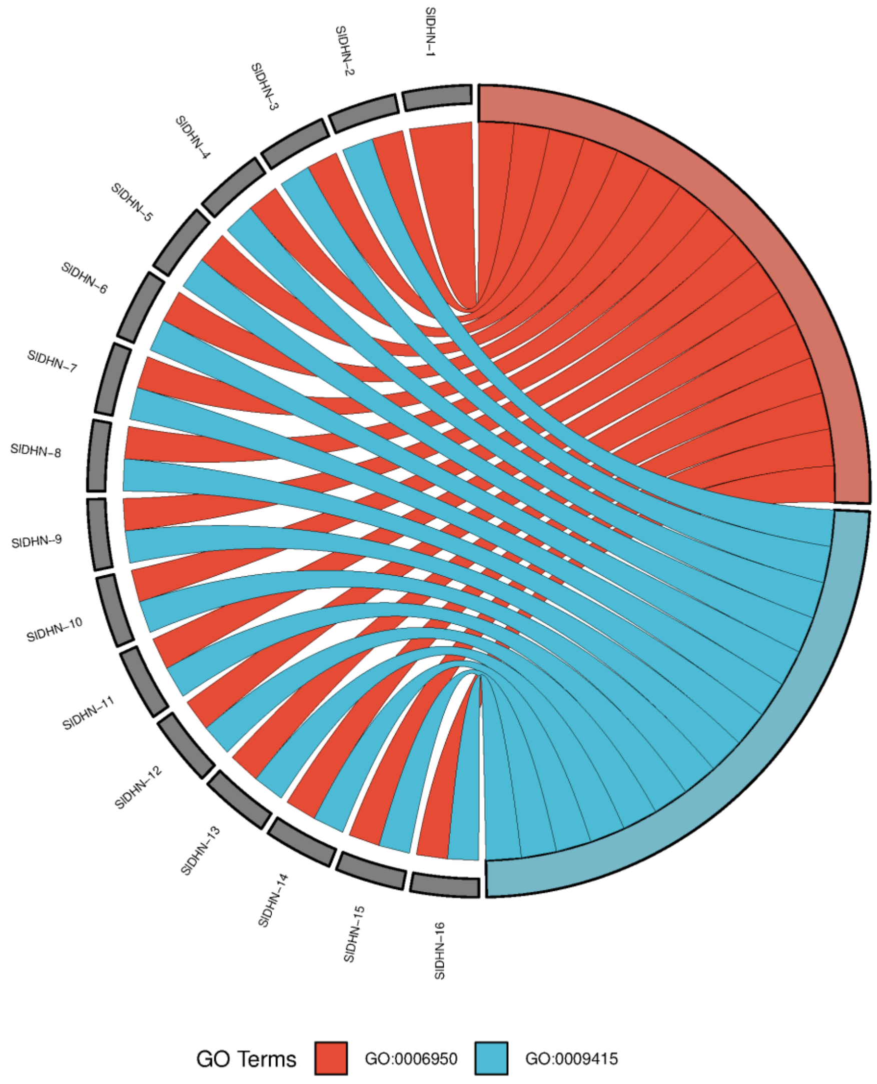

Fig. S55. GO terms for *DHN* genes

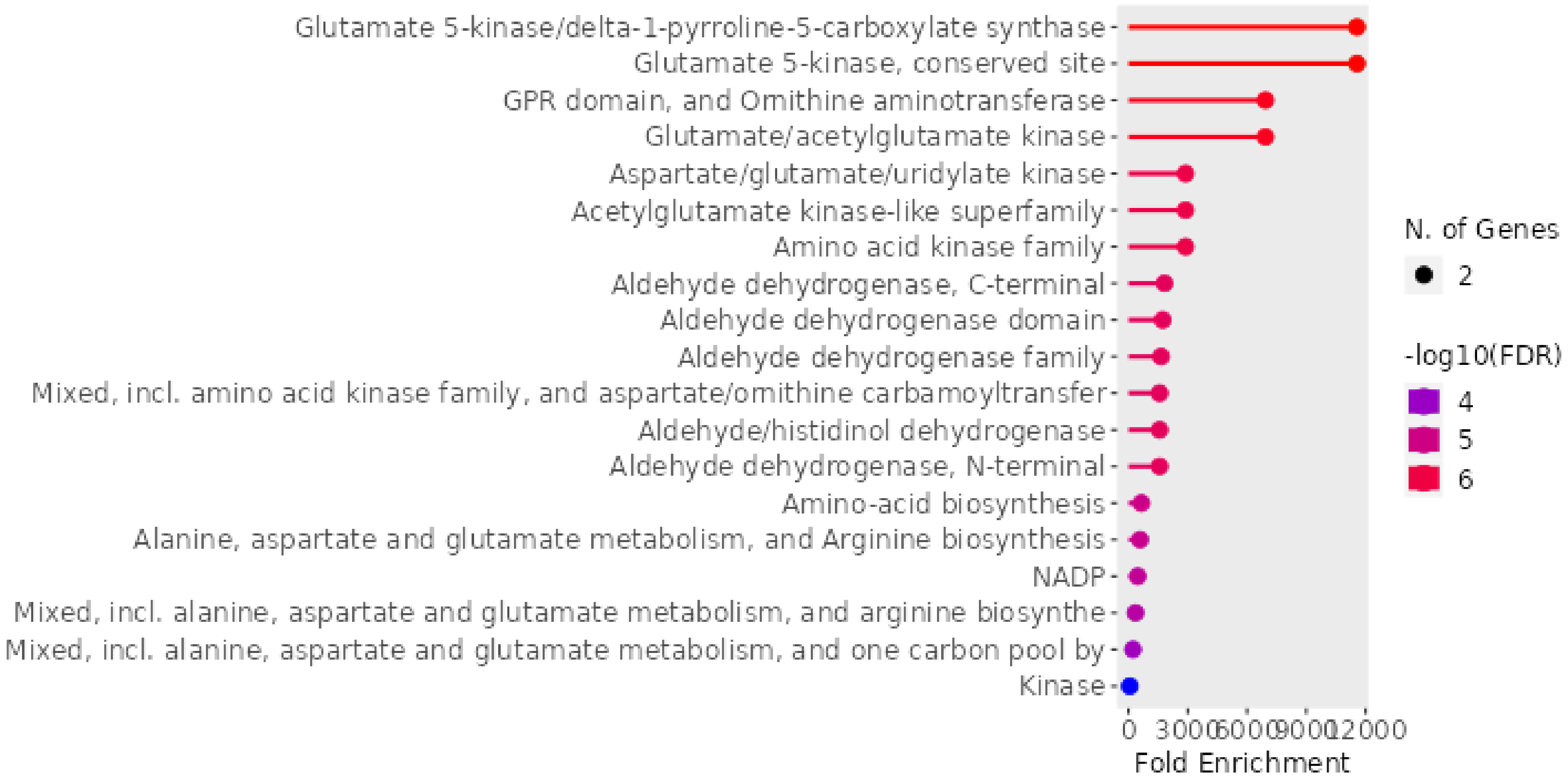

Fig. S56. Gene ontology enrichment analysis were confirmed the functional role of *P5CS* as a stress responsive

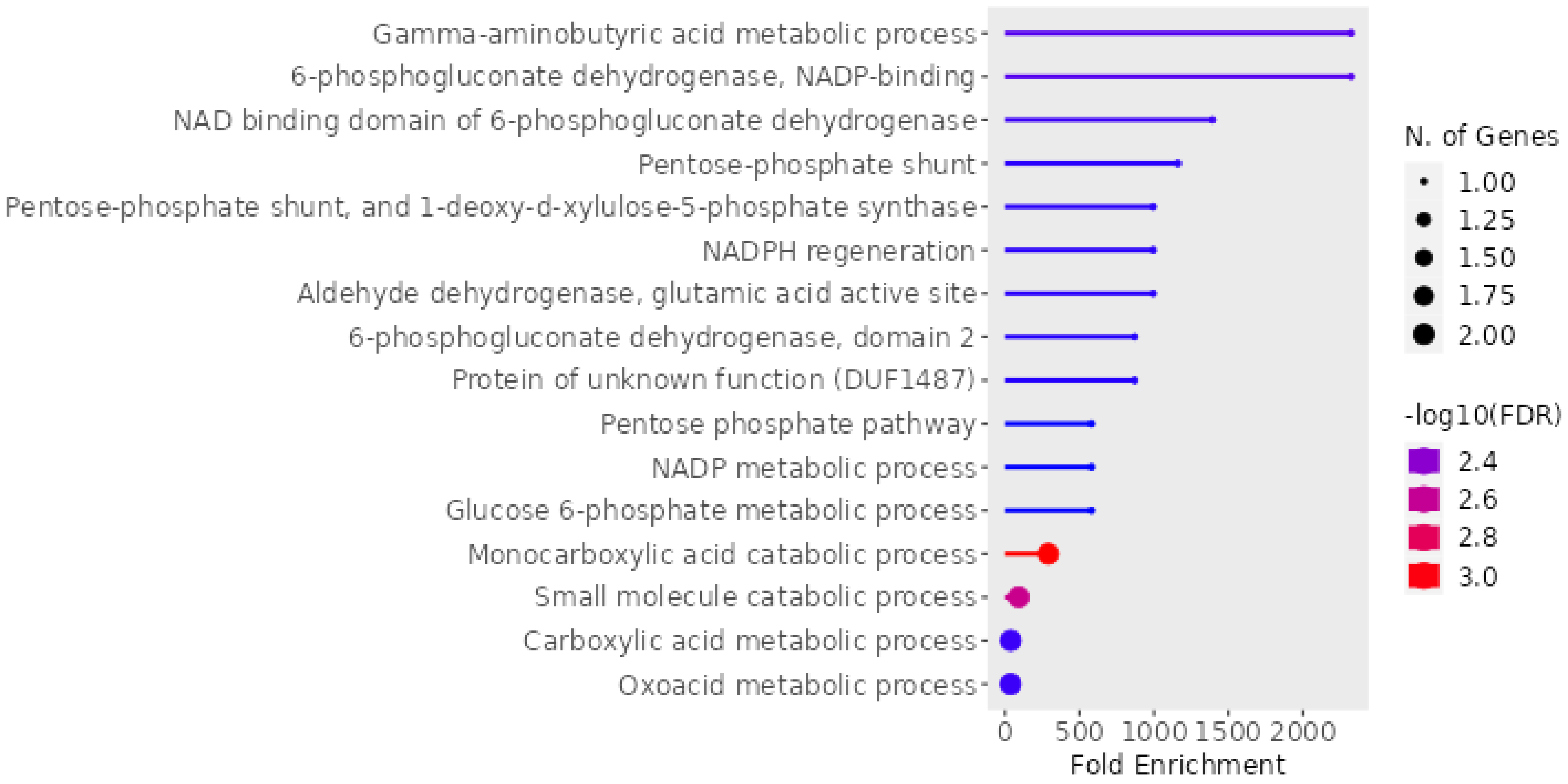

Fig. S57. Gene ontology enrichment analysis were confirmed the functional role of *SSADH* as a stress responsive

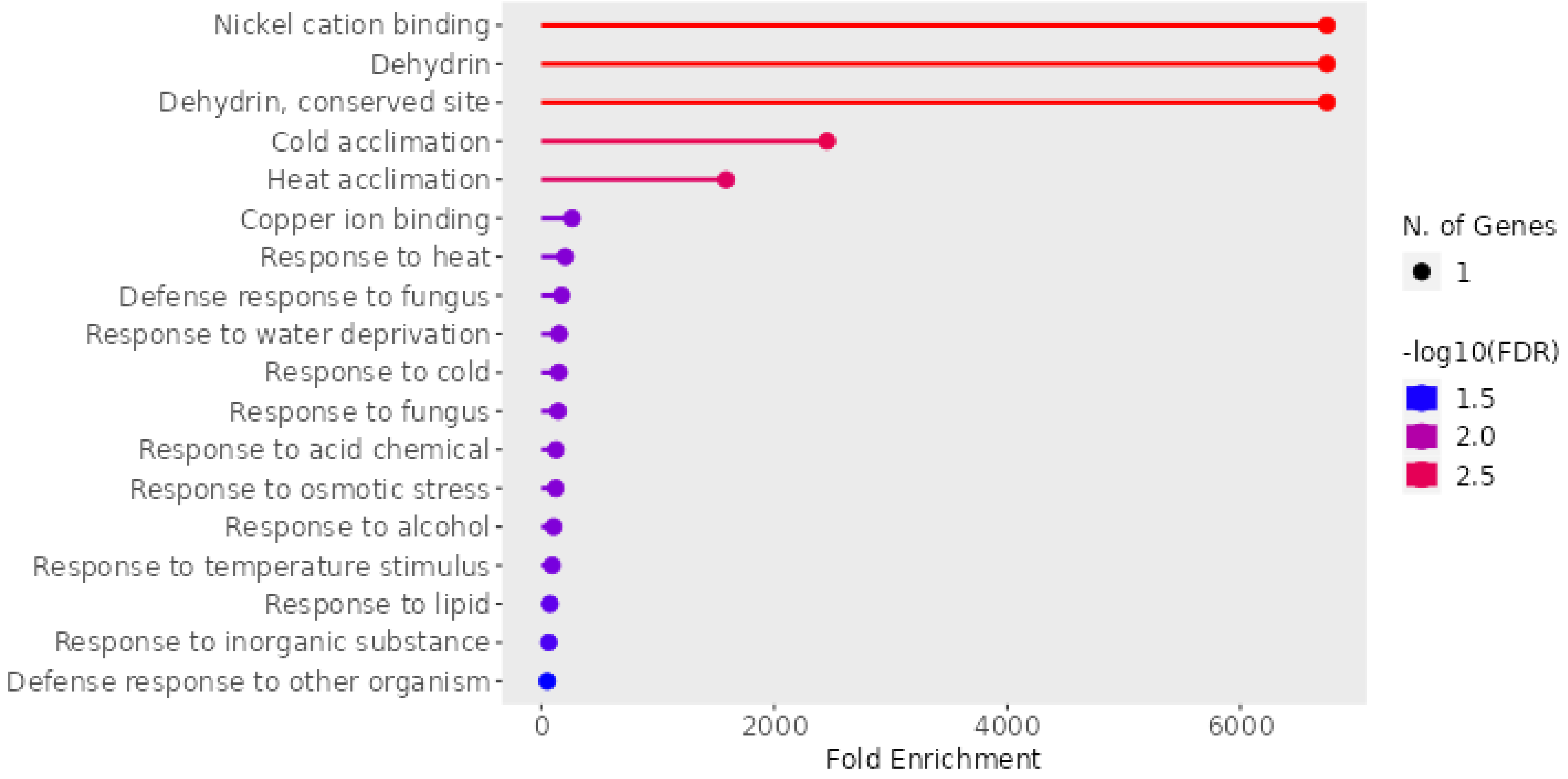

Fig. S58. Gene ontology enrichment analysis were confirmed the functional role of *DHN* as a stress responsive
